# Metformin improves HPRT1-targeted purine metabolism and repairs NR4A1-mediated autophagic flux by modulating FoxO1 nucleocytoplasmic shuttling to treat postmenopausal osteoporosis

**DOI:** 10.1038/s41419-024-07177-5

**Published:** 2024-11-06

**Authors:** Keda Yang, Xiaochuan Wang, Chi Zhang, Dian Liu, Lin Tao

**Affiliations:** Department of Orthopedics, First Hospital of China Medical University, Shenyang, China

**Keywords:** Mechanisms of disease, Bone, Osteoporosis

## Abstract

Osteoporosis is a major degenerative metabolic bone disease that threatens the life and health of postmenopausal women. Owing to limitations in detection methods and prevention strategy awareness, the purpose of osteoporosis treatment is more to delay further deterioration rather than to fundamentally correct bone mass. We aimed to clarify the pathogenesis of postmenopausal osteoporosis and optimize treatment plans. Our experiments were based on previous findings that oxidative stress mediates bone metabolism imbalance after oestrogen deficiency. Through energy metabolism-targeted metabolomics, we revealed that purine metabolism disorder is the main mechanism involved in inducing oxidative damage in bone tissue, which was verified via the use of machine-learning data from human databases. Xanthine and xanthine oxidase were used to treat osteoblasts to construct a purine metabolism disorder model. The activity and differentiation ability of osteoblasts decreased after X/XO treatment. Transcriptomic sequencing indicated that autophagic flux damage was involved in purine metabolism-induced oxidative stress in osteoblasts. Additionally, we performed serum metabolomics combined with network pharmacology to determine the pharmacological mechanism of metformin in the treatment of postmenopausal osteoporosis. HPRT1 was the potential target filtered from the hub genes, and FoxO1 signalling was the key pathway mediating the effect of metformin in osteoblasts. We also revealed that SIRT3-mediated deacetylation promoted the nuclear localization of FoxO1 to increase the expression of HPRT1. HPRT1 upregulation promoted purine anabolism and prevented the accumulation of ROS caused by purine catabolism to reverse oxidative damage in osteoblasts. We propose that purine metabolism disorder-induced oxidative stress is important for the pathogenesis of postmenopausal osteoporosis. The therapeutic mechanism of metformin should be confirmed through subsequent drug optimization and development studies to improve bone health in postmenopausal women.

## Introduction

Osteoporosis is a systemic bone metabolic disease characterized by decreased bone density and quality and destruction of the bone microstructure, which increase susceptibility to fractures. With the ageing of the population and changes in lifestyle, the incidence of osteoporosis has increased annually [1]. Owing to the insidious nature of osteoporosis, most patients are diagnosed with severe disease only after experiencing serious complications such as fractures or skeletal deformities. Postmenopausal women are at high risk for osteoporosis [2]. As oestrogen levels decrease, its inhibitory effect on osteoclast activity gradually weakens. Bone loss continues leads to osteoporosis when the bone resorption rate exceeds the bone formation rate. At present, treatment for osteoporosis relies mainly on oral drugs that act directly on osteoblasts and osteoclasts and have limited effects [3]. Hormone replacement therapy is not favoured because of its limited applicability and significant side effects. Therefore, elucidating the pathogenesis of postmenopausal osteoporosis and analysing the fundamental reason for the imbalance in bone metabolism caused by decreased oestrogen are important ways to optimize treatment methods and design targeted drugs.

Oxidative stress is considered an important mediator in the development of postmenopausal osteoporosis [4]. A lack of oestrogen weakens the metabolic activity of the female body, causing slow excretion of toxic substances and accelerating ageing. The ageing process is induced mainly by oxidative stress-induced damage accompanied by the accumulation of reactive oxygen species (ROS) [5]. Oestrogen is also a reducing hormone, and the antioxidant capacity decreases when oestrogen is deficient [6]. Clinical studies have shown changes in oxidative-related biomarkers, including antioxidant enzymes and advanced oxidation products, in the serum, which indicates a highly oxidative state in postmenopausal women with osteoporosis [7]. Mitochondria are the main organelles in cells that regulate redox balance. When the mitochondrial ATP supply is abnormal, the damaged electron transfer chain increases the production of ROS, such that the ROS load exceeds the mitochondrial clearance capacity, which causes mitochondrial damage and a vicious cycle of energy metabolism abnormalities [8]. Aerobic glycolysis and calcium homoeostasis in mitochondria provide energy and activate osteogenesis in the differentiation and mineralization of osteoblasts. Redox imbalance of mitochondria negatively affects osteoblast survival and induces osteoblast apoptosis [9]. Low mitochondrial antioxidant activity and mitophagy impairment promote the expression of osteoclastogenic marker genes to promote bone resorption and erosion [10, 11]. Mitochondrial dysfunction leads to an imbalance of osteogenesis and osteoclastogenesis to accelerate the development of osteoporosis. Purines are an important class of metabolites that serve as the basis for nucleotides and constitute the genetic material and energy units of living organisms. The purine metabolism process is gradually decomposed into uric acid under the catalysis of xanthine oxidase (XO), accompanied by the generation of a large amount of ROS [12]. Antioxidant enzymes in mitochondria are not sufficient to remove excessive ROS, resulting in an intracellular peroxidation state. ROS accumulation in mitochondria inhibits the respiratory chain to decrease ATP production, reduce membrane permeability to induce cytochrome C-mediated cell apoptosis and block the expression of mitochondrial genes to decrease the number of mitochondria [13]. Bioinformatics analysis of metabolites has indicated that purine metabolism disorders are involved in the development of osteoporosis [14, 15]. Oestrogen plays an important role in the biosynthesis and excretion of purine metabolites by increasing the bioavailability of purine compounds and regulating the expression of receptors on the renal tubular epithelium [16]. A cohort study revealed that the serum uric acid level was associated with bone mineral density in females but not in males, which indicated the specific role of purine metabolism disorders in postmenopausal osteoporosis [17]. Oestrogen deficiency inhibits the activity of CD39 and CD37 to decrease the extracellular adenosine levels of primary osteoprogenitors and osteoclasts in the bone marrow, which indicates the pathological association of purine metabolism with postmenopausal osteoporosis [18]. Exploring the mechanism of purine metabolism disorder-induced oxidative damage in bone tissue contributes to clarifying the pathogenesis, revealing the target of action, and increasing the treatment efficacy for postmenopausal osteoporosis.

Metformin is an available anti-ageing drug that contributes to improving metabolism and lifespan [19]. Metformin can inhibit the production of ROS and ameliorate oxidative stress [20]. The antioxidant properties of metformin suggest its potential application in the prevention and treatment of postmenopausal osteoporosis. It was reported that metformin treatment contributed to decreasing the risk of osteoporosis and fractures and elevating bone mineral density at the forearm, femoral neck, and lumbar spine [21]. Our preliminary experiments confirmed that metformin could suppress bone loss and improve oxidative states in bilateral ovariectomized (OVX) mice [22]. Metformin enhances mitochondrial function and increases the activity of antioxidant enzymes to reverse oxidative damage in osteoblasts [23]. There is currently no evidence to suggest that metformin improves bone health by regulating purine metabolism. On the basis of the results obtained by our group showing that disordered purine metabolism is important for the pathogenesis of oxidative stress in osteoporosis, we explored the potential effect and regulatory mechanism of metformin on purine metabolism. Our experimental results elucidate the pharmacological effects of metformin and reveal effective targets for treating postmenopausal osteoporosis.

## Methods

### Reagents

MC3T3-E1 cells were purchased from the Cell Bank of Type Culture Collection of the Chinese Academy of Sciences. Xanthine (X7375) and xanthine oxidase (X1875) were purchased from Sigma Aldrich (St. Louis, MO, USA). Metformin (0830A) was purchased from Dalian Meilunbio (Dalian, China). Antibodies against p62 (1:10,000; cat. no. ab109012), LC3B (1:2000; cat. no. ab192890), caspase-3 (1:2,000; cat. no. ab184787), cleaved caspase-3 (1:5,000; cat. no. ab214430), Bcl-2 (1:2,000; cat. no. ab182858), Bax (1:2,000; cat. no. ab32503), cytochrome c (1:5,000; cat. no. ab133504), HPRT1 (1:5,000; cat. no. ab133242), acetyl-lysine (1:2,000; cat. no. ab190479), FoxO1 (1:2,000; cat. no. ab179450), and GAPDH (1:5,000; cat. no. ab8245) were purchased from Abcam. Antibodies against SIRT3 (1:5000; cat. no. 10099-1-AP) were purchased from Proteintech (Wuhan, China). Antibodies against NR4A1 (1:1000; cat. no. T56890s) were purchased from Abmart (Shanghai, China). Antibodies against β-tubulin (1:1000; cat. no. 2146S) were purchased from Cell Signalling Technology (Danvers, MA, USA).

### Animals and postmenopausal osteoporosis model

Eight-week-old C57BL/6J female mice were obtained from the Department of Laboratory Animal Science of China Medical University. All the mice were housed in a stable environment at 20–26 °C, 40–70% relative humidity, ≤14 mg/m^3^ ammonia, ≤60 dB noise and a 12 h light/dark cycle with ad libitum access to food and drinking water. Our animal experiments were approved by the Animal Ethics Committee of the First Affiliated Hospital of China Medical University (approval no. 2019014). All the experimental operations were carried out according to the laboratory and animal welfare guidelines of China Medical University. The mice were randomly divided into three groups (*n* = 7 per group): the sham group (the epidermis and peritoneum were cut and then sutured), the OVX group and the OVX+Met group. The mice in the OVX+Met group were intragastrically administered metformin (120 mg/kg/day, dissolved in 0.5 ml of saline). The mice in the other two groups were treated with an equal volume of saline. Bilateral ovariectomy was applied to mice to construct an OVX model, which is a mature method for directly reducing oestrogen levels. These mice were raised for two months under the above treatment methods. Isoflurane was used for inhalation anaesthesia with oxygen. The mouse bone tissue was divided into two parts: one fixed with 70% ethanol for bone structure imaging and bone parameter measurement and the other fixed with 10% formaldehyde for immunohistochemistry. Mouse blood was centrifuged to obtain serum samples, which were stored in a −80 °C freezer for subsequent experiments. The random assignment of experiments and samples was blinded.

### Microcomputed tomography (micro‑CT)

The mouse bones were removed from the alcohol storage solution and dried superficially on paper. Dry bone tissue was wrapped in plastic film and placed in a plastic/polystyrene foam tube, which was mounted horizontally in the SkyScan 1076/1176 scanner sample chamber, for micro-CT imaging (Bruker Corporation). The parameters of the scan were as follows: X-ray voltage, 50 kV; X-ray current, 200 µA; filter, 0.5 mm aluminium; image pixel size, 8.9 µm; camera resolution, high (4000 pixel field width); tomographic rotation, 180°/360°; rotation step, 0.3–0.5°; frame averaging, 1; and scan duration, 20–50 min. The reconstruction parameters were as follows: smoothing 0–1; beam hardening 33%; ring reduction 5; postalignment use value calculated by Nrecon; histogram limits min 0; and max at the right end of the high-density 'tail'. Region of interest selection: both trabecular and cortical were selected with reference to the growth plate. A crossectional slice was selected as a growth plate reference slice, in the following way. Moving slice-by-slice toward the growth plate from the metaphysis/diaphysis, a point is reached where a clear 'bridge' of low-density cartilage (chondrocyte seam) becomes established from one corner of the cross section to another. This bridge is established by the disappearance of the last band of fine primary spongiosal bone interrupting the chondrocyte seam. This landmark allows a reference level to be defined for the growth plate: trabecular and cortical volumes of interest are then defined relative to this reference level.

### Immunohistochemistry

Ten per cent ethylenediaminetetraacetic acid disodium (EDTA) (Sigma‒Aldrich) mixed with 1% sodium hydroxide (NaOH) (pH = 7.0) (Sigma‒Aldrich) was applied for decalcification of the bone tissues. The calcified bone was treated with gradient ethanol for dehydration and xylene for transparency. After paraffin embedding and slicing, the processed tissue slices were placed in a 63 °C constant-temperature oven and baked for 2 h for immunohistochemistry. After dewaxing and hydration, 0.01 M citric acid buffer (pH = 6.0) was added to the tissue slices, which were boiled for 15 min. After cleaning, 3% deionized water was used to inactivate endogenous peroxidase activity. The corresponding primary antibodies were added and incubated with the samples overnight at 4 °C. Then, immunohistochemistry detection reagents were sequentially added, and the samples were incubated at 37 °C for 20 min. A DBA kit was used for colour reactions. Haematoxylin QS was used as a counterstain. Before microscopic examination, routine dehydration, transparency, and film sealing were performed.

### Metabolomics analysis

A combination of ultrahigh-performance liquid chromatography (UHPLC) and liquid chromatography‒mass spectrometry (LC‒MS/MS) was applied for metabolomic detection of bone and serum. Targeted (energy) metabolomics detection of bone tissue and nontargeted metabolomic detection of serum samples: 1. Sample preparation: The samples were taken from the refrigerator and placed on ice (subsequent operations were performed on ice). Each sample was cut into a centrifuge tube, a steel ball was added, and the sample was homogenized twice at 30 Hz for 30 s each time. Fifty milligrams of the homogenized sample was accurately weighed into a new centrifuge tube, and 500 µl of 70% methanol/water (precooled at −20 °C) extraction solution was added. The samples were shaken at 2500 r/min for 5 min and allowed to stand for 5 min; this operation was repeated twice. The samples were subsequently centrifuged at 4 °C and 12,000 r/min for 5 min. The supernatants (400 μl) were aspirated into new centrifuge tubes. The supernatants were incubated at −20 °C for 30 min and centrifuged at 4 °C and 15,000 r/min for 20 min. Two hundred microlitres of the supernatant was used for analysis. 2. Ultraperformance liquid chromatography (ExionLC™ AD) and tandem mass spectrometry (QTRAP® 6500+) were used for data acquisition. The liquid phase conditions used were as follows: chromatographic column: ACQUITY UPLC BEH amide column (1.7 µm, 100 mm) × 2.1 mm i.d.); mobile phase: A phase, ultrapure water (10 mM ammonium acetate, 0.3% ammonia water); B phase, 90% acetonitrile/water (V/V); gradient elution procedure: A/B ratio of 5:95 (V/V) for 0–1.2 min, 30:70 (V/V) for 8 min, 50:50 (V/V) for 9.0–11 min, and 5:95 (V/V) for 11.1–15 min; flow rate of 0.4 mL/min; column temperature of 40 °C; and injection volume of 2 μL. The mass spectrometry conditions were as follows: the temperature of the electric spray ion source (ESI) was 550 °C, the mass spectrum voltage in positive ion mode was 5500 V, the mass spectrum voltage in negative ion mode was −4500 V, and the curtain gas (CUR) pressure was 35 psi. In the Q-Trap 6500+ each ion pair was scanned and detected on the basis of the optimized clustering potential (DP) and collision energy (CE).

### Data processing and analysis

The original file obtained from mass spectrometry detection was imported into Compound Discoverer 3.1 (CD3.1) software for spectrum processing and database searches to obtain qualitative and quantitative results for the metabolites. Quality control was subsequently performed on the data to ensure the accuracy and reliability of the results. Multivariate statistical analysis was conducted on the metabolites, including principal component analysis (PCA) and partial least squares discriminant analysis (PLS-DA), to reveal the differences in metabolic patterns among the different groups. Hierarchical clustering (HCA) and metabolite correlation analysis were used to reveal the relationships between samples and between metabolites. Finally, we assessed the biological significance of the metabolites through functional analysis and metabolic pathway analysis.

### Network pharmacology

Cytoscape 3.7.2 (Cytoscape Consortium) was used to construct the metabolite‒protein‒pathway network. The main metabolites and regulated proteins were examined to reveal the underlying pharmacological mechanism. The potential targets of osteoporosis were determined according to the results obtained by searching 'osteoporosis' in the Online Mendelian Inheritance in Man (OMIM, https://omim.org/), therapeutic target database (TTD, http://db.idrblab.net/ttd/) and genecards (https://www.gene-cards.org/). The targets of metformin were identified via searches against STITCH 5.0 (http://stitch.embl.de/), SwissTargetPrediction (http://www.swisstargetprediction.ch/), and ChEMBL (https://www.ebi.ac.uk/chembl/). The intersection of the two targets obtained above was considered a potential target for the treatment of osteoporosis with metformin. The enrichment analysis of these targets included protein-protein interaction (PPI) network, gene ontology (GO) enrichment and KEGG pathway analyses.

### Machine learning

We obtained the GSE51615 and GSE100609 datasets, which contain gene expression information on postmenopausal osteoporosis patients and healthy controls from public databases. The selected genes were included in the study because of their critical roles in purine metabolism. The expression data of these genes were extracted and processed using the R language, and shared genes were identified in the two datasets to ensure the consistency of the results. We used the ‘mlr3verse’ package to construct a machine-learning model and conducted multiple K-fold cross-validations on each model. We used indicators such as the AUC, accuracy, and Brier score to evaluate model performance; we also used box plots and ROC curves to evaluate model performance. The models were compared. On the basis of the performance evaluation, we selected the Kernel K-Nearest Neighbours (KKNN) model and independently validated it on a second dataset to determine the accuracy and robustness of the model. The KKNN model is an extended version of the KNN algorithm. The KKNN model generalizes the KNN model to more complex decision boundaries by introducing a kernel function, such as the Gaussian kernel or other kernel functions. The introduction of the kernel function can help the KKNN model better handle nonlinear data distributions, increasing the performance of the model when dealing with complex data. The data-driven approach adopted in this study has the potential to provide new insights into the prediction of osteoporosis.

### Cell culture

MC3T3-E1 cells were cultured in α-Minimal Essential Medium (Procell, Wuhan, China) supplemented with 10% foetal bovine serum (Procell, Wuhan, China), 100 U/ml streptomycin sulphate and 100 mg/ml penicillin. Osteogenic induction medium was purchased from Cyagen (Guangzhou, China). The osteogenic medium was replenished every other day. The cells were grown in a humidified incubator with 5% CO_2_ at 37 °C. Xanthine and xanthine oxidase (X/XO) were used to mimic the disruption of purine metabolism in osteoblasts, and metformin was added for 48 h of treatment. A full dose of xanthine was dissolved in 1 M NaOH and added to the medium. Then, xanthine oxidase was added to the medium at concentrations of 0.01, 0.1, 1, and 10 munits/ml with or without 200 mM metformin.

### Cell viability assay

A Cell Counting Kit-8 (CCK-8) (C0038, Beyotime, Shanghai, China) was used to detect cell viability after adding different concentrations of XO. The cells were plated in 96-well plates with 5 replicate wells per condition. Once the cells adhered and grew steadily, media supplemented with different concentrations of XO were applied. After 24, 48, and 72 h, 10 µl of CCK-8 reagent was added to each well to determine cell viability. The absorbance at 450 nm was measured using an enzyme-labelling instrument (BioTex, ELx808, Vermont, USA). The cell survival rate was calculated using the following formula: [(OD value in the experimental group—OD value in the blank group)/(OD value in the control group—OD value in the blank group)] × 100%.

### Apoptosis assay by flow cytometry

Different concentrations of X/XO were added to cell culture flasks containing MC3T3-E1 cells and incubated at 37 °C for 48 h. The cells were then harvested, resuspended in binding buffer and stained with FITC-Annexin V/propyl iodide via an Annexin V-FITC kit (C1062L, Beyotime, Shanghai, China) for 30 min in the dark at room temperature. The percentage of apoptotic cells was detected via FACScan flow cytometry (BD Biosciences) and analysed via CytExpert 2.3 (Beckman, Miami, USA).

### Measurement of intracellular ROS levels

The cells were plated in 6-well plates and treated with different concentrations of X/XO for 48 h. The oxidation-sensitive probe dichlorodihydrofluorescein (S0033S, DCFH-DA, Beyotime, Shanghai, China) was added to the osteoblasts, after which the cells were finally imaged under an inverted fluorescence microscope (Nikon, TI-PS100W/A, Japan) after a 30 min incubation.

### Measurement of the mitochondrial membrane potential

The mitochondrial membrane potential of MC3T3-E1 cells following the above treatment was assessed by using a mitochondrial membrane potential assay kit containing JC-1 (C2006, Beyotime, Shanghai, China). The cells were incubated in JC-1 staining working solution in a humidified incubator at 37 °C for 30 min, followed by three washes with JC-1 buffer. Relative changes were evaluated via flow cytometry and analysed with CytExpert 2.3. Absolute changes were measured using a multifunctional microplate reader. The fluorescence of JC-1 was recorded at 585/590 nm (red) and 510/527 nm (green) excitation/emission wavelengths. The red/green ratio was calculated to indicate the mitochondrial membrane potential.

### Alkaline phosphatase (ALP) activity detection

A BCIP/NBT Alkaline Phosphatase Colour Development Kit (C3206, Beyotime, Shanghai, China) was used to detect ALP levels. ALP is secreted by osteoblasts, and its activity can directly reflect the degree of differentiation of osteoblasts. We induced MC3T3-E1 cells to proliferate in osteogenic medium with or without X/XO treatment for 14 days. Then, ALP activity was assessed according to the manufacturer’s instructions.

### Alizarin red S staining

Changes in calcium salt concentration can indicate the proliferation and differentiation of osteoblasts. Alizarin Red S can recognize calcium salts by chelating their components with tissue and cell calcium salts, producing orange-red sediment. MC3T3-E1 cells were cultured in a 6-well plate, treated with osteogenic induction medium for 21 days, and then stained with Alizarin Red S. First, we washed the cells three times with PBS, fixed them with 4% paraformaldehyde for 20 min, and then washed them three times with distilled water. Then, 0.1% Alizarin Red Hydrogen Trichloride (pH 8.3) was added to the cells, which were allowed to stand at room temperature for 2 h.

### Nuclear and cytoplasmic protein extraction

A Nuclear and Cytoplasmic Protein Extraction Kit (P0028, Beyotime, Shanghai, China) was used to extract nuclear and cytoplasmic proteins. The cells were treated with cytoplasmic protein extraction reagents A and B, vortexed and incubated in an ice bath. The supernatant was collected as the cytoplasmic protein fraction. The cell pellets were subsequently incubated with nuclear protein mixture, vortexed, placed in an ice bath and centrifuged at 4 °C at 12,000 × *g* for 10 min. The supernatant was collected as the nuclear protein.

### Western blotting

The cells were lysed via RIPA buffer containing a mixture of benzoyl fluoride and phosphatase inhibitors from Beyotime (P0013B, Shanghai, China). Afterwards, the lysate was centrifuged at 12,000 × *g* for 30 min at 4 °C. The protein concentration was determined via a BCA protein concentration assay kit (P0010S, Beyotime, Shanghai, China), and each sample was adjusted to a concentration of 3 µg/µl in RIPA and sampling buffers. The protein samples were stored in a −20 °C freezer. The proteins were subsequently separated via SDS‒PAGE and transferred to a polyvinylidene fluoride (PVDF) membrane (Millipore, Burlington, USA). The membranes containing proteins of different molecular weights were then incubated in blocking buffer for 1.5 h, washed with 1% TBST, and then incubated with a primary antibody at 4 °C overnight, followed by incubation with a secondary antibody at 4 °C the next day. After thorough cleaning, the protein bands were treated with a luminescent solution and visualized using a chemiluminescence (ECL) system (UVP Inc., CA, United States). The protein levels were normalized to those of β-actin (molecular weight 43 kDa), tubulin-β (molecular weight 55 kDa) and GAPDH (molecular weight 37 kDa); finally, the optical density and relative protein expression were calculated via ImageJ software.

### Measurement of the intracellular calcium concentration

The intracellular calcium concentration in MC3T3-E1 cells following treatment was assessed using a calcium ion fluorescence probe containing Fluo-4 AM (S1061S; Beyotime, Shanghai, China). The cells were incubated in a humidified incubator at 37 °C in Fluo-4 AM working solution for 60 min and then washed three times with PBS. Relative changes were assessed via flow cytometry and analysed via CytExpert 2.3. The cells were finally imaged under an inverted fluorescence microscope.

### Coimmunoprecipitation (Co-IP)

The cells were collected and then lysed in lysis buffer supplemented with proteinase inhibitor on ice. After centrifugation, the cell supernatant was collected, and protein samples were obtained. An antibody against acetyl-lysine was combined with protein A/G magnetic beads (295420, MedChem Express, Monmouth Junction, USA) to form the antibody‒magic bead complex after washing with TBST. The complex was mixed with the protein samples, rotated and incubated at 4 °C for 2 h, followed by washing 4 times with TBST to separate the magic beads. The proteins were subsequently separated via SDS‒PAGE for western blotting to analyse the acetylation level of FoxO1.

### Transcriptome sequencing

MC3T3-E1 cells were divided into 3 groups: the control group, the X/XO treatment group and the X/XO and metformin treatment group. After these treatments, we obtained the cells from the culture flasks and counted 10 million cells. The cells were quickly frozen in liquid nitrogen. Messenger RNA (mRNA) extraction, cDNA synthesis, PCR enrichment, library construction, quality control and sequencing were performed by the Novogene Institute (Tianjin, China). DESeq2 (1.20.0) was used to analyse differential gene expression between the two groups, with 3 biological replicates per condition. The clusterProfiler R package was used to enrich the GO of differentially expressed genes (DEGs) and rectify the differences in gene length. Following correction, GO terms with *p* values less than 0.05 were deemed significantly enriched in the DEGs. GO enrichment analysis was performed with DAVID Bioinformatics Resources 6.8 to analyse the genes whose expression significantly differed between the control group and the X/XO-treated group and between the X/XO-treated group and both the X/XO-treated and metformin-treated groups. The differentially enriched MF, CC and BP terms are shown. The enrichment of the marker genes was assessed via the KEGG pathway database in the clusterProfiler R package, after which the biological significance of the genes, as well as key pathways that influence specific biological processes, was revealed under different experimental conditions.

### Virus titre determination

The 293 A cell suspension was prepared with DMEM containing 5% FBS, and 11 mL of a 1 × 10^5^/mL cell suspension was prepared for each plate. Cells (100 μL per well with 1 × 10^4^ cells) were added to two 96-well plates. For columns 11 and 12 of the 96-well plates, 100 μL of DMEM supplemented with 5% FBS was added to each well to create a negative control. The A‒H rows of the 96-well plates were set up successively, and 100 μL of medium was added to each row to mark 8 continuous gradient dilution sample solutions. The 96-well plates were cultured in a CO_2_ incubator at 37 °C for 10 days. The cytopathic effect should occur within 10 days. On day 10, the cytopathic effect of each well was observed under a microscope, and the number of positive wells in each row was recorded and compared with that of the negative control. For a 100 μL sample, titre T = 10^1+d(s-0.5)^ ; d = log_10_ dilution = 1 (for 10 times dilution) s = sum of positive ratios (from the first 10 times dilution); TCID50/mL conversion to PFU/mL: T = a × 10^b^TCID50/mL = a × 10^b-0.7^PFU/mL. The titre value obtained from two repeated experiments should differ less than 10^0.7^.

### Cell transmission electron microscopy

The cells were removed from the culture bottle, centrifuged, the supernatant was removed, electron microscopy fixative was added, the mixture was resuspended at 4 °C, and the mixture was fixed for 4 h. The fixed cells were centrifuged, the supernatant was discarded, and 0.1 M phosphate-buffered saline (PB) (pH 7.4) was added.

The mixture was mixed and rinsed for 3 min and then centrifuged and washed 3 times. A 1% agarose solution was prepared, and the cell sample was embedded in lipid sugar. One percent osmium acid was prepared with 0.1 M phosphate buffer (pH 7.4), and the mixture was fixed at room temperature in the dark for 2 h.

The mixture was rinsed 3 times with phosphate-buffered saline (pH 7.4) for 15 min each. At room temperature, the tissue was dehydrated with graded alcohol for 20 min each time and then with 100% acetone twice for 15 min each time. A pure 812 embedding agent was used to infiltrate the sample at 37 °C for 8 h. Pure 812 embedding agent was added to the embedding plate, the sample was inserted into the embedding plate, and the mixture was incubated overnight at 37 °C. The embedding board was placed in a 60 °C oven for 48 h of polymerization, and the resin block was removed. An ultrathin slicer was used to slice the resin blocks, which were ultrathin 60–80 times. A 150 mesh square film of copper mesh was used for fishing. A 2% uranyl acetate-saturated alcohol solution was used to stain the copper mesh in the dark for 8 min. The samples were washed with ultrapure water three times. The samples were incubated with 2.6% lead citrate solution in the absence of carbon dioxide for 8 min, washed with ultrapure water three times and dried with filter paper. The copper mesh slices were placed in a copper mesh box and dried at room temperature overnight. Images were obtained and collected for analysis via transmission electron microscopy (TEM) in the research building of China Medical University.

### mRFP-GFP-LC3 puncta assay

The cells were cultured in 35 mm glass bottom Petri dishes and then transfected with mRFP-GFP-LC3 adenovirus (HB-AP210 000; HanBio Technology, Shanghai, China) via the 1/2 small volume infection method for 12 h. The cells were subsequently treated with X/XO and metformin. Following treatment, mRFP-GFP-LC3 puncta were observed under a confocal microscope (Nikon, AXR, Japan). The mRFP tag was used to label and track LC3. Reduced GFP expression indicates the fusion of lysosomes and autophagosomes to form autophagolysosomes. The decrease in GFP fluorescence was due to a pH change, resulting in only red fluorescence being detectable at this stage. After microscopic imaging, the red and green fluorescence images were merged. Yellow spots were identified as autophagosomes (RFP + GFP + ), whereas red dots were considered autophagolysosomes (RFP + GFP−). The intensity of the autophagic flux was evaluated by quantifying the number of yellow and red dots.

### Cell transfection

HPRT1 and NR4A1 small interfering RNAs (siRNAs) were purchased from Santa Cruz Biotechnology (CA, USA). Then, 36 μl of siRNA duplex was added to 250 μl of siRNA transfection medium to form solution A, and 36 μl of siRNA transfection reagent was added to 250 μl of siRNA transfection medium to form solution B. Solutions A and B were vortexed and incubated for 30 min at room temperature. After each transfection, solution A, solution B and 2 ml of siRNA transfection medium were added to a cell culture flask containing 4 × 105 cells and incubated at 37 °C for 6 h. Subsequently, the transfection medium was changed to fresh cell culture medium and incubated for 24 h. Then, drugs were added to the medium for the next experiment.

### Lentiviral activation particle transduction

HPRT lentiviral activation particles were purchased from Santa Cruz Biotechnology (CA, USA). The lentiviral particles were added to a cell culture flask containing 80% confluent cells and incubated overnight. The flask contained complete medium supplemented with 5 μg/ml polybrene (sc-134220, Santa Cruz). The cells were selected with puromycin and hygromycin B until resistant colonies were identified. A stable HPRT-overexpressing cell strain was subsequently obtained.

### Statistical analysis

The experimental results were analysed and plotted via GraphPad Prism 9.0 statistical software. Each experiment was repeated three times. Normally distributed data are represented by the mean ± standard deviation, and nonnormally distributed data are represented by the median and upper and lower quartiles. For normally distributed data, the *t*-test was used to compare the data between two groups, and one-way analysis of variance was used to compare the data between multiple groups. A *P* value < 0.05 after correction was considered to indicate statistical significance.

## Results

### Bone energy metabolism-targeted metabolomics and machine learning

**Fig. 1:**
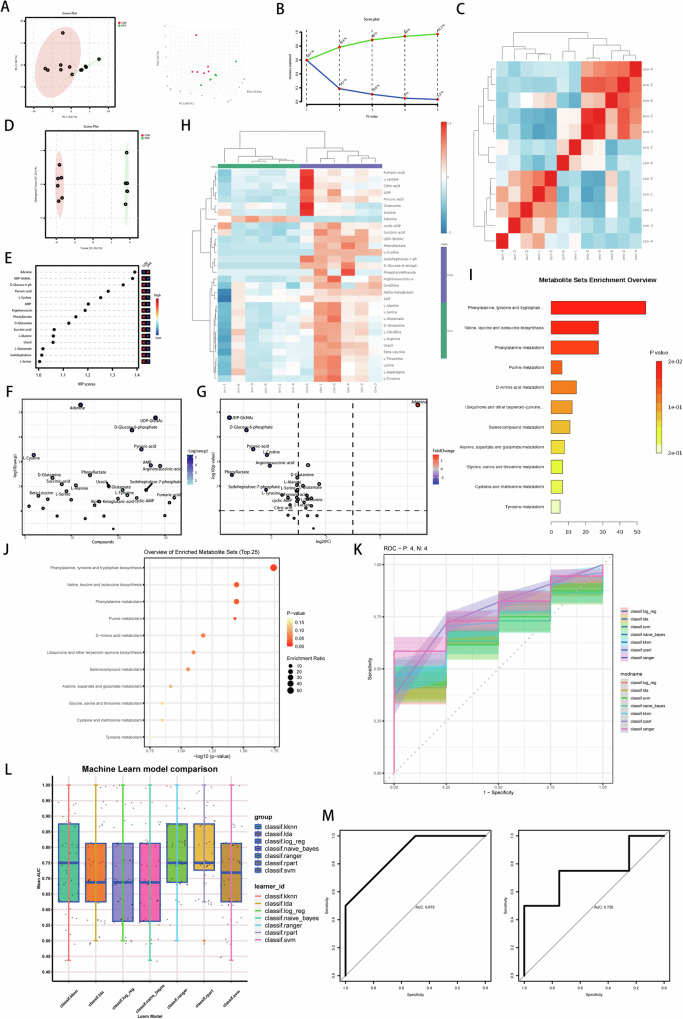
Bone energy metabolism-targeted metabolomic and machine learning. **A** 2D and 3D Principal Component Analysis (PCA) of sham and OVX samples. **B** Grouped Principal Component Analysis (PCA) of sham and OVX samples. **C** Repeated correlation assessment of sham and OVX samples. **D**, **F**, **G** Orthogonal Partial Least Squares Discriminant Analysis (OPLS-DA). **E** Differential Metabolite Bar Chart between sham and OVX samples. **H** Differential clustering heatmap analysis of differential metabolites between sham and OVX samples. **I**, **J** Differential metabolite KEGG functional annotation of differential metabolites. **K** ROC curve of machine semester with databases. **L** Machine Learn model comparison. **M** ROC curves of relevant machine learning models in (**K**) and (**L**).

We conducted energy metabolism-targeted metabolome detection on femoral bone samples from 6 mice in each group in the sham group and OVX group (Fig. 1A, B). However, there were significant differences between the sham operation group and the ovariectomy group. We then performed repeated correlation analyses (Fig. 1C), and the results demonstrated that the identification of differentially abundant metabolites in these samples was highly reliable. The results of the cluster analysis revealed significant differences in purine metabolism-related metabolites between these two groups. To further increase the sensitivity of the analysis, we used least partial squares discriminant analysis (OPLS-DA) (Fig. 1D). Overall, 31 effective differentially abundant metabolites were screened (Fig. 1E–G, Table 1). We statistically analysed the relative differential contributions of the differentially abundant metabolites through multiple algorithms and generated a heatmap of the differentially abundant metabolites (Fig. 1H). On the basis of previous results, the KEGG annotations of the significant differentially abundant metabolites were classified according to the pathway types in the KEGG database. As shown in Fig. 1I, J, purine metabolism significantly differed between these two groups. Purine-related metabolites accumulated in the OVX group. To verify the above conclusion, key genes in the purine metabolism pathway were used as models. We applied machine learning methods to evaluate the possibility of using the expression patterns of purine metabolism-related genes to predict bone quality and mass in postmenopausal women on the basis of relevant data from postmenopausal osteoporosis patients in public databases and loose diagnostic models. After comparing the performance of multiple machine learning models, we found that the KKNN and SVM models showed outstanding performance in predicting osteoporosis, which was evident from the average AUC of each model in the box plot (Fig. 1K, L). The average AUC values of these models on the GSE51615 and GSE100609 datasets were mostly distributed between 0.6 and 0.9. Further ROC curve analysis indicated that the KKNN and SVM models exhibited high sensitivity and a low false-positive rate, the naive Bayes model performed well in the low false-positive rate interval but then declined rapidly, and the Ranger and Rpart models overall exhibited poor performance. With respect to the independent dataset GSE100609, the verification results of the KKNN model further highlighted its excellence through receiver operating characteristic (ROC) curve analysis, with an area under the curve (AUC) value of 0.875 (Fig. 1M). These results indicate that purine metabolism-related genes have high diagnostic value for distinguishing postmenopausal osteoporosis patients from healthy controls.

**Table 1:** 1.1 List of differential metabolite contribution values from least discriminant squares analysis in bone tissue energy metabolism-targeted analysis results. 1.2 The differential metabolite clustering heat map was obtained using least discriminant squares analysis in the sham operation group and OVX group.

1.1	
Metabolite	oplsVIP
Adenine	1.515666974
ADP	0.492363443
Alpha-Ketoglutaric-acid	0.529404787
AMP	1.352323955
Argininosuccinic-acid	0.768840284
Beta-Leucine	0.81690218
Citric-acid	1.031178042
cyclic-AMP	1.13232056
D-Glucose-6-phosphate	1.554822175
D-Glutamine	0.917285772
Fumaric-acid	0.612383251
Guanosine	0.101659914
Inosine	0.767346307
L-Alanine	0.945691168
L-Arginine	0.756372672
L-Asparagine	0.623786114
L-Citrulline	0.705729606
L-Cystine	1.306309443
L-Glutamate	0.843025387
L-Lactate	1.002846062
L-Serine	0.8630749
L-Threonine	0.857799521
L-Tyrosine	1.07057544
Lysine	0.767541959
Ornithine	0.898561848
Phenyllactate	1.379055089
Pyruvic-acid	1.455703503
Sedoheptulose-7-phosphate	1.098634568
Succinic-acid	1.135227191
UDP-GlcNAc	1.139237698
Uracil	0.948696008

1.2							
Index	Compounds	Class	VIP	*P*-value	Fold_Change	Log2FC	Type
Adenine	Adenine	Nucleotide metabolomics	1.51566697	0.00026147	10.4048	3.3792	up
Phenyllactate	Phenyllactate	Organic Acid And Its Derivatives	1.37905509	0.012613774	0.0927	−3.4313	down
Citric-acid	Citric acid	Amino Acid metabolomics	1.03117804	0.079982943	0.4062	−1.2997	down
L-Lactate	L-Lactate	Organic Acid And Its Derivatives	1.00284606	0.05608305	0.4826	−1.0511	down
L-Tyrosine	L-Tyrosine	Amino Acid metabolomics	1.07057544	0.033289697	0.3317	−1.592	down
cyclic-AMP	cyclic-AMP	Nucleotide metabolomics	1.13232056	0.035249414	0.3855	−1.3752	down
Pyruvic-acid	Pyruvic acid	Amino Acid metabolomics	1.4557035	0.002543321	0.1592	−2.6511	down
AMP	AMP	Nucleotide metabolomics	1.35232396	0.005884348	0.6418	−0.6398	down
UDP-GlcNAc	UDP-GlcNAc	Nucleotide metabolomics	1.1392377	0.00032683	0.1281	−2.9647	down

### Integrated analysis of metabolomics and network pharmacology

**Fig. 2:**
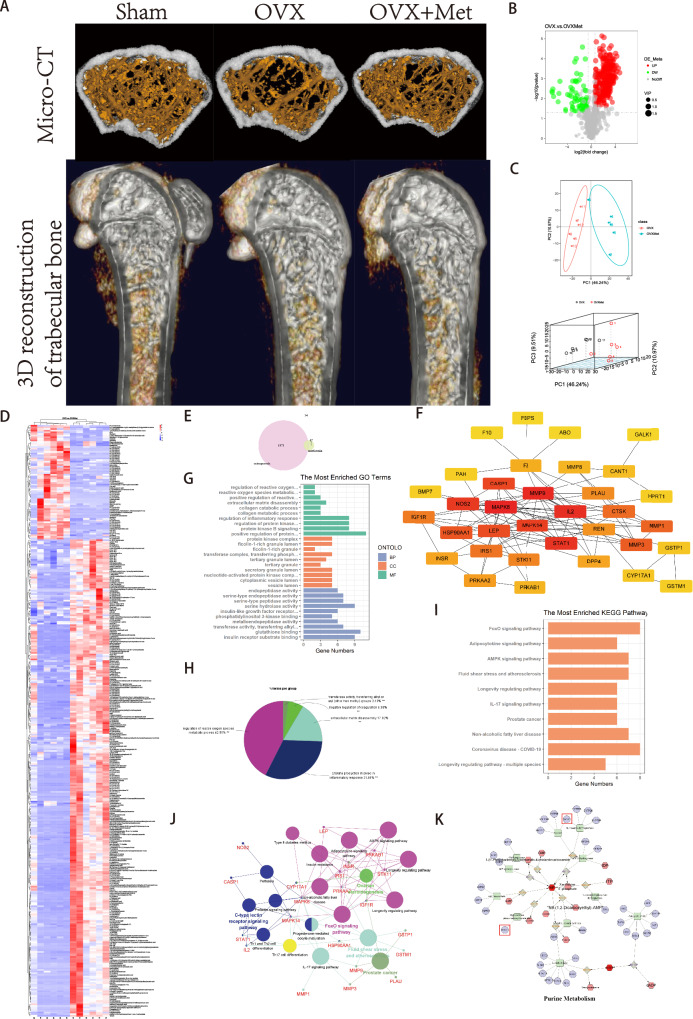
Integrated analysis of metabolomics and network pharmacology. **A** Micro-CT analysis and 3D reconstruction of mice bone tissue. **B** Volcano plot of serum differential metabolites in OVX and metformin treatment groups. **C** 2D and 3D total sample PCA analysis of samples. **D** Heatmap of all of differential metabolite in OVX and metformin treatment samples. **E** Venn diagram of the intersection of hub targets for metformin and osteoporosis. **F** PPI network of hub genes in (**E**). **G** GO function enrichment of hub genes. **H** Proportion of enrichment functions. **I** KEGG pathway analysis of hub genes. **J** Network analysis of pathways enriched in (**I**). **K** Targets screening based on interaction analysis of metabolomics and network pharmacology.

To determine the therapeutic effect of metformin in the treatment of osteoporosis, OVX mice were intragastrically administered metformin for two months. The micro-CT results indicated that metformin could prevent bone loss and improve the bone microstructure in OVX mice (Fig. 2A and S1A). We performed a serum metabolomics analysis to determine the differentially abundant metabolites between the OVX group and the OVX with metformin treatment group (Fig. 2B, Table 2). The differences between the samples of these two groups were visualized via multimodal PCA (Fig. 2C). To further increase the sensitivity of the analysis, we used least partial squares discriminant analysis (OPLS-DA) (Fig. S1B). The differences in metabolites were visualized via a cluster heatmap (Fig. S1C). All of the differentially abundant metabolites are listed in Fig. 2D according to their contents. Additionally, we performed network pharmacology analysis to explore the pharmacological mechanism and potential targets of metformin. After collection from the databases, we obtained 1871 associated targets for osteoporosis and 47 associated targets for metformin (Fig. 2E). After taking the intersection, a total of 34 genes were identified as hub targets for metformin treatment of osteoporosis. The PPI network of these 34 genes is presented in Fig. 2F. We also performed GO enrichment and KEGG pathway analyses of the hub genes. Gene function was analysed from three aspects, namely, molecular function (MF), biological process (BP), and cellular component (CC), and the top ten terms were identified (Fig. 2G). After GO analysis, a total of five functional groups were identified according to the MF analysis (Fig. 2H). The genes were ranked in order of proportion as follows: regulation of reactive oxygen species metabolic process (42.55%), cytokine production involved in the inflammatory response (31.91%), extracellular matrix disassembly (17.02%), negative regulation of coagulation (6.38%) and transferase activity, transferring alkyl or aryl (other than methyl) groups (2.13%). KEGG pathway analysis also revealed the top ten relevant pathways, including the FoxO signalling pathway, adipocytokine signalling pathway, AMPK signalling pathway, fluid shear stress and atherosclerosis, longevity regulating pathway, IL-17 signalling pathway, prostate cancer, nonalcoholic fatty liver disease, coronavirus disease-COVID-19, and longevity regulating pathway-multiple species (Fig. 2I). The KEGG network is shown in Fig. 2J. Additionally, interaction analysis via metabolomics and network pharmacology indicated that purine metabolism plays an important role in the effect of metformin on osteoporosis. Differential metabolites were imported into the MetScape plugin in Cytoscape to collect the compound-reaction-enzyme-gene networks. By matching the potential targets identified in network pharmacology with the genes in MetScape analysis, we revealed that HPRT1 and CANT1 were the hub genes mentioned above. The affected pathway is purine metabolism (Fig. 2K).

**Table 2:** Heat map of differential metabolite clustering between OVX group and metformin group.

ID	Name	Formula	Molecular Weight	FC	log2FC	*P*-value	ROC	VIP	Up_Down
Com_8696_pos	17beta-Trenbolone	C18 H22 O2	270.1614	3.273451	1.710813	7.21E-06	1	1.225943	up
Com_8887_pos	PC (8:0/8:0)	C24 H48 N O8 P	509.313	4.168854	2.059651	8.55E-06	1	1.454958	up
Com_740_pos	N-[2-chloro-6-(trifluoromethoxy)phenyl]-2,2-dimethylpropanamide	C12 H13 Cl F3 N O2	295.0589	1.730923	0.791541	1.42E-05	1	1.382145	up
Com_8717_pos	PC (9:0/16:2)	C33 H62 N O8 P	631.4225	3.829303	1.937082	2.26E-05	1	1.279703	up
Com_3048_pos	Carbaprostacyclin	C21 H34 O4	332.2351	9.499103	3.247791	2.28E-05	1	1.396035	up
Com_4686_pos	N-(1-benzylpiperidin-4-yl)-5-methylthieno[2,3-d]pyrimidin-4-amine	C19 H22 N4 S	338.1556	0.350516	−1.51245	2.61E-05	1	1.365419	down
Com_19969_pos	5-[(2-hydroxybenzylidene)amino]-2-(2-methoxyethoxy)benzoic acid	C17 H17 N O5	315.1146	7.764488	2.956891	3.05E-05	1	1.385932	up
Com_9550_pos	PC (5:0/13:1)	C26 H50 N O8 P	535.3284	2.599021	1.377968	3.10E-05	1	1.473482	up
Com_8964_pos	Coenzyme Q2	C19 H26 O4	318.1811	3.341192	1.740363	3.49E-05	1	1.343371	up
Com_19993_pos	2-Methoxyestradiol (2-MeOE2)	C19 H26 O3	302.1881	2.618802	1.388907	4.18E-05	1	1.347742	up
Com_193_pos	α-Eleostearic acid	C18 H30 O2	278.2247	4.165767	2.058582	4.57E-05	1	1.431064	up
Com_7337_pos	Tetrahydroaldosterone	C21 H32 O5	364.226	16.0517	4.004654	5.40E-05	1	1.36022	up
Com_1684_pos	2,4-dihydroxyheptadec-16-en-1-yl acetate	C19 H36 O4	350.2457	7.980518	2.996482	6.25E-05	1	1.359723	up
Com_154_pos	9-Oxo-10(E),12(E)-octadecadienoic acid	C18 H30 O3	294.2195	5.358208	2.421751	6.43E-05	1	1.421208	up
Com_7855_pos	PC (14:1/14:1)	C36 H68 N O8 P	673.4688	9.19536	3.200906	6.45E-05	1	1.38905	up
Com_2934_pos	13,14-Dihydro prostaglandin E1	C20 H36 O5	338.2444	2.785055	1.477706	6.67E-05	1	1.428898	up
Com_11485_pos	Deflazacort	C25 H31 N O6	441.2139	8.043996	3.007912	6.80E-05	1	1.399617	up
Com_11391_pos	Linalool	C10 H18 O	154.1358	5.465796	2.450432	6.93E-05	1	1.412754	up
Com_465_pos	5-OxoETE	C20 H30 O3	318.2174	2.926607	1.549229	7.48E-05	1	1.427816	up
Com_1904_pos	PC (16:2/18:5)	C42 H70 N O8 P	365.2216	18.16962	4.183456	7.50E-05	1	1.414713	up
Com_15327_pos	DL-3,4-Dihydroxyphenyl glycol	C8 H10 O4	170.0586	3.844683	1.942865	9.07E-05	1	1.385451	up
Com_1837_pos	13(S)-HOTrE	C18 H30 O3	294.2192	4.517604	2.175558	9.10E-05	1	1.411092	up
Com_3152_pos	Methyl dihydrojasmonate	C13 H22 O3	226.1571	10.68007	3.416849	9.61E-05	1	1.415966	up
Com_11469_pos	PC (8:0/22:6)	C38 H64 N O8 P	693.4264	5.606867	2.487195	9.70E-05	1	1.348596	up
Com_5715_pos	Homo-Gamma-Linolenic Acid (C20:3)	C20 H34 O2	306.256	3.996967	1.998906	9.77E-05	1	1.436862	up
Com_6007_pos	PC (17:0/17:0)	C42 H84 N O8 P	761.5833	0.172458	-2.53568	9.81E-05	1	1.411479	down
Com_18944_pos	Dl-3-Hydroxy-kynurenine	C10 H12 N2 O4	224.0797	3.508118	1.810697	0.000101	1	1.381551	up
Com_2251_pos	4,4’-dimethoxy[1,1’-biphenyl]-2-carbonitrile	C15 H13 N O2	239.0924	0.488941	−1.03227	0.000103	1	1.396226	down
Com_2796_pos	16-Heptadecyne-1,2,4-triol	C17 H32 O3	306.2197	13.16203	3.71831	0.000104	1	1.349358	up
Com_2007_pos	Prostaglandin K2	C20 H30 O5	332.1968	10.75203	3.426538	0.00011	1	1.399593	up
Com_12472_pos	PC (18:4e/18:4)	C44 H74 N O7 P	759.506	6.177946	2.627127	0.000111	1	1.408564	up
Com_682_pos	9-Oxo-ODE	C18 H30 O3	294.2194	8.312346	3.055256	0.000113	1	1.413416	up
Com_2054_pos	13-HPODE	C18 H32 O4	312.23	5.204181	2.379671	0.00012	1	1.396803	up
Com_2257_pos	ACar 18:0	C25 H50 N O4	427.3668	1.609115	0.686268	0.000126	1	1.384449	up
Com_10569_pos	beta-Estradiol 17-Acetate	C20 H26 O3	314.1884	6.514567	2.703669	0.000131	1	1.416167	up
Com_4555_pos	11-Deoxy prostaglandin F1α	C20 H36 O4	362.2436	11.88347	3.570884	0.000133	1	1.397843	up
Com_7482_pos	PC (11:0/12:0)	C31 H62 N O8 P	607.4219	8.538761	3.094027	0.000141	1	1.357413	up
Com_16129_pos	O-Acetyl-L-carnitine	C9 H17 N O4	203.1161	3.892584	1.960728	0.000142	1	1.385186	up
Com_10920_pos	PC (18:2e/16:3)	C42 H76 N O7 P	737.5393	0.14542	−2.78171	0.000144	1	1.422114	down
Com_289_pos	(±)12(13)-DiHOME	C18 H34 O4	296.2354	6.014889	2.588538	0.000149	0.972222	1.386897	up
Com_3344_pos	PC (18:4/18:4)	C44 H72 N O8 P	773.4861	6.439822	2.687021	0.00018	1	1.150699	up
Com_5729_pos	20-Carboxy-Leukotriene B4	C20 H30 O6	366.2045	5.465821	2.450438	0.000186	1	1.371734	up
Com_18024_pos	Aplaviroc hydrochloride	C33 H43 N3 O6	577.3201	7.697015	2.944299	0.00019	0.972222	1.33556	up
Com_7037_pos	4-methoxy-6-[2-(4-methoxyphenyl)ethyl]-2H-pyran-2-one	C15 H16 O4	260.1028	11.30101	3.49838	0.00019	1	1.35817	up
Com_15826_pos	1-(4-benzylpiperazino)-2-(pyridin-2-ylamino)propan-1-one	C19 H24 N4 O	346.1759	9.45115	3.24049	0.000202	1	1.404057	up
Com_8437_pos	T-2 Toxin	C24 H34 O9	488.2033	10.94591	3.45232	0.000205	1	1.340603	up
Com_9923_pos	4-Hydroxyretinoic Acid	C20 H28 O3	316.2042	3.862548	1.949553	0.00021	1	1.354743	up
Com_19153_pos	1,5-diphenyl-1H-1,2,4-triazole-3-thiol	C14 H11 N3 S	291.0228	6.637026	2.730537	0.000216	1	1.408027	up
Com_8800_pos	2-({2-[(3-methyl-5-cinnolinyl)amino]-2-oxoethyl}sulfanyl)acetic acid	C13 H13 N3 O3 S	269.0904	10.22979	3.354704	0.000217	1	1.368731	up
Com_1624_pos	Spinosyn A	C41 H65 N O10	731.4729	16.95145	4.083337	0.000218	0.972222	1.370318	up
Com_14787_pos	N-(2,3-dihydro-1H-inden-2-yl)-4-hydroxybutanamide	C13 H17 N O2	219.1264	11.92411	3.57581	0.000222	0.972222	1.354703	up
Com_9010_pos	1,3-Dihydro-1,3,3-trimethyl-2H-indol-2-ylidene acetaldehyde	C13 H15 N O	201.1156	13.81228	3.78788	0.000227	0.972222	1.313979	up
Com_3711_pos	PC (20:3e/22:5)	C50 H86 N O7 P	843.6	8.285878	3.050655	0.000233	1	1.385366	up
Com_9195_pos	2-(Formylamino)Benzoic Acid	C8 H7 N O3	165.0429	6.490867	2.698411	0.000242	1	1.373002	up
Com_17330_pos	L-Tyrosinemethylester	C10 H13 N O3	195.0899	4.830036	2.272034	0.000243	1	1.358721	up
Com_10311_pos	2,3-Dinor-11β-prostaglandin F2α	C18 H30 O5	348.1929	8.558479	3.097354	0.000245	1	1.415278	up
Com_11650_pos	PC (17:1/18:5)	C43 H74 N O8 P	763.5162	4.18045	2.063658	0.000247	1	1.26525	up
Com_15347_pos	Isobutyryl carnitine	C11 H21 N O4	231.1476	7.119564	2.831789	0.000261	0.972222	1.365782	up
Com_17749_pos	WQH	C22 H27 N7 O5	469.2092	9.404916	3.233415	0.000263	0.972222	1.309412	up
Com_11157_pos	3-(3,4-Dihydroxyphenyl)-2-Methylalanine	C10 H13 N O4	211.0849	4.613671	2.205915	0.000265	1	1.360394	up
Com_6777_pos	Spectinomycin	C14 H24 N2 O7	364.1868	5.317971	2.410876	0.000274	1	1.367442	up
Com_8641_pos	PC (11:0/11:0)	C30 H60 N O8 P	593.406	5.595777	2.484338	0.000275	1	1.373543	up
Com_4464_pos	2-[(carboxymethyl)(methyl)amino]-5-methoxybenzoic acid	C11 H13 N O5	239.0798	7.261695	2.860306	0.000285	1	1.38439	up
Com_6469_pos	1-(3-ethyl-2,4-dihydroxy-6-methoxyphenyl)butan-1-one	C13 H18 O4	220.1103	4.325689	2.11293	0.000291	0.972222	1.352676	up
Com_5387_pos	Estrone	C18 H22 O2	287.1849	21.91218	4.453661	0.000298	1	1.382495	up
Com_1763_pos	Palmitoleic Acid	C16 H30 O2	276.2092	7.267058	2.861371	0.0003	1	1.368694	up
Com_5944_pos	PC (16:2/17:2)	C41 H74 N O8 P	739.5161	6.632335	2.729517	0.000305	1	1.34902	up
Com_17190_pos	Colchicine	C22 H25 N O6	399.1669	7.675922	2.94034	0.000313	1	1.369251	up
Com_7283_pos	2-[6-(1H-benzo[d]imidazol-2-yl)-2-pyridyl]-1H-benzo[d]imidazole	C19 H13 N5	311.1138	14.43357	3.851356	0.000328	0.972222	1.339537	up
Com_11711_pos	PC (18:5/22:6)	C48 H74 N O8 P	823.529	7.014228	2.810284	0.000329	1	1.388204	up
Com_1755_pos	Prostaglandin H1	C20 H34 O5	319.2013	6.974118	2.802011	0.000331	1	1.343062	up
Com_13910_pos	ethyl 4-[(6-methyl-3-pyridazinyl)oxy]benzoate	C14 H14 N2 O3	258.1007	3.84384	1.942548	0.000332	0.972222	1.347523	up
Com_3415_pos	(2 R,3S,4S,5 R,6 R)-2-(hydroxymethyl)-6-(2-phenylethoxy)oxane-3,4,5-triol	C14 H20 O6	266.1156	1.620512	0.69645	0.000333	1	1.30961	up
Com_240_pos	Acetyl-L-carnitine	C9 H17 N O4	203.1159	1.657959	0.729408	0.000333	1	1.188141	up
Com_12199_pos	Normorphine	C16 H17 N O3	271.121	9.515883	3.250338	0.000337	0.972222	1.315179	up
Com_14506_pos	Oxymatrine	C15 H24 N2 O2	264.1842	11.44578	3.516744	0.000345	1	1.378404	up
Com_5863_pos	(+)-ar-Turmerone	C15 H20 O	216.1517	4.007327	2.00264	0.000346	0.972222	1.284878	up
Com_1832_pos	PC (20:4e/22:6)	C50 H82 N O7 P	839.5682	13.25664	3.728643	0.000361	1	1.286511	up
Com_18257_pos	PC (5:0/5:0)	C18 H36 N O8 P	425.2189	7.969679	2.994522	0.000368	1	1.377289	up
Com_4736_pos	N-Desmethylclobazam	C15 H11 Cl N2 O2	286.0508	4.111914	2.03981	0.00041	0.972222	1.271363	up
Com_3432_pos	PC (10:0/11:0)	C29 H58 N O8 P	579.3907	8.37031	3.065281	0.000412	1	1.356873	up
Com_14850_pos	2-(3,4-dimethoxyphenyl)ethanamine	C10 H15 N O2	181.1107	4.614427	2.206152	0.00042	0.972222	1.318088	up
Com_3953_pos	N1-(2-amino-2-oxoethyl)-2-(isopropylthio)acetamide	C7 H14 N2 O2 S	212.0591	18.03152	4.172449	0.000427	1	1.382663	up
Com_10652_pos	Methyl 3-indolyacetate	C11 H11 N O2	189.0787	2.420343	1.275211	0.000438	0.972222	1.299233	up
Com_15124_pos	N-Acetyl-L-tyrosine	C11 H13 N O4	223.0847	4.543832	2.183909	0.000439	0.972222	1.314862	up
Com_19894_pos	SQH	C14 H22 N6 O6	392.1485	5.608164	2.487529	0.00044	0.972222	1.250572	up
Com_12226_pos	SM (d15:3/28:1)	C48 H91 N2 O6 P	400.3366	3.952615	1.982807	0.000475	1	1.05495	up
Com_955_pos	PC (19:2/19:2)	C46 H84 N O8 P	809.5948	3.79214	1.923012	0.000497	1	1.338817	up
Com_422_pos	1,2-dihydroxyheptadec-16-yn-4-yl acetate	C19 H34 O4	348.2279	25.51669	4.673369	0.000503	1	1.375617	up
Com_11649_pos	Lysopc 18:2	C26 H50 N O7 P	519.3336	1.616529	0.692899	0.000506	1	1.364832	up
Com_18546_pos	1-(4-hydroxyphenyl)propane-1,2-diol	C9 H12 O3	190.0606	6.930372	2.792933	0.000511	0.972222	1.252217	up
Com_12391_pos	Bisphenol A	C15 H16 O2	228.1148	2.971926	1.571398	0.000513	1	1.329681	up
Com_13818_pos	5-Hydroxytryptophol	C10 H11 N O2	177.0792	5.555528	2.473924	0.000532	1	1.360863	up
Com_4432_pos	1-phenyl-5-(2-thienyl)-1H-1,2,4-triazol-3-ol	C12 H9 N3 O S	243.0512	11.73803	3.553118	0.000539	0.972222	1.336977	up
Com_5988_pos	3b,7b-Dihydroxy-5-androsten-17-one	C19 H28 O3	304.2039	3.299141	1.722091	0.00054	1	1.040978	up
Com_1799_pos	Daidzein	C15 H10 O4	254.0583	0.182154	-2.45677	0.00055	0.972222	1.365137	down
Com_412_pos	Avocadyne 1-acetate	C19 H34 O4	308.2353	14.65282	3.873107	0.000559	0.972222	1.323587	up
Com_4126_pos	Decanoylcarnitine	C17 H33 N O4	315.2415	2.10904	1.076586	0.000562	1	1.344493	up
Com_1251_pos	PC (22:6e/17:2)	C47 H80 N O7 P	801.5529	1.876538	0.908074	0.00057	1	1.343272	up
Com_7850_pos	8,15-Dihete	C20 H32 O4	336.2301	3.576735	1.838643	0.000583	1	1.359746	up
Com_1323_pos	Urocanic acid	C6 H6 N2 O2	138.0431	2.655994	1.409252	0.000598	1	1.3849	up
Com_4324_pos	5-Methoxyindoleacetic acid	C11 H11 N O3	205.0743	2.776568	1.473303	0.000619	1	1.364908	up
Com_3163_pos	PC (18:4e/16:4)	C42 H70 N O7 P	731.4745	8.9861	3.167695	0.000623	1	1.324178	up
Com_6595_pos	Glycitein	C16 H12 O5	284.0691	0.290421	-1.78378	0.000627	0.944444	1.340886	down
Com_13998_pos	2-(3,4-dimethoxyphenyl)-N-(4-morpholinophenyl)acetamide	C20 H24 N2 O4	356.1736	7.863909	2.975247	0.000642	0.944444	1.308928	up
Com_1761_pos	2,4-Dimethylbenzaldehyde	C9 H10 O	134.0733	1.604285	0.68193	0.000669	1	1.363533	up
Com_3775_pos	20-Dihydro 6α-methylprednisone	C22 H30 O5	374.2076	5.802405	2.536651	0.000683	1	1.361065	up
Com_3137_pos	4-Pyridoxic acid	C8 H9 N O4	183.0536	6.984147	2.804084	0.000694	1	1.369883	up
Com_11756_pos	PC (4:0/4:0)	C16 H32 N O8 P	397.1876	10.48315	3.390001	0.000696	1	1.373086	up
Com_6110_pos	KMH	C17 H30 N6 O4 S	374.2102	6.619067	2.726628	0.00071	1	1.315648	up
Com_5084_pos	Levodopa	C9 H11 N O4	197.0693	8.22688	3.040345	0.000719	1	1.35335	up
Com_5573_pos	13,14-dihydro-15-keto Prostaglandin A2	C20 H30 O4	356.1968	5.692499	2.509062	0.000723	1	1.320243	up
Com_5939_pos	8,8-dimethyl-2-phenyl-4H,8H-pyrano[2,3-h]chromen-4-one	C20 H16 O3	304.1065	8.581931	3.101302	0.00073	0.972222	1.337153	up
Com_3757_pos	PC (17:2/16:4)	C41 H70 N O8 P	735.4694	8.690249	3.119397	0.00074	1	1.348801	up
Com_1588_pos	PC (9:0/9:0)	C26 H52 N O8 P	537.3442	12.6889	3.665495	0.000753	1	1.37821	up
Com_11851_pos	Avobenzone	C20 H22 O3	332.1381	11.94788	3.578682	0.000755	1	1.307408	up
Com_9249_pos	PC (17:2/16:3)	C41 H72 N O8 P	737.4853	6.362315	2.669552	0.000789	1	1.373204	up
Com_9705_pos	2-Phenylpropionic acid	C9 H10 O2	150.0683	3.48119	1.799581	0.000791	0.972222	1.242439	up
Com_8461_pos	PC (9:0/18:5)	C35 H60 N O8 P	653.4179	5.877567	2.555219	0.000794	0.972222	1.316754	up
Com_7261_pos	methyl 3,4,5-trihydroxycyclohex-1-ene-1-carboxylate	C8 H12 O5	188.0689	2.392887	1.258752	0.000796	1	1.390851	up
Com_6726_pos	PC (20:4e/16:3)	C44 H76 N O7 P	761.5219	5.914851	2.564342	0.000803	0.972222	1.202759	up
Com_16705_pos	Metanephrine	C10 H15 N O3	197.1057	1.51721	0.601421	0.000806	0.972222	1.396158	up
Com_1176_pos	Desoxycortone	C21 H30 O3	330.2171	14.54666	3.862616	0.000812	0.972222	1.322283	up
Com_11540_pos	PC (7:0/22:6)	C37 H62 N O8 P	679.4085	3.509375	1.811214	0.000813	1	1.38542	up
Com_11730_pos	6 β-Hydroxycortisol	C21 H30 O6	378.2053	3.709022	1.891039	0.000816	0.972222	1.290855	up
Com_411_pos	(11E,15Z)-9,10,13-trihydroxyoctadeca-11,15-dienoic acid	C18 H32 O5	350.2074	12.6167	3.657263	0.000858	1	1.377611	up
Com_1786_pos	Tylosin	C46 H77 N O17	915.5111	6.171077	2.625522	0.000864	1	1.322822	up
Com_4038_pos	Cortisol	C21 H30 O5	362.21	2.78758	1.479013	0.000865	0.972222	1.269928	up
Com_452_pos	Palmitoylcarnitine	C23 H45 N O4	399.3352	1.833788	0.874827	0.000916	1	1.303258	up
Com_2000_pos	N-(4-butyl-2-methylphenyl)-N’-[4-(4-methylpiperazino)phenyl]urea	C23 H32 N4 O	380.2543	16.52322	4.046423	0.000922	1	1.293461	up
Com_5322_pos	Oleandomycin	C35 H61 N O12	687.412	6.579337	2.717942	0.000954	0.944444	1.320658	up
Com_4148_pos	4-oxododecanedioic acid	C12 H20 O5	266.1135	2.277737	1.187601	0.000963	0.972222	1.298005	up
Com_11204_pos	Piperine	C17 H19 N O3	570.2803	7.52644	2.911968	0.000988	0.972222	1.261291	up
Com_523_pos	PC (22:6e/18:3)	C48 H80 N O7 P	813.5527	11.53564	3.528026	0.001002	1	1.374866	up
Com_11443_pos	PC (18:4/20:5)	C46 H74 N O8 P	799.5039	8.473563	3.082969	0.001006	0.944444	1.268179	up
Com_2851_pos	PC (14:0/16:3)	C38 H70 N O8 P	699.4828	7.773098	2.95849	0.001028	0.944444	1.339011	up
Com_9318_pos	β-Hydroxythiofentanyl	C20 H26 N2 O2 S	358.1761	4.497475	2.169115	0.001046	1	1.239352	up
Com_1228_pos	PC (21:2/20:3)	C49 H88 N O8 P	849.6109	11.65461	3.542829	0.00106	1	1.34861	up
Com_11663_pos	7-(3,4-dihydroxyphenyl)-5-hydroxy-1-(4-hydroxyphenyl)heptan-3-one	C19 H22 O5	330.1487	8.848982	3.145511	0.001063	0.944444	1.030424	up
Com_15076_pos	Milbemycin A3 oxime	C31 H43 N O7	563.2875	4.526768	2.178481	0.001078	0.972222	1.26398	up
Com_20671_pos	N-benzyl-N-isopropyl-N’-(4-isopropylphenyl)thiourea	C20 H26 N2 S	326.1851	4.30093	2.104649	0.00108	0.972222	1.314826	up
Com_2548_pos	PC (15:1/16:4)	C39 H68 N O8 P	709.4556	7.934176	2.98808	0.001085	0.972222	1.341315	up
Com_5305_pos	4-oxo-4-[(pyridin-4-ylmethyl)amino]but-2-enoic acid	C10 H10 N2 O3	206.0696	6.947965	2.79659	0.00115	1	1.329199	up
Com_11766_pos	Coniferyl alcohol	C10 H12 O3	180.0791	1.824223	0.867282	0.001155	0.944444	1.270686	up
Com_18323_pos	4-(3-Methyl-5-oxo-4,5-dihydro-1H-pyrazol-1-yl)benzenesulfonic acid	C10 H10 N2 O4 S	254.0365	5.39821	2.432481	0.001157	0.944444	1.277896	up
Com_3356_pos	Genistein	C15 H10 O5	270.0534	0.2412	−2.0517	0.001158	0.972222	1.305452	down
Com_19913_pos	Asp-Phe	C13 H16 N2 O5	280.1063	5.15738	2.366638	0.001173	0.972222	1.274672	up
Com_2929_pos	PC (17:0/18:1)	C43 H84 N O8 P	773.5881	0.158879	−2.654	0.001266	0.972222	1.204322	down
Com_1317_pos	Lysops 22:5	C28 H46 N O9 P	571.2898	12.29829	3.620386	0.001288	1	1.368784	up
Com_10368_pos	L-arginine	C6 H14 N4 O2	174.112	6.029883	2.59213	0.001298	0.972222	1.320873	up
Com_430_pos	PC (2:0/16:2)	C26 H48 N O8 P	533.3129	9.489545	3.246339	0.001315	1	1.359369	up
Com_7034_pos	dAMP	C10 H14 N5 O6 P	331.0677	0.114601	−3.12531	0.00132	0.972222	1.27693	down
Com_1359_pos	PC (14:1/16:4)	C38 H66 N O8 P	695.4664	10.58371	3.403773	0.001327	0.944444	1.313766	up
Com_14071_pos	6,7,8-trimethoxy-2H-chromen-2-one	C12 H12 O5	258.0467	0.154632	-2.69309	0.001337	0.972222	1.273024	down
Com_1094_pos	PC (18:0/20:3)	C46 H86 N O8 P	811.5972	8.650716	3.112819	0.001338	1	1.327112	up
Com_661_pos	PE (18:1/20:5)	C43 H74 N O8 P	763.5164	0.026353	−5.24591	0.001355	0.944444	1.281756	down
Com_318_pos	PC (18:2/20:5)	C46 H78 N O8 P	803.5321	10.09577	3.335679	0.001415	0.972222	1.307664	up
Com_6585_pos	PC (10:0/10:0)	C28 H56 N O8 P	565.3749	7.250064	2.857994	0.001415	1	1.317026	up
Com_686_pos	PC (20:2/22:6)	C50 H84 N O8 P	857.5773	10.00412	3.322522	0.001426	1	1.31157	up
Com_11229_pos	PC (21:2/20:5)	C49 H84 N O8 P	845.5941	0.183048	−2.4497	0.001429	1	1.335329	down
Com_10047_pos	5-Phenylvaleric Acid	C11 H14 O2	178.0995	3.973953	1.990575	0.00144	0.972222	1.33992	up
Com_5576_pos	PC (3:0/13:1)	C24 H46 N O8 P	507.2973	2.547896	1.349307	0.001508	1	1.218945	up
Com_17731_pos	PC (12:0/13:0)	C33 H66 N O8 P	635.4546	4.311708	2.10826	0.001517	0.944444	1.227598	up
Com_993_pos	N-Acetylanthranilic acid	C9 H9 N O3	179.0586	12.52853	3.647145	0.001529	0.944444	1.261103	up
Com_7465_pos	PC (20:0/22:5)	C50 H90 N O8 P	863.6271	4.529368	2.17931	0.001551	0.944444	1.30798	up
Com_12748_pos	3-[2-(3-Hydroxyphenyl)ethyl]-5-methoxyphenol	C15 H16 O3	244.11	6.55	2.711495	0.001552	0.972222	1.313298	up
Com_7611_pos	VNH	C15 H24 N6 O5	184.0892	2.588666	1.372209	0.001566	0.972222	1.237295	up
Com_9767_pos	ACar 14:3	C21 H36 N O4	365.2561	2.616601	1.387694	0.001625	0.944444	1.268209	up
Com_3584_pos	4-Methoxybenzaldehyde	C8 H8 O2	136.0527	6.454825	2.690378	0.00163	1	1.25343	up
Com_4248_pos	PC (10:0/13:1)	C31 H60 N O8 P	605.4065	6.920124	2.790798	0.001638	1	1.303295	up
Com_12541_pos	7-Hydroxy-3,4-dihydrocarbostyril	C9 H9 N O2	163.0634	4.097626	2.034788	0.001648	0.972222	1.347814	up
Com_18531_pos	6-fluoro-2-methyl-4-quinolyl 5-methyl-3-phenylisoxazole-4-carboxylate	C21 H15 F N2 O3	362.1125	5.42126	2.438628	0.001659	0.944444	1.357827	up
Com_931_pos	PC (18:3e/20:3)	C46 H82 N O7 P	791.5689	8.018274	3.003292	0.001672	0.972222	1.320741	up
Com_4623_pos	Andrographolide	C20 H30 O5	332.1992	2.508745	1.326966	0.001682	0.972222	1.236385	up
Com_5521_pos	PC (16:3/16:4)	C40 H66 N O8 P	737.4495	5.226921	2.385961	0.0017	0.944444	1.26063	up
Com_1895_pos	Hexanoylcarnitine	C13 H25 N O4	259.1787	2.160039	1.111057	0.001728	0.972222	1.247866	up
Com_878_pos	PC (17:0/18:5)	C43 H76 N O8 P	765.535	12.02928	3.588479	0.001739	1	1.223068	up
Com_9674_pos	Methyl-2-aminobenzoate	C8 H9 N O2	151.0636	3.291576	1.718778	0.001776	0.944444	1.278197	up
Com_1441_pos	Azaspiracid-1	C47 H71 N O12	409.7648	7.778763	2.959541	0.001858	0.916667	1.348633	up
Com_3187_pos	Oleic acid	C18 H34 O2	282.256	1.676202	0.745196	0.001882	0.972222	1.270109	up
Com_5258_pos	5-Hydroxytryptophan	C11 H12 N2 O3	220.0852	9.471437	3.243583	0.001913	0.972222	1.28835	up
Com_9431_pos	PC (9:0/13:1)	C30 H58 N O8 P	591.3908	3.365726	1.750918	0.00192	0.972222	1.207353	up
Com_15514_pos	5,8-dihydroxy-10-methyl-5,8,9,10-tetrahydro-2H-oxecin-2-one	C10 H14 O4	180.0793	1.770352	0.824036	0.00194	0.972222	1.256887	up
Com_10973_pos	Quinoline-4-carboxylic acid	C10 H7 N O2	173.048	3.863237	1.94981	0.002036	0.972222	1.334331	up
Com_1997_pos	PC (20:5/20:5)	C48 H76 N O8 P	825.5167	4.695675	2.231332	0.002054	0.888889	1.316441	up
Com_6753_pos	PC (17:0/18:4)	C43 H78 N O8 P	767.5483	3.465852	1.79321	0.00217	1	1.310333	up
Com_9183_pos	3-(3-morpholinopropyl)-2-(2-pyridinyl)-2,3-dihydro-4(1H)-quinazolinone	C20 H24 N4 O2	352.1873	6.338711	2.66419	0.00226	1	1.334265	up
Com_313_pos	7-Ketocholesterol	C27 H44 O2	400.3344	7.385256	2.884648	0.002291	0.972222	1.282635	up
Com_4507_pos	Thiamine	C12 H16 N4 O S	264.1046	0.399678	−1.32309	0.002293	0.972222	1.153204	down
Com_3924_pos	4-decyl-3-hydroxy-5-oxooxolane-2,3-dicarboxylic acid	C16 H26 O7	352.1505	7.819977	2.967164	0.002301	1	1.311238	up
Com_4606_pos	Acetophenone	C8 H8 O	120.0576	1.604371	0.682008	0.002323	1	1.32722	up
Com_13551_pos	6β-Hydroxy-21-desacetyl deflazacort	C23 H29 N O6	415.1982	6.380265	2.673616	0.002332	0.944444	1.321056	up
Com_6014_pos	Gly-Phe	C11 H14 N2 O3	222.1008	7.877853	2.977802	0.002369	1	1.283178	up
Com_10483_pos	19-Nortestosterone	C18 H26 O2	274.1935	3.479929	1.799058	0.002426	0.972222	1.197304	up
Com_237_pos	PC (14:0e/3:0)	C25 H52 N O7 P	509.3488	1.611375	0.688293	0.002505	1	1.45287	up
Com_71_pos	PC (18:1/20:5)	C46 H80 N O8 P	805.548	7.924626	2.986343	0.002514	0.944444	1.292052	up
Com_5757_pos	6-methyl-5-nitroquinoline	C10 H8 N2 O2	188.059	6.678221	2.739464	0.002543	0.916667	1.272376	up
Com_17207_pos	PC (13:1/14:1)	C35 H66 N O8 P	659.4526	2.794845	1.482768	0.002575	0.944444	1.145879	up
Com_4069_pos	Ecgonine	C9 H15 N O3	185.1056	2.271718	1.183784	0.002618	0.916667	1.27606	up
Com_8228_pos	dihydrotachysterol	C28 H46 O	398.3552	3.704304	1.889202	0.002623	0.944444	1.229274	up
Com_5743_pos	PE (18:2/18:3)	C41 H72 N O8 P	737.5013	0.092333	−3.43701	0.002715	0.972222	1.233948	down
Com_20251_pos	N-gamma-Acetyl-N-2-Formyl-5-Methoxykynurenamine	C13 H16 N2 O4	264.1117	2.144745	1.100806	0.002832	0.972222	1.207011	up
Com_3732_pos	3-Methoxy prostaglandin F1α	C21 H38 O6	408.2494	5.905411	2.562037	0.002856	0.916667	1.331931	up
Com_7527_pos	PC (16:1/18:5)	C42 H72 N O8 P	749.4859	6.480887	2.696191	0.002901	0.944444	1.211454	up
Com_10693_pos	PC (2:0/16:3)	C26 H46 N O8 P	531.2974	9.069612	3.181041	0.003062	0.944444	1.196685	up
Com_2749_pos	PC (20:4/20:5)	C48 H78 N O8 P	827.5344	5.666707	2.502511	0.003149	1	1.291505	up
Com_7657_pos	Tetranor-12(S)-HETE	C16 H26 O3	288.1704	2.020589	1.014776	0.003169	0.944444	1.090302	up
Com_2048_pos	Glutamic acid	C5 H9 N O4	147.0533	1.724365	0.786065	0.003183	0.916667	1.177868	up
Com_818_pos	delta-Tocopherol	C27 H46 O2	402.3501	6.30511	2.656522	0.0032	0.972222	1.267494	up
Com_13919_pos	PC (16:2e/18:5)	C42 H72 N O7 P	733.4911	3.976066	1.991342	0.003223	0.888889	1.252882	up
Com_18996_pos	3,5-Diiodo-L-tyrosine	C9 H9 I2 N O3	432.8685	5.117798	2.355523	0.003244	0.944444	1.204947	up
Com_2722_pos	PC (16:1e/3:0)	C27 H54 N O7 P	535.3643	1.690855	0.757753	0.00327	0.944444	1.059929	up
Com_2780_pos	2-methyl-2,3,4,5-tetrahydro-1,5-benzoxazepin-4-one	C10 H11 N O2	159.0688	0.011727	−6.41403	0.003314	0.972222	1.313456	down
Com_11179_pos	PC (18:5e/20:5)	C46 H74 N O7 P	783.5135	0.11267	−3.14983	0.003332	1	1.306937	down
Com_10637_pos	2-[(3S)-1-(3,4-Difluorobenzyl)-3-pyrrolidinyl]-1,3-benzoxazole	C18 H16 F2 N2 O	314.1271	2.782689	1.476479	0.003349	1	1.221312	up
Com_14584_pos	2-[5-(2-hydroxypropyl)oxolan-2-yl]propanoic acid	C10 H18 O4	224.1047	4.706909	2.23478	0.003356	0.944444	1.293078	up
Com_10663_pos	8,8-dimethyl-2H,8H-pyrano[3,2-g]chromen-2-one	C14 H12 O3	228.0767	5.854847	2.549632	0.003381	0.916667	1.272253	up
Com_668_pos	PC (20:5/22:6)	C50 H78 N O8 P	851.5312	7.58277	2.922725	0.003403	0.944444	1.250953	up
Com_759_pos	PC (20:3/22:6)	C50 H82 N O8 P	855.5633	7.331326	2.874074	0.003453	0.972222	1.28679	up
Com_679_pos	Cholecalciferol	C27 H44 O	384.3395	5.613831	2.488986	0.003612	0.916667	1.252435	up
Com_7178_pos	PC (18:3/18:4)	C44 H74 N O8 P	793.5223	3.389316	1.760994	0.003631	1	1.268067	up
Com_4601_pos	PC (20:0/22:6)	C50 H88 N O8 P	1723.222	4.333388	2.115495	0.003658	0.944444	1.242839	up
Com_17419_pos	Bialaphos	C11 H22 N3 O6 P	305.1173	4.121276	2.043091	0.003671	0.888889	1.110434	up
Com_11117_pos	(2E)-6-hydroxy-2-methyl-6-(4-methylphenyl)hept-2-enoic acid	C15 H20 O3	248.1418	2.185587	1.128021	0.003672	0.972222	1.254908	up
Com_2043_pos	PC (22:6e/7:0)	C37 H64 N O7 P	665.4321	7.881947	2.978552	0.003735	0.972222	1.270375	up
Com_8339_pos	2,3,4,9-Tetrahydro-1H-β-carboline-3-carboxylic acid	C12 H12 N2 O2	216.0905	0.473158	−1.07961	0.003769	0.944444	1.152213	down
Com_20968_pos	3-(3,4-dimethylphenyl)-3,4-dihydro-1,2,3-benzotriazin-4-one	C15 H13 N3 O	268.1309	1.914408	0.936898	0.003793	0.944444	1.177922	up
Com_13321_pos	Dehydrocholic acid	C24 H34 O5	402.2408	2.217446	1.148899	0.003823	0.916667	1.223449	up
Com_17038_pos	1,6-dihydroxy-3-methoxy-8-methyl-9H-xanthen-9-one	C15 H12 O5	272.0691	0.526498	−0.9255	0.003962	0.972222	1.54698	down
Com_18457_pos	α-Lapachone	C15 H14 O3	264.0752	5.210088	2.381308	0.003986	0.916667	1.222303	up
Com_21270_pos	Dihydrokawain	C14 H16 O3	232.1088	2.160902	1.111634	0.00407	0.888889	1.265708	up
Com_12429_pos	PC (7:0/8:0)	C23 H46 N O8 P	473.3233	1.992399	0.994507	0.004123	0.944444	1.336774	up
Com_5266_pos	fentanyl-d5	C22 H23 [2]H5 N2 O	341.2564	2.693412	1.429435	0.004153	1	1.208121	up
Com_387_pos	PC (20:3/20:3)	C48 H84 N O8 P	833.5793	5.510795	2.462261	0.004217	0.944444	1.260406	up
Com_19962_pos	Citrinin	C13 H14 O5	232.0719	3.59944	1.847772	0.004226	0.916667	1.286702	up
Com_7534_pos	α-Zearalanol	C18 H26 O5	344.1601	3.432745	1.779363	0.004261	0.916667	1.212612	up
Com_5080_pos	5-Methoxyindole-3-Carbaldehyde	C10 H9 N O2	175.0638	0.10386	−3.26729	0.004293	0.944444	1.268492	down
Com_307_pos	1H-indene-3-carboxamide	C10 H9 N O	159.0688	0.010549	−6.56673	0.004301	0.916667	1.279381	down
Com_22293_pos	Orsellinic acid ethyl ester	C10 H12 O4	196.0741	8.681433	3.117933	0.004328	0.944444	1.208947	up
Com_9878_pos	Palmitoylethanolamide	C18 H37 N O2	299.2825	1.559393	0.640985	0.004344	0.944444	1.330338	up
Com_9506_pos	5-Hydroxyindole-2-carboxylic acid	C9 H7 N O3	177.0427	6.695613	2.743216	0.004491	0.944444	1.212783	up
Com_8679_pos	10-Nitrolinoleate	C18 H31 N O4	307.2148	7.343549	2.876478	0.004507	0.972222	1.185788	up
Com_7141_pos	PC (22:4/22:5)	C52 H86 N O8 P	883.594	3.436426	1.780909	0.004595	0.888889	1.209455	up
Com_7411_pos	4-methoxy-6-(prop-2-en-1-yl)-2H-1,3-benzodioxole	C11 H12 O3	210.0872	3.64472	1.865808	0.004734	0.861111	1.279364	up
Com_4863_pos	PC (22:6e/15:1)	C45 H78 N O7 P	775.5365	4.434001	2.148609	0.004755	0.944444	1.159716	up
Com_1379_pos	Serotonin	C10 H12 N2 O	176.0953	0.010909	−6.51839	0.004944	0.888889	1.267514	down
Com_12517_pos	PC (18:0e/10:0)	C36 H74 N O7 P	646.4799	3.083211	1.624434	0.004989	0.972222	1.196691	up
Com_8664_pos	PC (22:5e/18:3)	C48 H82 N O7 P	815.5852	0.165497	−2.59513	0.005075	0.916667	1.090942	down
Com_2724_pos	Oleoyl ethanolamide	C20 H39 N O2	307.2878	2.124355	1.087025	0.005129	0.888889	1.204993	up
Com_6202_pos	1-[4-hydroxy-3-(3-methylbut-2-en-1-yl)phenyl]ethan-1-one	C13 H16 O2	204.1154	4.144982	2.051366	0.005132	0.888889	1.245071	up
Com_3407_pos	Carvone	C10 H14 O	150.1047	3.029551	1.599104	0.005198	0.888889	1.250376	up
Com_5828_pos	N1,N2-dicyclohexylethanedithioamide	C14 H24 N2 S2	284.1392	4.579196	2.195094	0.005272	0.916667	1.211969	up
Com_7360_pos	(2S)-2-(2-hydroxypropan-2-yl)-2H,3H,7H-furo[3,2-g]chromen-7-one	C14 H14 O4	246.0873	4.465566	2.158843	0.00535	0.861111	1.243047	up
Com_8812_pos	ACar 15:0	C22 H44 N O4	385.3195	2.097002	1.068329	0.005353	1	1.244115	up
Com_19599_pos	gamma-Tocopherol	C28 H48 O2	416.3659	1.729413	0.790282	0.005484	0.916667	1.116559	up
Com_1363_pos	Uric acid	C5 H4 N4 O3	168.0286	0.023454	−5.41399	0.005496	0.833333	1.256219	down
Com_1255_pos	PC (22:5e/16:2)	C46 H80 N O7 P	789.5541	7.652586	2.935947	0.005522	0.944444	1.152567	up
Com_4419_pos	5-(6-hydroxy-6-methyloctyl)-2,5-dihydrofuran-2-one	C13 H22 O3	208.1468	3.344166	1.741646	0.005543	0.861111	1.232657	up
Com_13875_pos	4-Methoxycinnamic Acid	C10 H10 O3	178.0637	1.844046	0.882874	0.005558	0.972222	1.187844	up
Com_19209_pos	D-Glucosamine 6-phosphate	C6 H14 N O8 P	259.0464	3.991602	1.996968	0.005651	0.888889	1.256275	up
Com_922_pos	ACar 16:1	C23 H44 N O4	397.3198	2.321532	1.215077	0.005665	1	1.216084	up
Com_3969_pos	N-Formylkynurenine	C11 H12 N2 O4	236.0801	6.833696	2.772666	0.005892	0.972222	1.227286	up
Com_5014_pos	4-(anilinomethylidene)-3-methyl-4,5-dihydroisoxazol-5-one	C11 H10 N2 O2	220.0852	4.480045	2.163513	0.005913	0.916667	1.080931	up
Com_18005_pos	Pyridoxine O-Glucoside	C14 H21 N O8	331.1278	1.539478	0.622441	0.005954	0.944444	1.102022	up
Com_5671_pos	(±)-Abscisic acid	C15 H20 O4	286.1186	4.093891	2.033473	0.005962	0.833333	1.223662	up
Com_5948_pos	Emamectin B1a	C49 H75 N O13	885.5377	4.040586	2.014565	0.005975	0.916667	1.208262	up
Com_19924_pos	5-Methyltetrahydrofolic acid	C20 H25 N7 O6	459.1879	0.063769	−3.971	0.006189	0.833333	1.249912	down
Com_2588_pos	LPE 18:3	C23 H42 N O7 P	475.2706	5.712052	2.514009	0.006298	0.916667	1.195536	up
Com_13256_pos	Lysope 14:0	C19 H40 N O7 P	425.2555	2.580516	1.367659	0.006334	0.888889	1.097721	up
Com_14364_pos	Tetrahydrocortisone	C21 H32 O5	381.2521	1.843351	0.882331	0.006336	0.916667	1.25769	up
Com_5864_pos	Gly-Tyr	C11 H14 N2 O4	238.0957	4.048931	2.017541	0.00673	0.861111	1.092165	up
Com_3254_pos	indoline-2-carboxylic acid	C9 H9 N O2	185.0457	4.775598	2.255681	0.006786	0.888889	1.209541	up
Com_10653_pos	PC (2:0/3:0)	C13 H26 N O8 P	355.1398	4.446334	2.152616	0.006832	0.944444	1.164047	up
Com_21614_pos	N-{4-[(2R,3R)-3-(Hydroxymethyl)-5-oxo-2-morpholinyl]phenyl}acetamide	C13 H16 N2 O4	286.0959	6.806228	2.766855	0.006875	0.888889	1.223151	up
Com_10347_pos	Levothyroxine	C15 H11 I4 N O4	776.6887	0.079678	−3.64967	0.006911	0.861111	1.234821	down
Com_8576_pos	7,8-Dihydrobiopterin	C9 H13 N5 O3	239.1023	0.192781	−2.37497	0.007025	0.888889	1.186453	down
Com_13967_pos	3,5-dimethyl-1-phenyl-1,5-dihydro-4H-pyrazolo[4,3-c]quinolin-4-one	C18 H15 N3 O	289.1222	3.564029	1.833509	0.007196	0.861111	1.108788	up
Com_8637_pos	PC (19:2/22:6)	C49 H82 N O8 P	843.5629	3.652079	1.868718	0.00721	0.916667	1.1036	up
Com_1190_pos	Epigallocatechin	C15 H14 O7	612.1523	2.722847	1.445116	0.007214	0.944444	1.17428	up
Com_6899_pos	Vitamin A	C20 H30 O	286.2296	1.734526	0.794542	0.007244	1	1.367315	up
Com_8375_pos	4,4’-Bis(3-methyl-5-oxo-1-phenylpyrazolinylidene)	C20 H16 N4 O2	163.5508	6.771345	2.759442	0.007283	0.916667	1.009452	up
Com_14579_pos	ACar 12:3	C19 H32 N O4	337.2257	1.941823	0.957412	0.00752	0.888889	1.198904	up
Com_435_pos	ACar 18:1	C25 H48 N O4	425.351	1.831328	0.87289	0.007531	1	1.181896	up
Com_11898_pos	5,6-dimethyl-4-oxo-4H-pyran-2-carboxylic acid	C8 H8 O4	168.0429	2.295537	1.198832	0.007562	0.916667	1.236089	up
Com_17650_pos	4-(4-nitrophenylazo)aniline	C12 H10 N4 O2	264.0575	0.098735	−3.34029	0.007695	0.833333	1.260904	down
Com_3643_pos	5α-Dihydrotestosterone	C19 H30 O2	290.2248	4.480342	2.163609	0.007734	0.916667	1.122599	up
Com_815_pos	Biliverdin	C33 H34 N4 O6	582.249	0.012681	−6.30119	0.007799	0.833333	1.222679	down
Com_478_pos	octadec-9-ynoic acid	C18 H32 O2	262.2297	2.902232	1.537163	0.007962	0.944444	1.160734	up
Com_2081_pos	Prostaglandin E2-1-glyceryl ester	C23 H38 O7	408.2492	3.718726	1.894808	0.008077	0.861111	1.211999	up
Com_7960_pos	tetranor-12(R)-HETE	C16 H26 O3	248.1779	1.775687	0.828378	0.008235	0.916667	1.098215	up
Com_10827_pos	2-phenyl[1,3]oxazolo[4,5-c]quinolin-4(5H)-one	C16 H10 N2 O2	262.0745	2.846852	1.509367	0.008632	0.916667	1.125244	up
Com_14179_pos	6-Hydroxymelatonin	C13 H16 N2 O3	248.1166	6.197052	2.631582	0.00865	0.972222	1.208966	up
Com_4244_pos	(S)-Equol	C15 H14 O3	242.0949	0.559466	−0.83788	0.008786	0.916667	1.153431	down
Com_3673_pos	10-Undecenoic acid	C11 H20 O2	184.1466	2.701396	1.433705	0.009025	0.833333	1.221054	up
Com_10879_pos	PC (22:4e/18:3)	C48 H84 N O7 P	817.5931	0.153883	−2.7001	0.009073	0.833333	1.224782	down
Com_2765_pos	9,10-Dihome	C18 H34 O4	314.2462	8.610415	3.106083	0.009544	0.972222	1.226366	up
Com_15554_pos	cis-gondoic acid	C20 H38 O2	310.2868	1.526959	0.610661	0.009884	0.916667	1.145105	up
Com_8358_pos	Artemisinin	C15 H22 O5	264.1367	2.766354	1.467986	0.009904	0.833333	1.146814	up
Com_10522_pos	PC (22:6e/2:0)	C32 H54 N O7 P	595.3636	0.154516	−2.69417	0.010051	0.861111	1.188352	down
Com_15858_pos	NSI-189	C22 H30 N4 O	366.2405	2.636949	1.398869	0.010315	0.944444	1.143415	up
Com_3015_pos	LPE 20:3	C25 H46 N O7 P	503.3015	0.342076	−1.54761	0.010341	0.888889	1.159162	down
Com_8047_pos	L-Adrenaline	C9 H13 N O3	183.0901	2.569493	1.361484	0.010742	0.888889	1.119331	up
Com_2278_pos	Indole-3-acetic acid	C10 H9 N O2	175.0637	0.510014	-0.97139	0.011144	0.916667	1.071783	down
Com_1224_pos	PC (20:2/20:3)	C48 H86 N O8 P	835.61	0.286329	−1.80425	0.011149	0.916667	1.112693	down
Com_6968_pos	PE (18:0/20:5)	C43 H76 N O8 P	765.5333	0.223	−2.16488	0.011513	0.916667	1.187518	down
Com_15873_pos	Phe-Phe	C18 H20 N2 O3	312.1479	0.482743	−1.05067	0.011611	0.916667	1.096299	down
Com_6778_pos	4-methyl-5-oxo-2-pentyl-2,5-dihydrofuran-3-carboxylic acid	C11 H16 O4	212.1027	1.956129	0.968001	0.011784	0.861111	1.157078	up
Com_13949_pos	N-Oleoyl Glycine	C20 H37 N O3	339.277	4.199308	2.070152	0.012182	0.888889	1.093162	up
Com_6369_pos	PC (16:2e/20:4)	C44 H78 N O7 P	763.5522	0.434942	−1.20111	0.012339	0.944444	1.07808	down
Com_1948_pos	PC (22:6e/19:2)	C49 H84 N O7 P	829.5849	4.052929	2.018965	0.012393	0.916667	1.135992	up
Com_15928_pos	PC (15:1/15:1)	C38 H72 N O8 P	701.4998	2.01445	1.010386	0.01241	0.888889	1.131789	up
Com_4607_pos	11β-Prostaglandin F2α	C20 H34 O5	376.2232	3.43434	1.780033	0.01244	1	1.125194	up
Com_14083_pos	5-Hydroxylysine	C6 H15 Cl N2 O3	198.0774	0.192787	−2.37492	0.013019	0.833333	1.178386	down
Com_9508_pos	AL 8810 Methyl ester	C25 H33 F O4	438.2245	2.754685	1.461887	0.013021	0.944444	1.095307	up
Com_138_pos	PC (20:1/18:2)	C46 H86 N O8 P	811.6102	0.503018	−0.99132	0.013453	0.916667	1.057681	down
Com_19749_pos	Thromboxane B1	C20 H36 O6	394.2344	2.305326	1.204971	0.013491	0.888889	1.113985	up
Com_9027_pos	16,16-Dimethyl prostaglandin A2	C22 H34 O4	384.2279	2.318968	1.213483	0.013916	0.916667	1.100742	up
Com_3836_pos	PC (21:2/20:4)	C49 H86 N O8 P	847.6103	0.137758	−2.85979	0.013995	0.972222	1.09977	down
Com_1928_pos	PC (22:6e/18:1)	C48 H84 N O7 P	817.6004	0.021271	−5.555	0.014385	0.944444	1.169896	down
Com_6097_pos	Pro-Leu	C11 H20 N2 O3	228.1481	1.744169	0.80254	0.014481	0.916667	1.139845	up
Com_12690_pos	Propionyl-L-carnitine	C10 H19 N O4	217.1321	1.502086	0.586967	0.014902	0.888889	1.053018	up
Com_5650_pos	Neosaxitoxin	C10 H17 N7 O5	315.1324	10.97464	3.456101	0.015193	1	1.120191	up
Com_14931_pos	3-hydroxy-2-octylpentanedioic acid	C13 H24 O5	242.152	4.299715	2.104241	0.015841	0.944444	1.095875	up
Com_3998_pos	Genistein 4’-O-glucuronide	C21 H18 O11	446.0859	0.465087	−1.10443	0.015924	0.888889	1.130713	down
Com_3341_pos	Indole-3-pyruvic acid	C11 H9 N O3	203.0588	0.350851	−1.51107	0.016203	0.888889	1.083663	down
Com_5731_pos	trans-3-Indoleacrylic acid	C11 H9 N O2	187.0636	4.14403	2.051034	0.016397	1	1.148513	up
Com_11499_pos	3-Methylindole	C9 H9 N	131.0738	0.10326	−3.27565	0.017201	0.833333	1.135941	down
Com_22041_pos	SM (d14:2/27:0)	C46 H91 N2 O6 P	798.6748	0.566551	−0.81972	0.017801	1	1.035494	down
Com_3183_pos	LPE 22:5	C27 H46 N O7 P	527.3013	0.366177	−1.44939	0.018047	0.861111	1.054385	down
Com_3983_pos	Prolylleucine	C11 H20 N2 O3	228.1477	1.662171	0.733069	0.018336	0.888889	1.164819	up
Com_4421_pos	ACar 18:3	C25 H44 N O4	421.3198	1.740549	0.799542	0.019957	0.888889	1.071188	up
Com_16400_pos	N-Arachidonoyl-L-serine	C23 H37 N O4	391.2715	2.34938	1.23228	0.02031	0.944444	1.113185	up
Com_13446_pos	N-lactoyl-phenylalanine	C12 H15 N O4	237.1002	3.002393	1.586113	0.020833	0.888889	1.016525	up
Com_17463_pos	PC (20:1/22:6)	C50 H86 N O8 P	859.6093	0.329233	−1.60282	0.021175	1	1.119401	down
Com_15043_pos	2-piperidino-6-(2-thienyl)-4-(trifluoromethyl)nicotinonitrile	C16 H14 F3 N3 S	337.0891	5.662053	2.501325	0.021192	0.888889	1.10242	up
Com_14240_pos	PC (20:4e/4:0)	C32 H58 N O7 P	599.3964	0.501615	−0.99535	0.022678	0.861111	1.039006	down
Com_17320_pos	PC (3:0/16:2)	C27 H50 N O8 P	547.3312	0.2646	−1.91811	0.02277	0.833333	1.119663	down
Com_9970_pos	PC (17:1/17:2)	C42 H78 N O8 P	755.5443	0.515006	−0.95734	0.022982	0.861111	1.145029	down
Com_5024_pos	LPE 20:5	C25 H42 N O7 P	499.2709	5.165532	2.368917	0.023474	0.833333	1.018851	up
Com_1632_pos	Xanthurenic acid	C10 H7 N O4	205.0381	0.230299	−2.11842	0.023832	0.916667	1.049548	down
Com_11917_pos	PC (18:0/18:0)	C44 H88 N O8 P	789.6222	0.314925	−1.66692	0.024144	0.833333	1.090918	down
Com_802_pos	N-Tetradecanamide	C14 H29 N O	227.2251	0.591712	−0.75703	0.025274	0.833333	1.05689	down
Com_10403_pos	Bilirubin	C33 H36 N4 O6	584.2652	0.109041	−3.19706	0.025419	0.861111	1.062179	down
Com_59_pos	Stearamide	C18 H37 N O	283.2877	0.538366	−0.89334	0.025441	0.777778	1.063391	down
Com_5278_pos	ACar 20:1	C27 H52 N O4	453.3824	1.554363	0.636323	0.02595	0.861111	1.094664	up
Com_10062_pos	PC (20:4/20:4)	C48 H80 N O8 P	829.5574	0.116679	−3.09938	0.026956	0.861111	1.090287	down
Com_20712_pos	PC (19:0/19:0)	C46 H92 N O8 P	817.648	0.54013	−0.88862	0.026999	0.833333	1.042002	down
Com_3553_pos	5-Hydroxyindole	C8 H7 N O	133.0531	0.460354	−1.11918	0.027623	0.861111	1.066928	down
Com_12843_pos	PC (17:2/22:6)	C47 H78 N O8 P	1631.092	0.24598	−2.02339	0.029442	0.833333	1.085861	down
Com_3102_pos	Spermidine	C7 H19 N3	128.1316	0.413641	−1.27355	0.030146	0.833333	1.125967	down
Com_149_pos	LPE 22:6	C27 H44 N O7 P	525.2859	0.382305	−1.3872	0.03179	0.861111	1.003913	down
Com_7377_pos	GNH	C12 H18 N6 O5	326.1349	0.376668	−1.40863	0.032065	0.861111	1.017779	down
Com_7125_pos	PC (22:4e/16:3)	C46 H80 N O7 P	789.5582	0.288885	−1.79143	0.033127	0.833333	1.095894	down
Com_5071_pos	PC (19:2/20:5)	C47 H80 N O8 P	817.5618	0.050336	−4.31227	0.034415	0.694444	1.057781	down
Com_9034_pos	PC (17:2/18:5)	C43 H72 N O8 P	761.5017	0.106557	−3.2303	0.035737	0.694444	1.040771	down
Com_2180_pos	LSD-d3	C20 H22 [2]H3 N3 O	326.2224	1.538971	0.621966	0.036471	0.888889	1.225425	up
Com_8715_pos	LPE 20:1	C25 H50 N O7 P	507.3339	0.346915	−1.52734	0.038209	0.861111	1.026889	down
Com_11013_pos	PC (14:0e/17:2)	C39 H76 N O7 P	701.5344	0.167245	−2.57996	0.041215	0.805556	1.062801	down
Com_7836_pos	PC (22:6/22:6)	C52 H80 N O8 P	877.5625	0.139886	−2.83767	0.041247	0.694444	1.016207	down
Com_9497_pos	PC (18:3/20:5)	C46 H76 N O8 P	801.5277	0.050978	−4.29397	0.041334	0.694444	1.036023	down
Com_32_pos	Oleoyl ethylamide	C20 H39 N O	309.3033	0.586412	−0.77001	0.041805	0.777778	1.013935	down
Com_3656_pos	(5Z)-3-aminonon-5-enoic acid	C9 H17 N O2	171.1262	1.758567	0.8144	0.042484	0.833333	1.119663	up

### Metformin alleviates X/XO-induced osteoblast damage

**Fig. 3:**
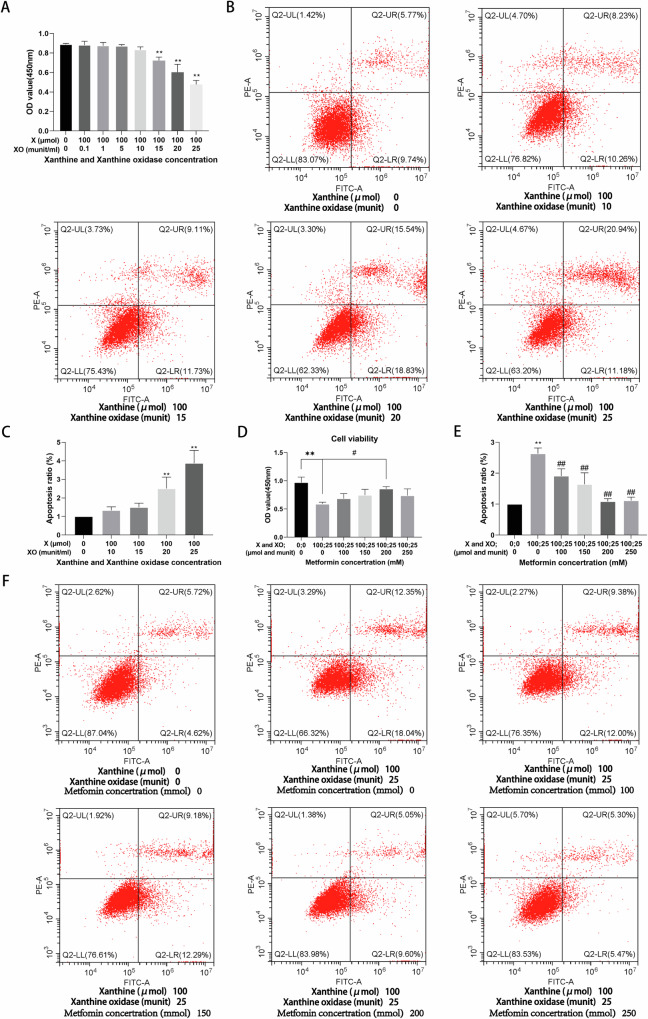
Metformin alleviates X/XO-induced osteoblast damage. **A** Cell viability with different xanthine oxidase concentrations treatment. **B** Flow cytometry cell apoptosis assay under different xanthine oxidase concentrations. **C** Statistical results of cell apoptosis rate in (**B**). **D** Cell viability under different metformin concentrations. **E** Statistical results of cell apoptosis rate in (**F**). **F** Flow cytometry cell apoptosis assay under different metformin concentrations. Experiments were implemented in triplicate. Data are means ± SDs, *p < 0.05, **p < 0.01 compared with control cells and #*p* < 0.05, ##*p* < 0.01 compared with X/XO applied analysed by using ANOVA.

Xanthine and xanthine oxidase were used to construct a model of disordered purine metabolism at the cellular level. First, we aimed to determine the optimal concentration of xanthine oxidase with an adequate xanthine concentration (100 µmol) for decomposition through CCK-8 and flow cytometry assays (Fig. 3A, B). The results revealed that 25 mU/ml xanthine oxidase significantly reduced viability while increasing the percentage of apoptotic osteoblasts (Fig. 3A, C). Therefore, 25 mU/ml xanthine oxidase was used for subsequent experiments. We subsequently explored the role of metformin in the treatment of postmenopausal osteoporosis. Our experiments revealed that 200 mmol of metformin significantly reversed the decreases in cell viability and the percentage of apoptotic cells caused by X/XO treatment (Fig. 3D–F). Therefore, 200 mmol of metformin was used in subsequent experiments.

### Metformin increases HPRT1 expression to reverse oxidative damage in osteoblasts

**Fig. 4:**
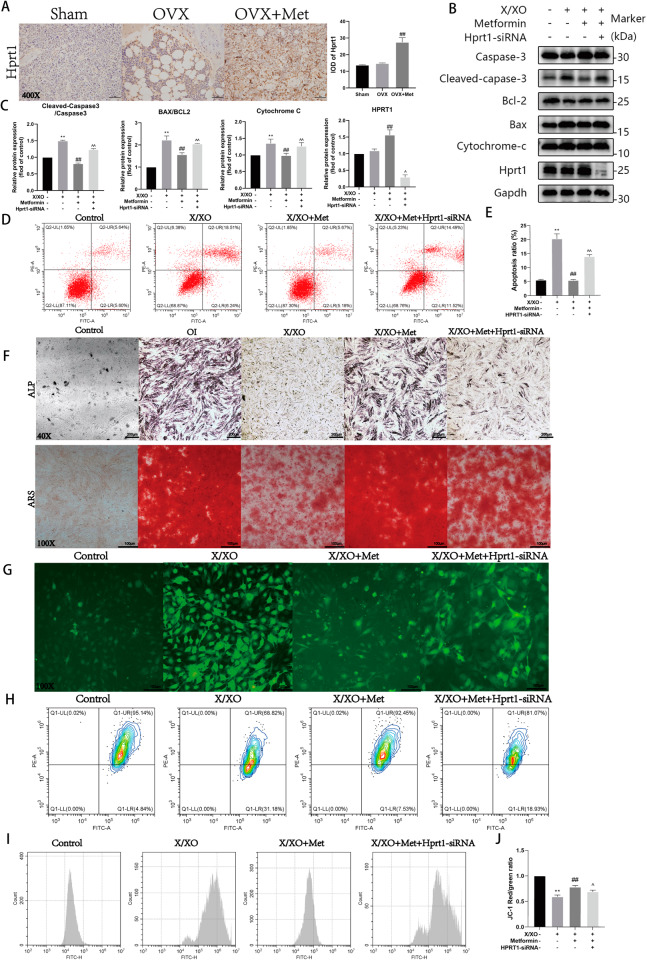
Metformin increases HPRT1 expression to reverse oxidative damage of osteoblasts. **A** Immunohistochemistry and IOD (integrated optical density) of bone tissue after staining HPRT1 antibody. **B** Detection of mitochondrial apoptosis related proteins by Western blotting. **C** Relative protein expression levels of the proteins in (**B**). **D** Flow cytometry to detect cell apoptosis with metformin and HPRT1-siRNA. **E** Statistical results of cell apoptosis rate in (**D**). **F** Differentiation levels of osteoblasts detected by ALP and alizarin red staining. **G** ROS levels detected by a fluorescent probe with X/XO and metformin treatment. **H** Mitochondrial membrane potential levels after above treatment. **I** Quantitative changes in mitochondrial membrane potential detected by using a full-wavelength multifunctional microplate reader in (**H**). **J** Flow cytometry detection of intracellular calcium ion staining related wavelengths Experiments were implemented in triplicate. Data are means ± SDs, *p < 0.05, **p < 0.01 compared with control cells, #*p* < 0.05, ##*p* < 0.01 compared with X/XO and ^*p* < 0.05, ^^*p* < 0.01 compared with X/XO+Met applied analysed by using ANOVA.

The results of network pharmacology analysis revealed that HPRT1 was the preferable target because of its important effect on the salvage pathway of purine generation. We performed immunohistochemical analysis of femoral tissue samples to confirm the changes in HPRT1 expression in vivo (Fig. 4A). No significant changes were observed in the number of HPRT1-positive puncta between the OVX group and the sham group, but the number of HPRT1-positive puncta was greater after metformin treatment. Consistent with the animal results, the expression of HPRT1 increased with metformin intervention in vitro (Fig. 4B, C). We also generated HPRT1-silenced cells for further verification (Fig. S1E). The percentage of apoptotic cells increased after the inhibition of HPRT1 expression (Fig. 4D, E). We further examined the relevant apoptotic protein levels via western blotting. The relative expression ratios of cleaved caspase-3/caspase-3 and BAX/BCL-2 were significantly greater with X/XO treatment. Metformin increased the proportion of related proteins, which was reversed after HPRT1 silencing (Fig. 4B, C). Additionally, we studied the ability of MC3T3-E1 cells to differentiate through ALP and alizarin red staining. As shown in Fig. 4F, metformin restored the differentiation and mineralization ability of osteoblasts after X/XO treatment. Silencing HPRT1 expression reversed the therapeutic effects of metformin. Then, we performed JC-1 and DCFH-DA staining to detect the mitochondrial membrane potential and oxidative state of the osteoblasts. The results indicated that the mitochondrial membrane potential decreased and the ROS levels increased with X/XO treatment and that the therapeutic effect of metformin was reversed by silencing HPRT1 (Fig. 4G, H, I). The intracellular calcium ion concentration also indicated the degree of oxidative damage (Fig. 4J). These results indicated that metformin upregulated the expression of HPRT1 to ameliorate the oxidative stress-induced damage induced by X/XO in osteoblasts.

### SIRT3-mediated deacetylation of FoxO1 promotes its nuclear localization to regulate HPRT1 expression

**Fig. 5:**
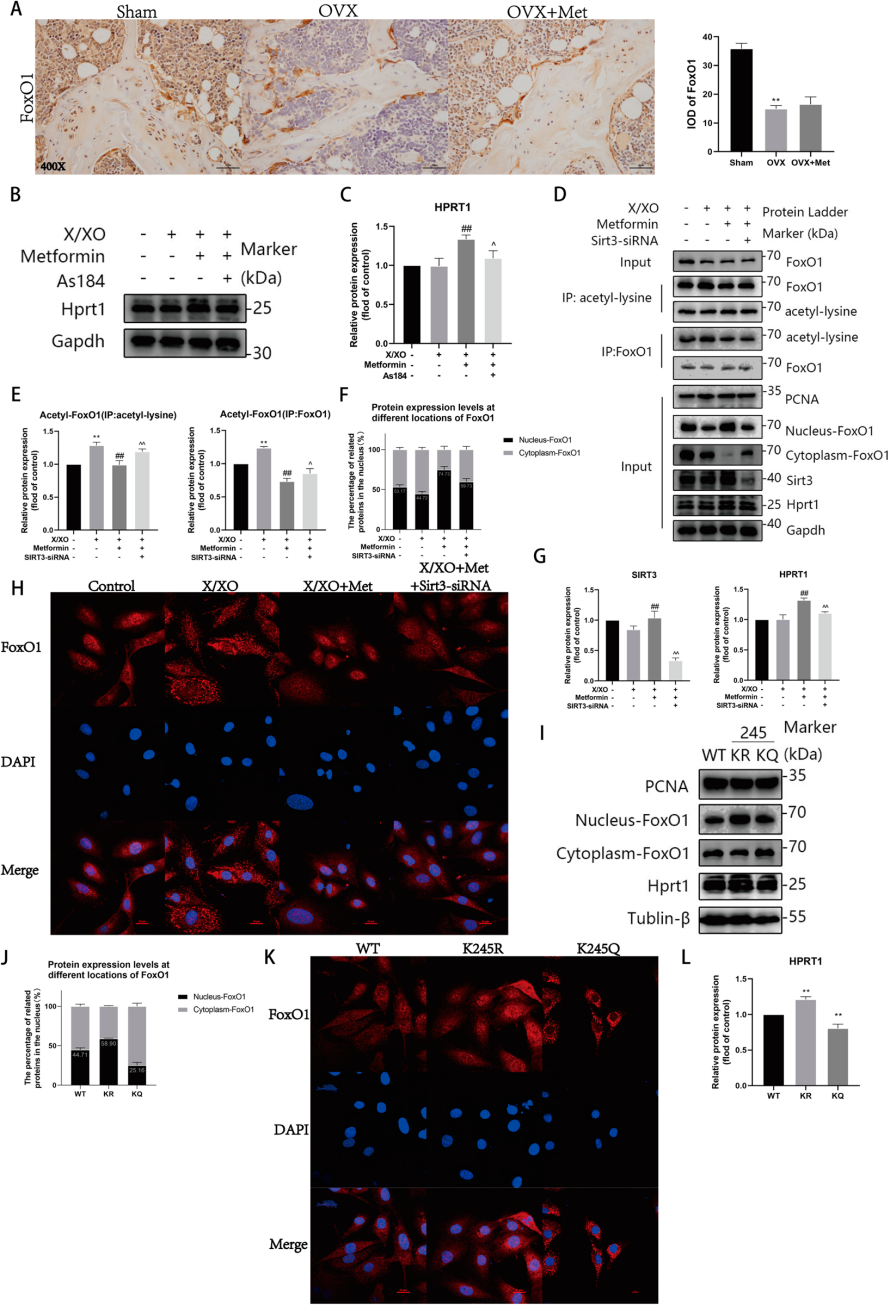
SIRT3-mediated deacetylation of FoxO1 promotes its nuclear localization to regulate HPRT1 expression. **A** Immunohistochemistry and IOD (integrated optical density) of bone tissue after staining FoxO1 antibody. **B** Detection of HPRT1 protein by Western blotting with As184 addition. **C** Relative protein expression levels of the proteins in (**B**). **D** Detection of related proteins by Western blotting with metformin and Sirt3-siRNA treatment. **E**, **F** Protein of FoxO1 subcellular localization level in (**D**). **G** SIRT3 and HPRT1 protein expression levels of the proteins in (**D**). **H** Confocal microscopy to detect subcellular localization of FoxO1 proteins in different groups. **I** Detection of FoxO1 nucleo-cytoplasmic ratio and HPRI1 expression by Western blotting after acetylation site mutation. **J** Protein of FoxO1 subcellular localization level in (**F**). **K** Confocal microscopy to detect subcellular localization of FoxO1 proteins in different groups. **L** HPRT1 protein expression levels of the proteins in (**F**). Experiments were implemented in triplicate. Data are means ± SDs, *p < 0.05, **p < 0.01 compared with control cells or WT, #*p* < 0.05, ##*p* < 0.01 compared with X/XO and ^*p* < 0.05, ^^*p* < 0.01 compared with X/XO+Met applied analysed by using ANOVA.

To investigate the enrichment of the FoxO signalling pathway, we detected differences in FoxO1 protein expression in bone tissues. The FoxO1 levels in the OVX and metformin treatment groups were lower than those in the sham group (Fig. 5A). At the cellular level, we added As184 to inhibit FoxO1 expression, and the HPRT1 protein level decreased (Fig. 5B, C). The localization of FoxO1 determines its function, and its acetylation level regulates its subcellular localization. SIRT3 is a deacetylase that was shown to improve the oxidative stress state in our previous study. We first detected the subcellular localization of FoxO1 at different acetylation levels via western blotting. Compared with that in the control group, the acetylation level of FoxO1 in the model group increased, and its nuclear localization decreased. In the metformin treatment group, SIRT3 levels increased, FoxO1 acetylation levels decreased, and FoxO1 nuclear localization increased. Sirt3 gene silencing reversed these effects and decreased the levels of the corresponding HPRT1 protein (Fig. 5D–G). At the same time, we used confocal microscopy to detect the nuclear and cytoplasmic fluorescence localization of the FoxO1 protein after different treatments. Compared with that in the control group, FoxO1 in purine-damaged cells accumulated mainly in the cytoplasm. Metformin treatment increased the proportion of FoxO1 protein localized to the nucleus, but Sirt3 silencing reversed this effect (Fig. 5H). These results indicate that metformin can regulate the acetylation of FoxO1 through SIRT3 to promote its nuclear localization and ultimately regulate HPRT1 expression. Additionally, we aimed to identify the acetylation sites in FoxO1. We used software to predict the acetylation sites of FoxO1 regulated by SIRT3. We constructed a point mutation plasmid containing acetylation site 245 and transfected it into MC3T3-E1 cells. Compared with that in wild-type cells, the nuclear localization of FoxO1 was increased in K245R-transfected cells, and correspondingly, the HPRT1 protein level was increased (Fig. 5I, J, L). The results for K245Q-transfected cells were the opposite. Accordingly, we used confocal microscopy to detect the visualization and localization of FoxO1 protein after point mutation. The K245R group showed more FoxO1 protein nuclear localization, while the K245Q group showed the opposite (Fig. 5K). These findings indicate that the regulation of FoxO1 by SIRT3 is achieved through acetylation at site -245.

### The transcriptome data suggest that autophagy is involved in X/XO-induced oxidative damage

**Fig. 6:**
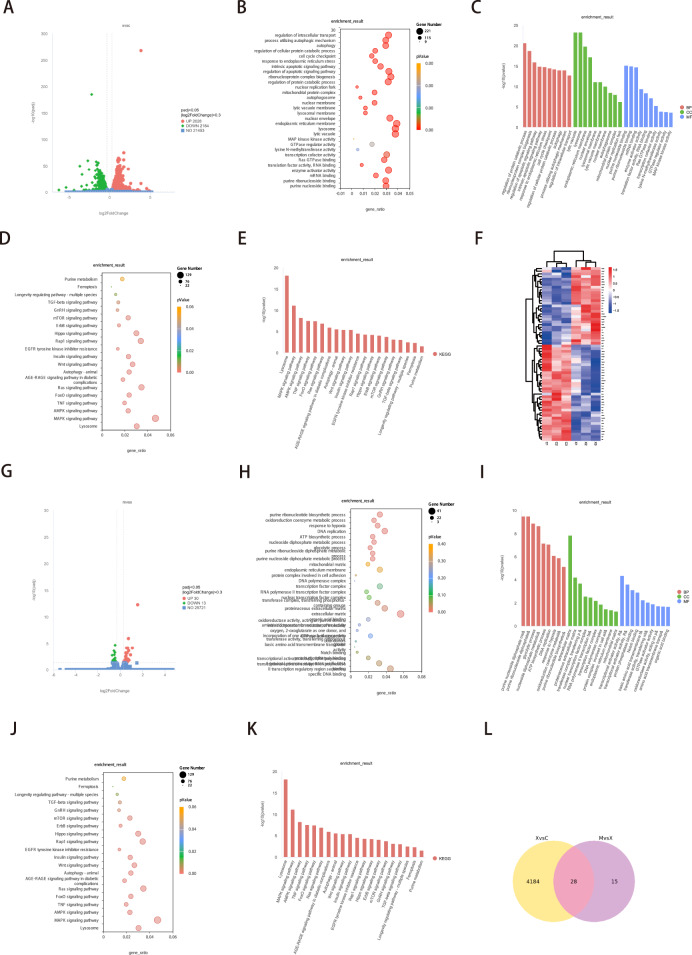
Transcriptome landscape suggests that autophagy was involved in X/XO-induced oxidative damage. **A** Volcano map of different genes between control and X/XO groups. **B** GO enrichment of differentially expressed genes between control and X/XO groups in dot map. **C** GO enrichment of differentially expressed genes between control and X/XO groups in bar graph. **D** Dot map of differentially expressed genes by KEGG pathway analysis of genes in (**A**). **E** Bar graph of differentially expressed genes by KEGG pathway analysis of genes in (**A**). **F** Clustering heat map of differentially expressed genes involved in the autophagy pathway. **G** Volcano map of different genes between X/XO and metformin treatment groups. **H** GO enrichment of differentially expressed genes between X/XO and metformin treatment groups in dot map. **I** GO enrichment of differentially expressed genes between X/XO and metformin treatment groups in bar graph. **J** Dot map of differentially expressed genes by KEGG pathway analysis of genes in (**G**). **K** Bar graph of differentially expressed genes by KEGG pathway analysis of genes in (**G**). **L** Venn map of differentially expressed gene intersection (padj < 0.05, |log2FoldChange| > 0.3).

To explore the downstream mechanism of X/XO-induced oxidative damage, transcriptome analysis was performed to compare the differential gene expression. A total of 2028 upregulated and 2184 downregulated genes were identified in the X/XO group compared with the control group (Fig. 6A and S1D). We performed GO enrichment and KEGG pathway analyses to explore pathogenesis mechanisms. GO analysis indicated that the DEGs were enriched in 'processing utilizing autophagic mechanism', 'autophagy', 'lysosomal membrane', 'lysosome', 'lytic vacuole' and 'autophagosome' (Fig. 6B, C). KEGG analysis also revealed enrichment of lysosome- and autophagy-related pathways (Fig. 6D, E). The above results indicated that there were significant changes in autophagy-related processes, including autophagosome generation and lysosomal phagocytosis, after X/XO treatment. We subsequently performed a cluster analysis of the gene expression profiles associated with the autophagy pathway. Compared with those in the control group, the expression levels of genes related to autophagosome formation, such as Zfyve1, Vmp1, Atg16l1 and Atg16l2; genes related to autophagosome membrane formation and transport, such as Atg9a and Atg9b; genes related to autophagosome formation and waste removal during autophagy, such as Sqstm1; and genes related to lysosomal membrane formation, such as Lamp1 and Lamp2, were significantly increased; and the expression levels of genes related to autophagosome membrane fusion, such as Sh3glb1, Ppp2cb, and Rab1a; and genes related to autophagosome and lysosome fusion, such as Rab7 and Rab7b, were significantly decreased in the treatment group (Fig. 6F). Additionally, we compared the changes in these parameters with those observed after metformin treatment. The results revealed 248 upregulated genes and 301 downregulated genes after metformin treatment (Fig. 6G). GO analysis also revealed that metformin can regulate purine ribonucleoside and oxidoreduction coenzyme metabolism (Fig. 6H, I). KEGG analysis revealed that the genes enriched in the FoxO signalling pathway and autophagy-related pathways were consistent with the above findings (Fig. 6J, K). To identify the factors involved in autophagy regulation by both X/XO and metformin, we constructed a Venn diagram of the DEGs and obtained 28 commonly expressed genes with padj < 0.05 and |log2FoldChange| > 0.3 (Fig. 6L, Table 3). We sorted the genes according to their differences and selected the top 15 genes for further screening. Nr4a1 is a potential target that mediates the autophagy process and can regulate autophagy in response to external stimuli during various physiological and pathological processes to maintain a stable internal environment [24].

**Table 3:** List of co-differentiated genes between groups in transcriptome analysis.

	gene_name	gene_description	MvsX_log2FoldChange	XvsC_log2FoldChange
ENSMUSG00000061848	Gm5805	predicted gene 5805 [Source:MGI Symbol;Acc:MGI:3644633]	1.071529075	−1.134933977
ENSMUSG00000083087	Gm11249	predicted gene 11249 [Source:MGI Symbol;Acc:MGI:3650834]	0.912088372	−0.950185922
ENSMUSG00000060548	Tnfrsf19	tumour necrosis factor receptor superfamily, member 19 [Source:MGI Symbol;Acc:MGI:1352474]	0.771762948	−1.831314692
ENSMUSG00000049929	Lpar4	lysophosphatidic acid receptor 4 [Source:MGI Symbol;Acc:MGI:1925384]	0.657736842	−0.332507986
ENSMUSG00000032715	Trib3	tribbles pseudokinase 3 [Source:MGI Symbol;Acc:MGI:1345675]	0.654435981	1.318439762
ENSMUSG00000027737	Slc7a11	solute carrier family 7 (cationic amino acid transporter, y+ system), member 11 [Source:MGI Symbol;Acc:MGI:1347355]	0.635420538	1.110717254
ENSMUSG00000027313	Chac1	ChaC, cation transport regulator 1 [Source:MGI Symbol;Acc:MGI:1916315]	0.612207784	1.157689283
ENSMUSG00000028339	Col15a1	collagen, type XV, alpha 1 [Source:MGI Symbol;Acc:MGI:88449]	0.609751946	−0.399997378
ENSMUSG00000021180	Rps6ka5	ribosomal protein S6 kinase, polypeptide 5 [Source:MGI Symbol;Acc:MGI:1920336]	0.527641827	−0.355616133
ENSMUSG00000023034	Nr4a1	nuclear receptor subfamily 4, group A, member 1 [Source:MGI Symbol;Acc:MGI:1352454]	0.524548556	−0.359572843
ENSMUSG00000047686	Rtl3	retrotransposon Gag like 3 [Source:MGI Symbol;Acc:MGI:2685221]	0.517725187	−0.602326877
ENSMUSG00000041313	Slc7a1	solute carrier family 7 (cationic amino acid transporter, y+ system), member 1 [Source:MGI Symbol;Acc:MGI:88117]	0.505591923	0.514537122
ENSMUSG00000040010	Slc7a5	solute carrier family 7 (cationic amino acid transporter, y+ system), member 5 [Source:MGI Symbol;Acc:MGI:1298205]	0.489381697	0.364840376
ENSMUSG00000014361	Mertk	c-mer proto-oncogene tyrosine kinase [Source:MGI Symbol;Acc:MGI:96965]	0.482258899	−0.302961594
ENSMUSG00000022346	Myc	myelocytomatosis oncogene [Source:MGI Symbol;Acc:MGI:97250]	0.478839652	−0.471298779
ENSMUSG00000005667	Mthfd2	methylenetetrahydrofolate dehydrogenase (NAD+ dependent), methenyltetrahydrofolate cyclohydrolase [Source:MGI Symbol;Acc:MGI:1338850]	0.475576757	0.594519236
ENSMUSG00000035783	Acta2	actin, alpha 2, smooth muscle, aorta [Source:MGI Symbol;Acc:MGI:87909]	0.44913732	−0.91500096
ENSMUSG00000028221	Pip4p2	phosphatidylinositol-4,5-bisphosphate 4-phosphatase 2 [Source:MGI Symbol;Acc:MGI:1919769]	0.429367375	−0.433505981
ENSMUSG00000031963	Bmper	BMP-binding endothelial regulator [Source:MGI Symbol;Acc:MGI:1920480]	0.417046122	−0.789411729
ENSMUSG00000074743	Thbd	thrombomodulin [Source:MGI Symbol;Acc:MGI:98736]	0.410622643	0.516347224
ENSMUSG00000026456	Cyb5r1	cytochrome b5 reductase 1 [Source:MGI Symbol;Acc:MGI:1919267]	0.344169423	0.968205153
ENSMUSG00000018906	P4ha2	procollagen-proline, 2-oxoglutarate 4-dioxygenase (proline 4-hydroxylase), alpha II polypeptide [Source:MGI Symbol;Acc:MGI:894286]	−0.437639509	0.88382691
ENSMUSG00000028463	Car9	carbonic anhydrase 9 [Source:MGI Symbol;Acc:MGI:2447188]	−0.490470519	1.239291632
ENSMUSG00000029307	Dmp1	dentin matrix protein 1 [Source:MGI Symbol;Acc:MGI:94910]	−0.57358663	0.884648979
ENSMUSG00000038065	Mturn	maturin, neural progenitor differentiation regulator homologue (Xenopus) [Source:MGI Symbol;Acc:MGI:1915485]	−0.603167677	−0.342211582
ENSMUSG00000064348	mt-Tn	mitochondrially encoded tRNA asparagine [Source:MGI Symbol;Acc:MGI:102479]	−0.665872122	0.833857608
ENSMUSG00000024186	Rgs11	regulator of G-protein signalling 11 [Source:MGI Symbol;Acc:MGI:1354739]	−0.73990227	1.102882757
ENSMUSG00000038393	Txnip	thioredoxin interacting protein [Source:MGI Symbol;Acc:MGI:1889549]	−1.495773276	−2.195567334

### Impaired autophagy flux mediates X/XO-induced oxidative damage in osteoblasts

**Fig. 7:**
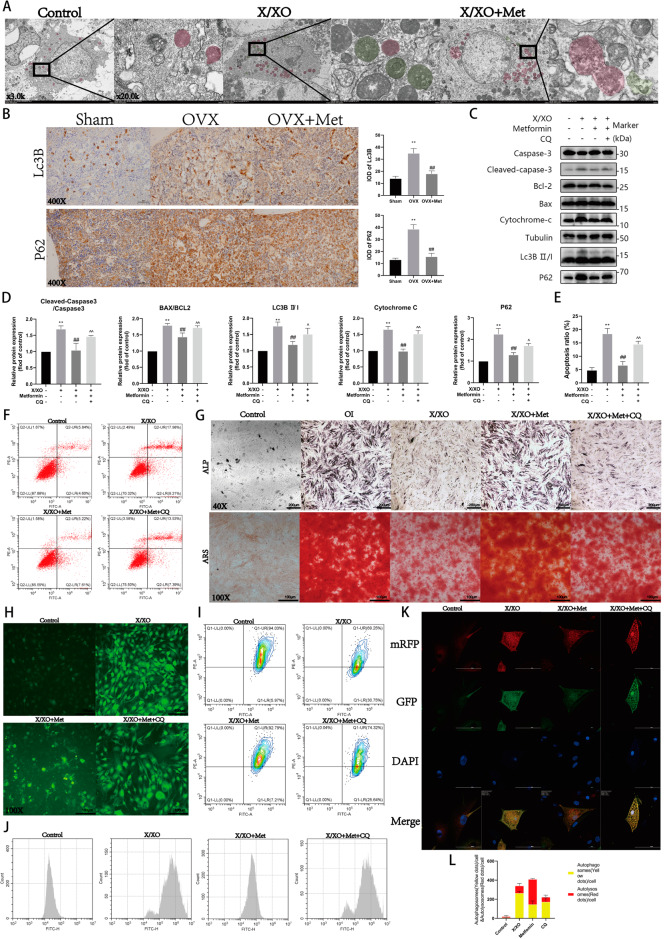
Impaired autophagy flux mediates X/XO-indued oxidative damage in osteoblasts. **A** Transmission electron microscopy for detection of autophagosome and autolysosome. **B** Immunohistochemistry and IOD (integrated optical density) of bone tissue after staining LC3B and p62 antibody. **C** Detection of mitochondrial apoptosis and autophagy related proteins by Western blotting. **D** Relative protein expression levels of the proteins in (**C**). **E** Statistical results of cell apoptosis rate in (**F**). **F** Cell apoptosis detected by flow cytometry with CQ treatment. **G** ALP and alizarin red staining detected with CQ treatment. **H** ROS levels detected by a fluorescent probe with CQ treatment. **I** Mitochondrial membrane potential levels after above treatment. **J** Flow cytometry detection of intracellular calcium ion staining related wavelengths. **K** Immunofluorescence analysis to observe the colocalization of GFP-LC3 and mRFP-LC3 puncta by laser confocal microscopy. **L** Quantitative detection analysis of autophagosomes (yellow dots in merged images) and autolysosomes (red dots in merged images). Experiments were implemented in triplicate. Data are means ± SDs, *p < 0.05, **p < 0.01 compared with control cells, #*p* < 0.05, ##*p* < 0.01 compared with X/XO and ^*p* < 0.05, ^^*p* < 0.01 compared with X/XO+Met applied analysed by using ANOVA.

Previously, we investigated the changes in autophagy and lysosomes during osteogenesis through transcriptome sequencing. First, transmission electron microscopy was used to verify the changes in autophagy and lysosomes. The results in the X/XO group suggested an increased ratio of green structures (autophagosomes) and a decreased ratio of purple structures (autolysosomes), which indicated that autophagic flux was blocked. Metformin greatly increased the number of autolysosomes and decreased the number of autophagosomes (Fig. 7A), which indicates that treatment with metformin alleviated the blocked autophagic flux caused by purine metabolism damage. An increased LC3BII/I indicates the occurrence of autophagy, and a decreased p62 indicates the degradation of autophagy. Thus, immunohistochemical analysis was performed to detect changes in the protein levels of LC3B and p62 at the animal level. Immunohistochemical analysis was performed to detect the number of LC3B- and p62-positive points. The expression of both autophagy markers was greater in the OVX group than in the sham group but was lower in the metformin treatment group, which was consistent with the results of the transcriptome analysis (Fig. 7B). Then, western blotting was used to detect the protein expression levels of p62 and LC3BII/I, which indicated an increase after X/XO treatment (Fig. 7C, D). The increase in LC3BII/I was considered to indicate autophagy activation, but the increase in p62 indicated that autophagic degradation was blocked. Furthermore, the changes in the LC3BII/I and p62 ratios were reversed by metformin, and these changes were blocked by the autophagy inhibitor CQ. CQ is a lysosome functional inhibitor that can inhibit autolysosome fusion and degradation and is used to study autophagic flux. Additionally, we detected osteoblast activity in response to CQ treatment. The results revealed that the therapeutic effects of metformin on apoptosis and differentiation were reversed by CQ (Fig. 7E, F, G). The increase in the expression of proteins related to mitochondrial apoptosis also indicated that autophagy was essential for the effect of metformin treatment (Fig. 7C, D). To evaluate the oxidative state in osteoblasts, we also detected the ROS level and mitochondrial membrane potential. Compared with metformin treatment, CQ addition increased intracellular ROS levels and decreased the mitochondrial membrane potential in the X/XO environment (Fig. 7H, I). The increase in the intracellular calcium concentration also indicated an increase in the degree of oxidative damage in response to CQ treatment (Fig. 7J). Additionally, osteoblasts were infected with mRFP-GFP-LC3 adenovirus to accurately observe and evaluate changes in autophagy. The results in the X/XO group suggested an increased ratio of yellow dots (autophagosomes) and a decreased ratio of red dots (autolysosomes), which indicated that autophagic flux was blocked. The change in the ratio of puncta was attenuated by metformin but reversed after CQ treatment (Fig. 7K, L). These results indicate that impaired autophagic flux is essential for X/XO-induced oxidative damage in osteoblasts.

### Metformin improves X/XO-induced oxidative damage by restoring Nr4a1-mediated autophagic flux

**Fig. 8:**
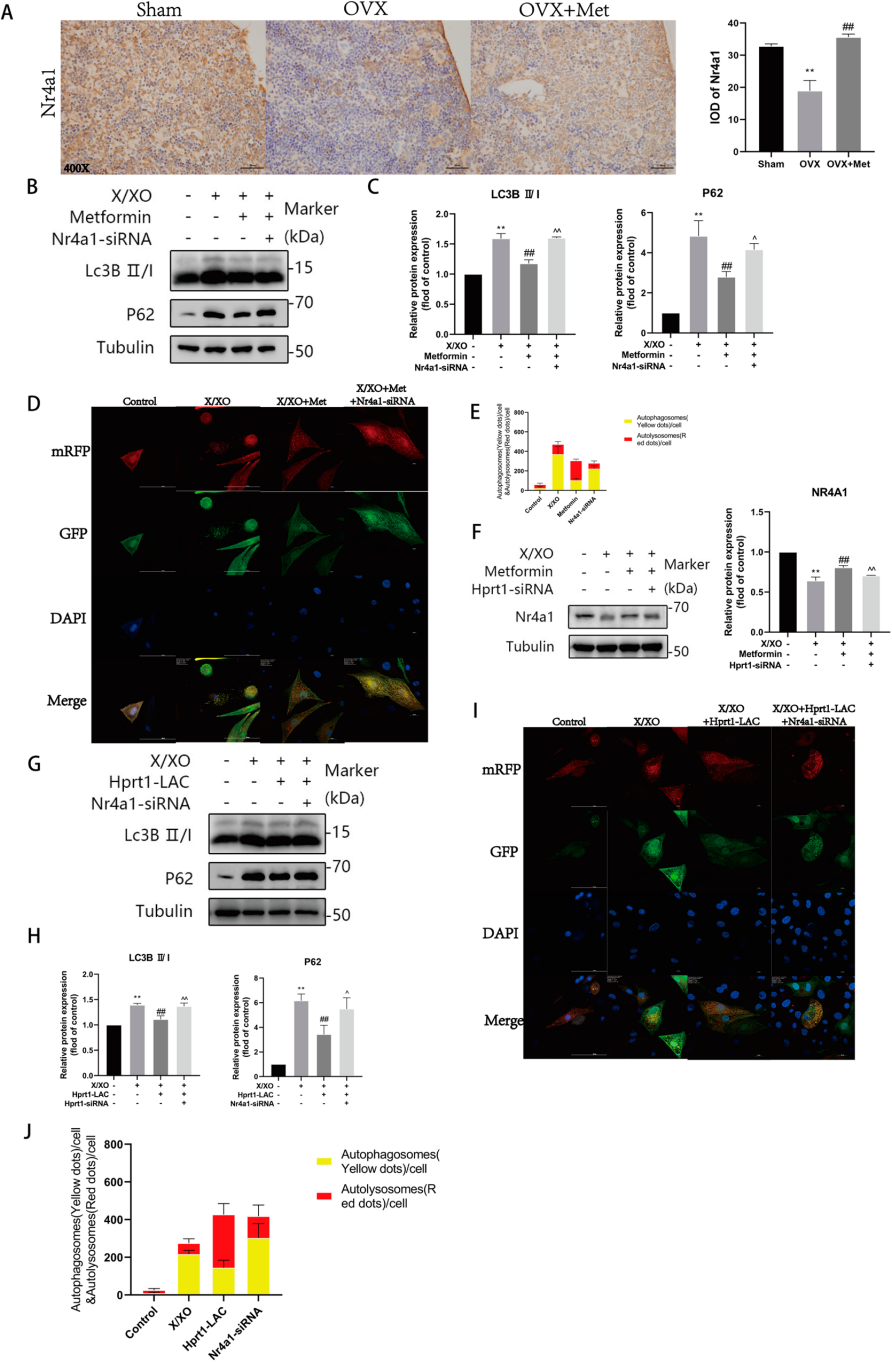
Metformin improved X/XO-induced oxidative damage by repairing Nr4a1-mediated autophagy flux. **A** Immunohistochemistry and IOD (integrated optical density) of bone tissue after staining Nr4a1 antibody. **B** Detection of autophagy related proteins by Western blotting after Nr4a1-siRNA transfection. **C** Relative protein expression levels of the proteins in (**B**). **D** Immunofluorescence analysis to observe the colocalization of GFP-LC3 and mRFP-LC3 puncta by laser confocal microscopy after Nr4a1-siRNA transfection. **E** Quantitative detection analysis of autophagosomes (yellow dots in merged images) and autolysosomes (red dots in merged images) after Nr4a1-siRNA transfection. **F** Detection of Nr4a1 protein by Western blotting with metformin and HPRT1-siRNA treatment and relative protein expression levels. **G** Detection of autophagy-related proteins by Western blotting after HPRT1-LAC and Nr4a1-siRNA transfection. **H** Relative protein expression levels of the proteins in (**G**). **I** Immunofluorescence analysis to observe the colocalization of GFP-LC3 and mRFP-LC3 puncta by laser confocal microscopy after HPRT1-LAC and Nr4a1-siRNA transfection. (J) Quantitative detection analysis of autophagosomes (yellow dots in merged images) and autolysosomes (red dots in merged images) after HPRT1-LAC and Nr4a1-siRNA transfection. Experiments were implemented in triplicate. Data are means ± SDs, *p < 0.05, **p < 0.01 compared with control cells, #*p* < 0.05, ##*p* < 0.01 compared with X/XO and ^*p* < 0.05, ^^*p* < 0.01 compared with X/XO+Met or LAC-HPRT1 applied analysed by using ANOVA.

To determine the role of Nr4a1 in the regulation of autophagy, we first performed immunohistochemical analysis to determine the Nr4a1 content in bone tissue. The results revealed that the number of Nr4a1-positive points was lower in the OVX group than in the sham group but was reversed in the metformin treatment group (Fig. 8A). We subsequently generated Nr4a1-silenced cells for further verification by adding Nr4a1-siRNA. Western blotting revealed that the protein expression levels of LC3BII/I and p62 were significantly increased in the Nr4a1-siRNA group (Fig. 8B, C). We further validated the changes in autophagic flux by infecting cells with mRFP-GFP-LC3 adenovirus. As shown in Fig. 8D, E, the change in the ratio of yellow dots (autophagosomes) to red dots (autolysosomes) was reversed by silencing the Nr4a1 gene. These results indicated that metformin repaired autophagic flux by increasing the expression of Nr4a1. Additionally, we further investigated the changes in Nr4a1 expression after HPRT1 silencing. The western blotting results indicated that the expression level of the Nr4a1 protein decreased with X/XO treatment and that the therapeutic effect of metformin was reversed after HPRT1 was silenced (Fig. 8F). Finally, HPRT1-overexpressing and Nr4a1-silenced cells were constructed to assess whether HPRT1 can further regulate autophagic flux via Nr4a1. Compared with that in the X/XO group, the expression of LC3BII/I and p62 decreased in the HPRT1-overexpressing group, and silencing of the Nr4a1 gene reversed these effects (Fig. 8G, H). The autophagic flux test results revealed a decreased proportion of yellow dots (autophagosomes) and an increased proportion of red dots (autolysosomes) after HPRT1 overexpression, and this effect was reversed by silencing the Nr4a1 gene (Fig. 8I, J).

## Discussion

Approximately one-third of postmenopausal women suffer from osteoporosis, and nearly half of these patients experience osteoporotic fractures [25, 26]. Understanding the pathogenesis of bone loss caused by oestrogen depletion as a high-risk factor will contribute to the development of prevention strategies to reduce the occurrence of severe complications of osteoporosis. On the basis of our micro-CT results, we collected bone tissue from sham and OVX mice and confirmed that OVX mice presented significant bone loss and microstructural destruction. Targeted (energy) metabolomics of bone tissue from the sham and OVX groups indicated that adenine was the top differentially abundant metabolite and that purine metabolism was a key metabolic process after enrichment analysis of the differentially abundant metabolites. On the basis of our previous conclusion that oxidative stress plays an important role in postmenopausal osteoporosis, we speculated that purine metabolism disorders are the fundamental cause of oxidative damage caused by oestrogen deficiency. Purine metabolites are important components of genetic material and energy units and determine genetic orientation and functional performance. Xanthine oxidase mediates the catabolism of purine metabolites with massive ROS generation [27]. Under physiological conditions, ROS are reduced to H_2_O_2_ by antioxidant enzymes within mitochondria. Excessive ROS accumulation disrupts the redox balance and causes opening of the mitochondrial permeability transition pore, which not only disrupts the mitochondrial membrane potential but also induces leakage of cytochrome C and induces caspase-mediated mitochondrial apoptosis [28]. Our research team added xanthine and xanthine oxidase to osteoblasts to construct a purine metabolism disorder model. The experimental results indicated increased apoptosis and weakened differentiation ability of osteoblasts. Further analysis of the oxidative status of osteoblasts revealed that the intracellular ROS levels were significantly increased and that mitochondrial membrane potential was reduced. These results suggest that purine metabolism reduces osteoblast activity by disrupting mitochondrial function and inducing intracellular peroxidation, which is consistent with the conclusions of the animal experiments.

Metformin has been shown to be a potential drug that delays ageing by regulating metabolism, repairing mitochondrial function, and reducing oxidative damage [29]. On the basis of our previous research and the mechanism of mitochondrial oxidative damage induced by purine metabolism disorders, we aimed to clarify the regulatory effect of metformin on purine metabolism in osteoblasts. First, we determined the therapeutic effect of metformin in the treatment of postmenopausal osteoporosis. Animal experiments indicated that intragastric administration of metformin increased bone mass in OVX mice. We collected serum samples from the mice and detected metabolite levels. Through network pharmacology analysis, we concluded that purine metabolism plays an important role in the treatment of metformin. Metformin improved oxidative states by regulating metabolic processes involving ROS. These results also confirmed the conclusion that disordered purine metabolism induces oxidative stress-induced damage and disrupts bone metabolism. We also predicted that HPRT1 is a potential target for metformin treatment of postmenopausal osteoporosis. HPRT1 is a hypoxanthine phosphoribosyl transferase involved in the purine salvage pathway. HPRT1 catalyses the conversion of hypoxanthine to inosine monophosphate and guanine to guanosine monophosphate via the transfer of the 5-phosphoribosyl group from 5-phosphoribosyl 1-pyrophosphate. HPRT1 deficiency leads to decreased purine anabolism and reduced mitochondrial membrane potential, which induces an increase in catabolism to produce and accumulate ROS [30, 31]. In cell experiments, we determined that osteogenic ability decreased when HPRT1 was deficient. Mitochondrial damage and ROS accumulation also contributed to osteoblast injury after decreasing HPRT1 expression. Metformin increased HPRT1 expression to reverse osteoblast apoptosis and improve osteogenic differentiation. On the basis of this gene function, HPRT1 is a promising intervention target for the regulation of purine metabolism to treat osteoporosis. Additionally, KEGG pathway analysis of the hub genes obtained via metabolomics and network pharmacology indicated that the FoxO1 signalling pathway plays an important role in the regulation of HPRT1-mediated purine metabolism. The FoxO1 signalling pathway is associated with bone degeneration, and bone is lost significantly when FoxO1 is inhibited [32]. The precise control of FoxO1 subcellular localization and function can be precisely regulated by posttranslational modifications in response to different environmental stresses and stimuli [33]. Our study also aimed to clarify the epigenetic modifications of FoxO1 through validation of the regulatory effect of metformin on the FoxO1 nuclear-cytoplasmic shuttle. The deacetylase genes of the sirtuin family have been confirmed to be involved in the localization of FoxO1 and affect its function [34]. Compared to unmodified FoxO1, acetylated FoxO1 is more susceptible to phosphorylation, which promotes FoxO1 cytoplasmic localization [35, 36]. The deacetylation complex formed by FoxO1 combined with sirtuin genes was shown to resist oxidative stress [37]. X/XO treatment inhibited FoxO1 translocation from the cytoplasm to the nucleus. Our previous study demonstrated that SIRT3 could improve oxidative stress induced by mitochondrial damage in osteoblasts [23]. After the transfection of SIRT3-siRNA, the content of FoxO1 decreased in the nucleus and increased in the cytoplasm, which indicated that SIRT3-mediated deacetylation modulates the nucleocytoplasmic shuttling ability of FoxO1 and promotes its nuclear localization. Deacetylated FoxO1 has a stronger ability to bind DNA to promote the transcriptional activity of its downstream proteins [38]. FoxO1 deacetylation with SIRT3 upregulated HPRT1 expression to repair purine metabolism.

After elucidating the mechanism by which purine metabolism disorder-induced mitochondrial dysfunction leads to intracellular oxidative stress, we aimed to further reveal the reasons for the oxidative damage-mediated decline in osteoblast function. Transcriptome sequencing was performed to detect the differentially expressed genes in osteoblasts after X/XO and metformin treatment. Enrichment analysis of these genes indicated that autophagy and lysosomal activity are involved in the pathological changes induced by X/XO and the therapeutic effect of metformin. Autophagy is a catabolic process in which damaged or aged cytoplasmic components are degraded and renewed by packaging protein complexes and organelles and is the mechanism by which cells survive under stressful conditions [39]. In this process, these components are encapsulated in double-membrane vesicles to form autophagosomes. These autophagosomes further bind to lysosomes, and their contents are degraded by lysosomal enzymes [40]. In bone metabolism, elevated autophagy contributes to increasing the expression of osteogenic markers to promote osteoblast differentiation and mineralization [41]. The activation of autophagy was also confirmed to increase bone mass after OVX [42]. LC3B and p62 are essential genes involved in the regulation of autophagy. LC3B participates in the autophagy process after conversion from LC3B I to LC3B II [43]. LC3B II is located on the autophagosome membrane and is a marker of the number of autophagosomes. P62 is considered a substrate that binds directly to LC3B. The autophagosome is identified and degraded by lysosomes after the binding of LC3B and p62 to the autophagosome membrane [44]. P62 levels represent the degree of autophagic flux and the smoothness of the autophagy process. After X/XO treatment, the LC3B II/I ratio was significantly increased, but the expression of p62 was also increased, which indicated that the newly synthesized autophagosomes were not degraded by lysosomes. Purine metabolism disorders might inhibit the fusion of autophagosomes and lysosomes or their degradation to disrupt autophagic flux and block the autophagy process. To determine the effect of autophagy, we also transfected cells with the mRFP-GFP-LC3 adenovirus, and the results were consistent with the changes in the expression of related proteins. Additionally, we screened for DEGs via transcriptome sequencing and found that Nr4a1 was an important gene involved in the regulation of autophagy. Nr4a1 is a nuclear transcription factor involved in the response to pathological changes. There was a specific interaction between Nr4a1 and p62, which is translocated to mitochondria from the nucleus to induce autophagy [24]. X/XO significantly inhibited the expression of Nr4a1, and the therapeutic effect of metformin was reversed after Nr4a1-siRNA was added to osteoblasts. Nr4a1 silencing also blocked autophagic flux. Therefore, Nr4a1-mediated autophagy plays an important role in X/XO-induced osteoblast damage.

Our study investigated differences in the levels of bone energy metabolites and elucidated the mechanism of bone imbalance caused by purine metabolism disorders in vitro and in vivo. Oxidative stress-induced damage caused by ROS produced from purine metabolism is important for the pathogenesis of postmenopausal osteoporosis. Serum metabolomics combined with network pharmacology revealed the pharmacological mechanism of metformin in the treatment of postmenopausal osteoporosis. HPRT1 is the key regulatory target for repairing purine metabolism. SIRT3-mediated deacetylation of FoxO1 modulates its nucleocytoplasmic shuttling and contributes to increasing the expression of HPRT1. Additionally, transcriptome sequencing analysis indicated that autophagy participates in the regulation of oxidative damage in osteoblasts. The intracellular blockage of autophagy exacerbates disordered purine metabolism. Increasing the expression of Nr4a1 improved autophagic flux and reversed osteoblast apoptosis. The above results reveal important factors in the pathogenesis of postmenopausal osteoporosis, suggest therapeutic targets and describe the regulatory network. Our conclusions may be used to optimize therapeutic methods and promote drug development to increase bone mass in postmenopausal women.

There are several limitations to our study. The purine metabolism process can be broken down into a balance between XO-mediated catabolism and HPRT1-mediated synthetic metabolism. Our in vivo experimental results suggest that HPRT1 expression did not significantly change in the bone tissue of postmenopausal mice but increased with metformin treatment. HPRT1 silencing also reversed the therapeutic effect of metformin on X/XO-mediated oxidative damage in osteoblasts. It can be inferred that HPRT1 is an effective therapeutic target, but its role in the pathogenesis of postmenopausal osteoporosis remains to be confirmed. Additionally, XO is more effective at simulating purine metabolism disorders in vitro, but the in vitro model results did not effectively reflect the results of in vivo experiments. Our research group will explore the key role and regulatory network of HPRT1 in the pathogenesis of osteoporosis in subsequent studies by constructing HPRT1 knockout mice and primary osteoblast HPRT1 silencing models.

## Supplementary information

Supplementary-FigureS1

Supplementary-Legends

WB-unprocessed-blots

SupplementaryDocuments

## Data Availability

The datasets used and/or analyzed during the current study are available from the corresponding author on reasonable request.
