# Supplementary figures and images for: Metformin improves HPRT1-targeted purine metabolism and repairs NR4A1-mediated autophagic flux by modulating FoxO1 nucleocytoplasmic shuttling to treat postmenopausal osteoporosis
Source: Cell Death Dis. 2024 Nov 6;15(11):795. doi: 10.1038/s41419-024-07177-5 (PMC11538437; doi:10.1038/s41419-024-07177-5)

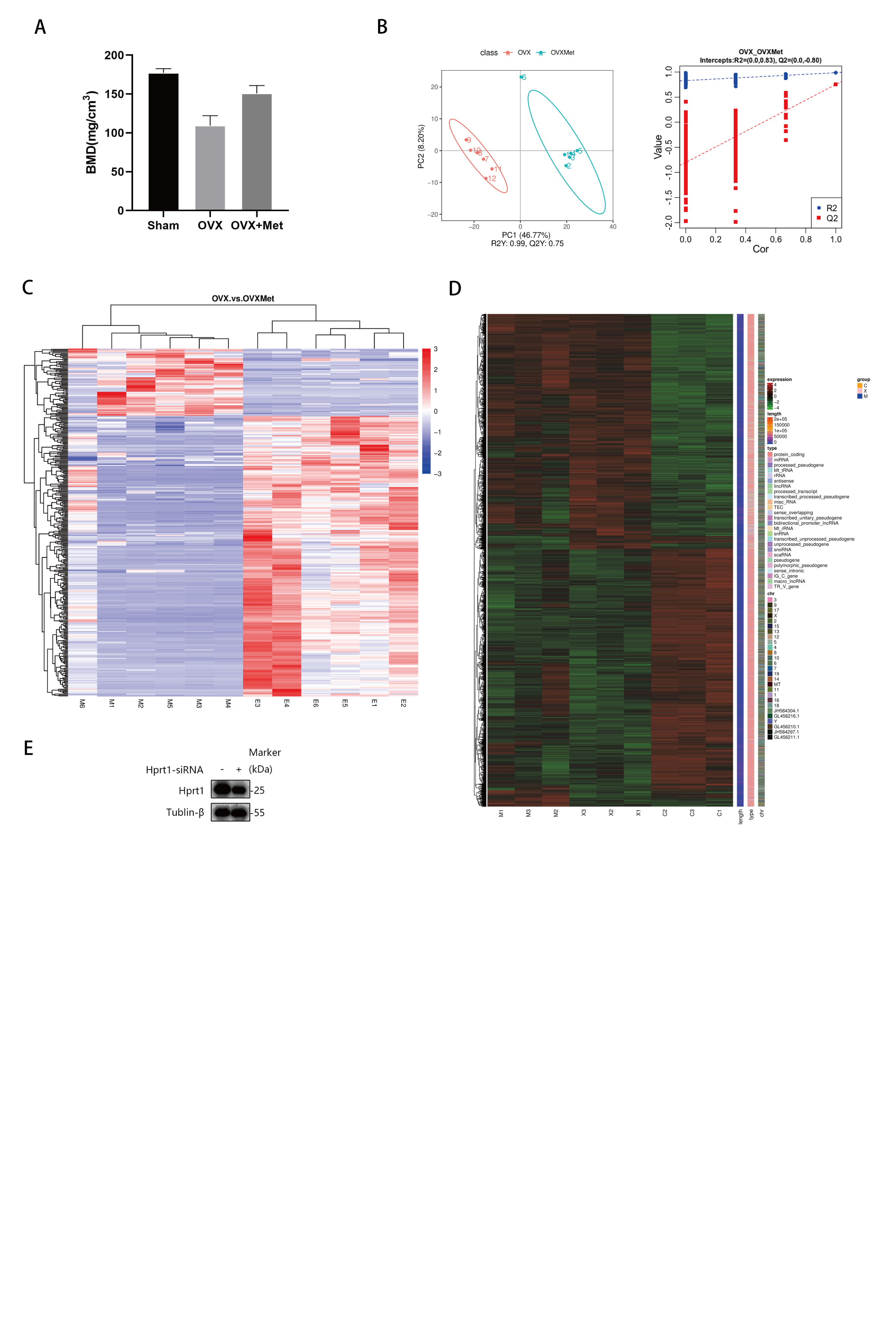

Supplement: Supplementary file 1 — Supplementary-FigureS1 [file 41419_2024_7177_MOESM1_ESM.png]
