# Supplementary material for: Metformin improves HPRT1-targeted purine metabolism and repairs NR4A1-mediated autophagic flux by modulating FoxO1 nucleocytoplasmic shuttling to treat postmenopausal osteoporosis
Source: Cell Death Dis. 2024 Nov 6;15(11):795. doi: 10.1038/s41419-024-07177-5 (PMC11538437; doi:10.1038/s41419-024-07177-5)
Supplement: Supplementary file 2 — Supplementary-Legends [file 41419_2024_7177_MOESM2_ESM.docx]

Supplementary Figure 1 Bone energy metabolism-targeted metabolomic and transcriptome analysis

(A) Statistical results of BMD (mg/cm^3^) of mice in Figure2A

(B) Least partial squares discriminant analysis (OPLS-DA) of differential metabolites in serum between OXV group and metformin treatment group

(C) Difference clustering heat map of serum differential metabolites between OXV group and metformin treatment group
(D) The differential gene clustering heat map in the transcriptome analysis results in Figure 6

(E) Construction of Hprt1 silenced cell line and detection of silencing effect of related proteins in Figure5
