# Supplementary material for: Metformin improves HPRT1-targeted purine metabolism and repairs NR4A1-mediated autophagic flux by modulating FoxO1 nucleocytoplasmic shuttling to treat postmenopausal osteoporosis
Source: Cell Death Dis. 2024 Nov 6;15(11):795. doi: 10.1038/s41419-024-07177-5 (PMC11538437; doi:10.1038/s41419-024-07177-5)

All blots are automatically exposed by the DNR system (Bio-Imaging Systems Ltd.) under the marker light after being fully soaked in ECL luminescent solution (MAO186-1 Meilunbio™).
All Western blot experiments were performed in triplicate.
Prestained protein ladders were obtained from Thermo Fisher Scientific™ (26616).
The protein name is followed by the predicted molecular weight (kDa) of the protein.
The numbers in the original image is the molecular weight marker (kDa) of the Prestained protein ladders.

Figure 4 B

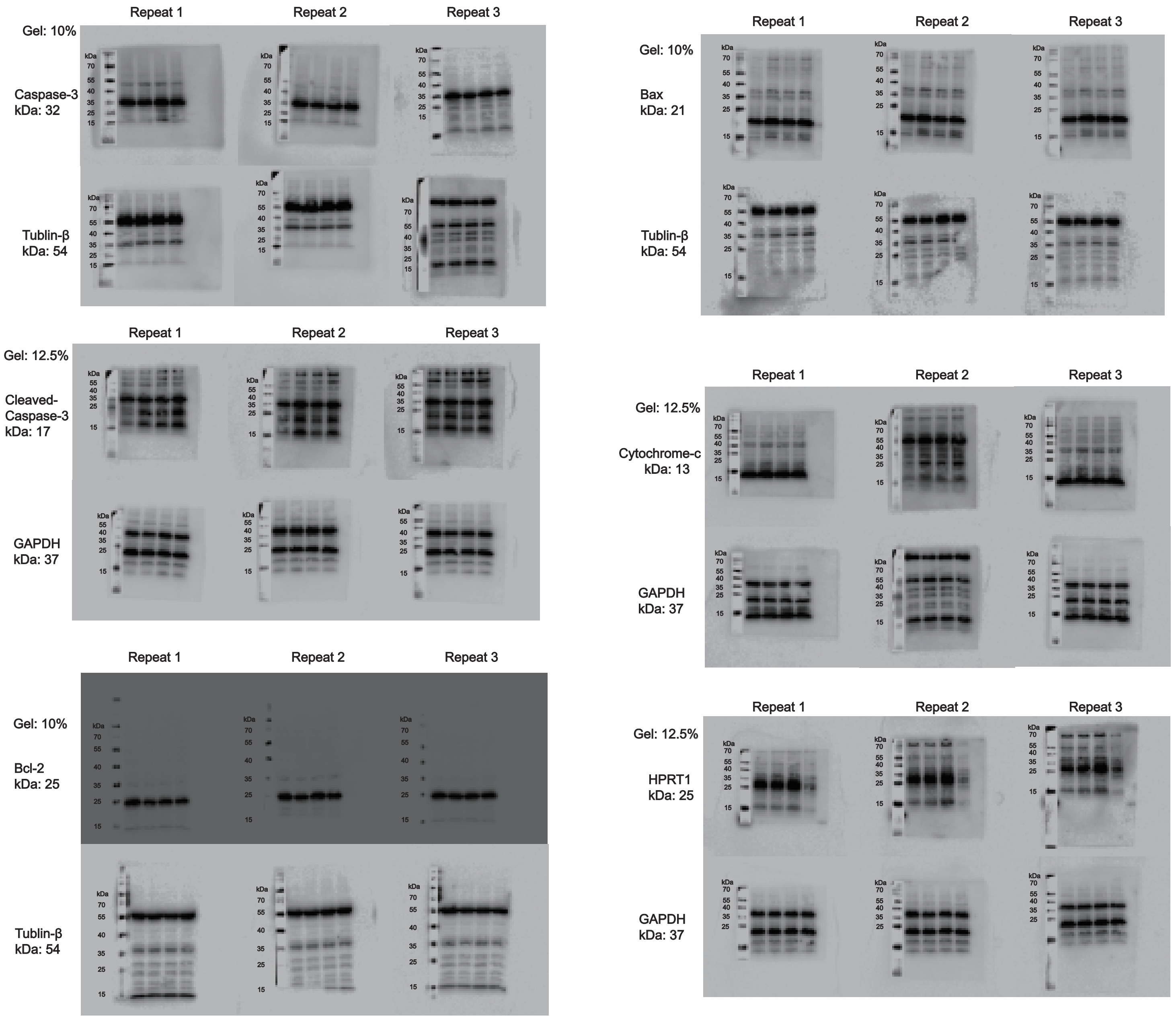

Figure 5 B

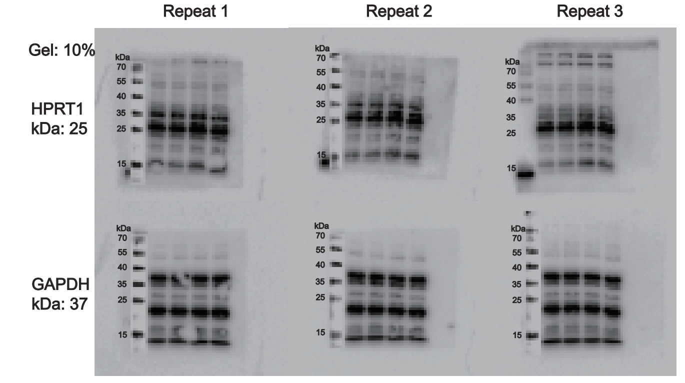

Figure 5 D

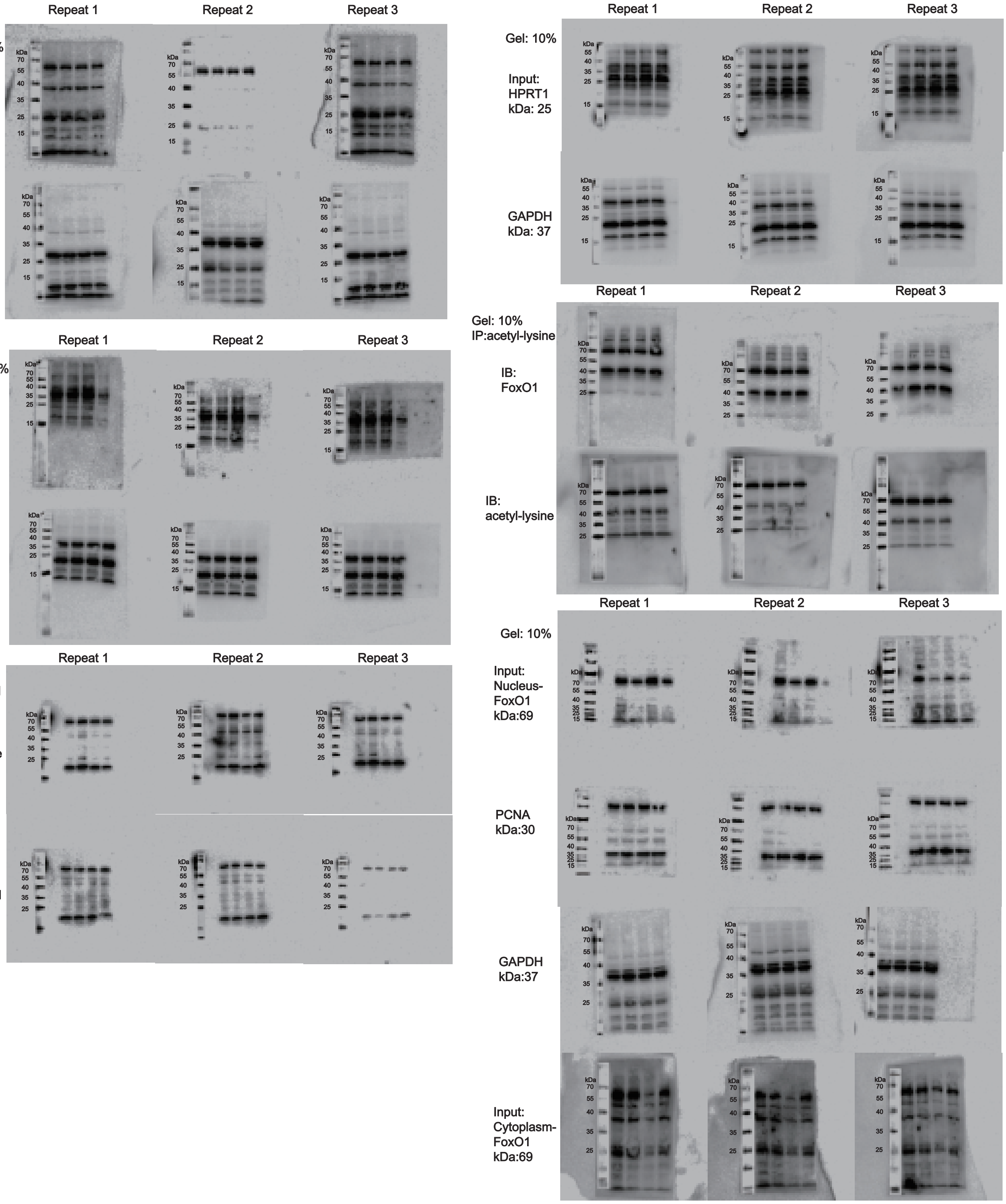

Figure 5 I

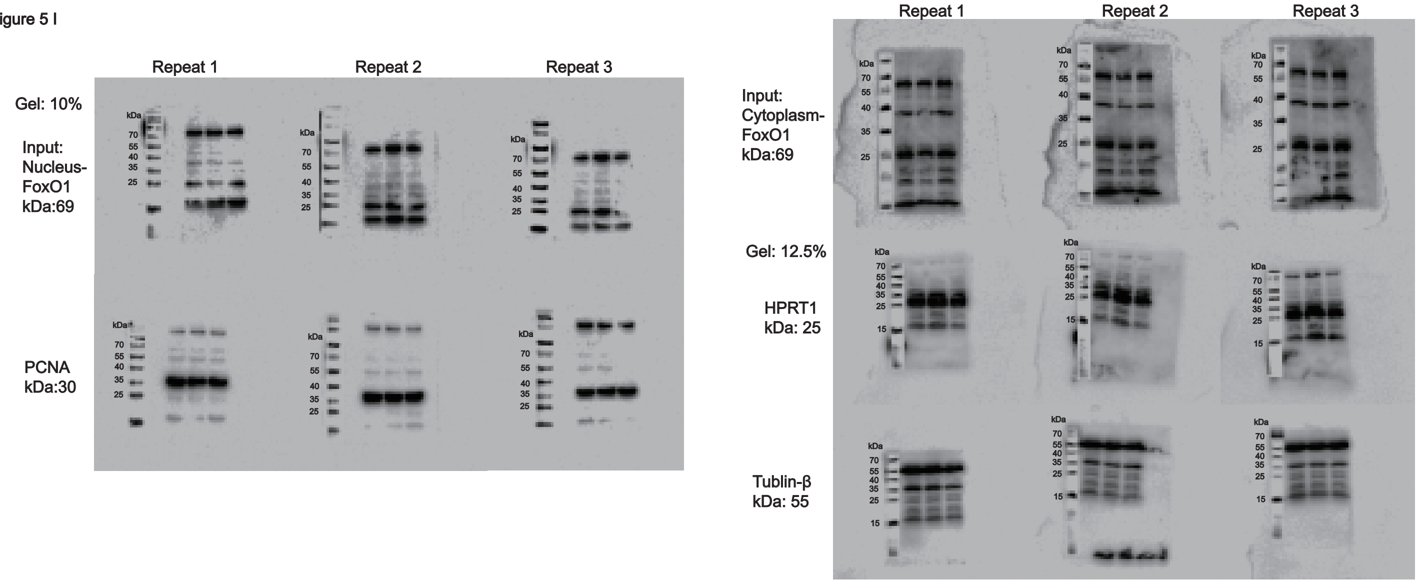

Figure 7 C

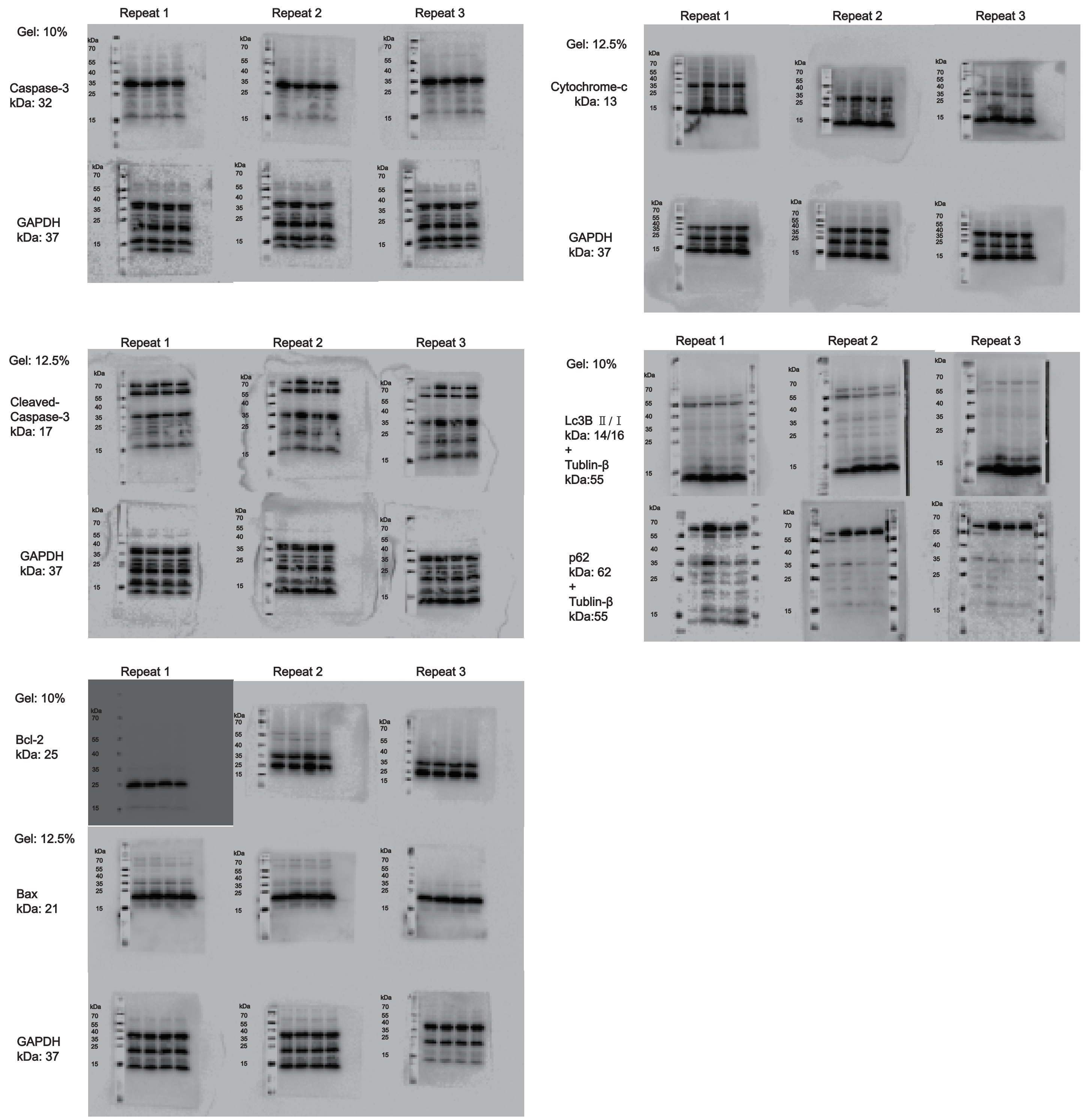
Repeat 1
Repeat 2
Repeat 3
Gel: 10%
Caspase-3
kDa: 32
GAPDH
kDa: 37
Gel: 12.5%
Cytochrome-c
kDa: 13
GAPDH
kDa: 37
Gel: 12.5%
Cleaved-Caspase-3
kDa: 17
GAPDH
kDa: 37
Gel: 10%
Lc3B Ⅱ/Ⅰ
kDa: 14/16
+
Tublin-β
kDa:55
p62
kDa: 62
+
Tublin-β
kDa:55
Gel: 10%
Bcl-2
kDa: 25
Gel: 12.5%
Bax
kDa: 21
GAPDH
kDa: 37
kDa
70
55
40
35
25
15

Figure 8 B

Gel: 10%

Lc3B Ⅱ/Ⅰ
kDa: 14/16

Repeat 1 Repeat 2 Repeat 3

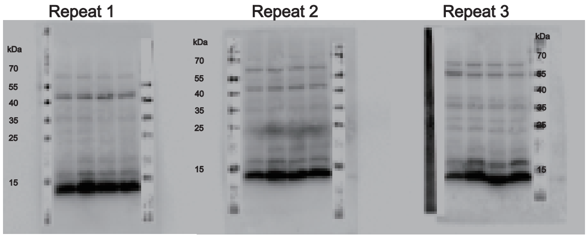

p62
kDa: 62

Repeat 1 Repeat 2 Repeat 3

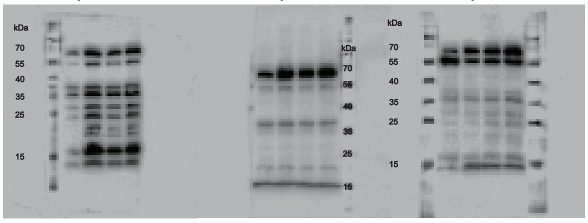

Figure 8 F

Gel: 10%

Nr4a1
kDa:64

Tublin-β
kDa:55

Repeat 1 Repeat 2 Repeat 3

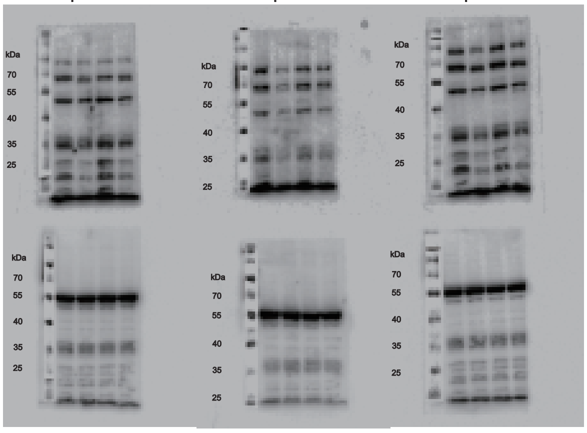

Figure 8 G

Gel: 10%

Lc3B Ⅱ/Ⅰ
kDa: 14/16

Repeat 1 Repeat 2 Repeat 3

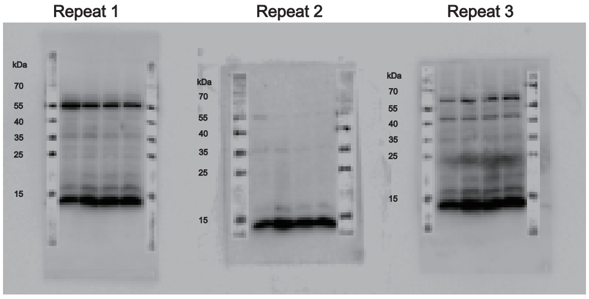

p62
kDa: 62

Repeat 1 Repeat 2 Repeat 3

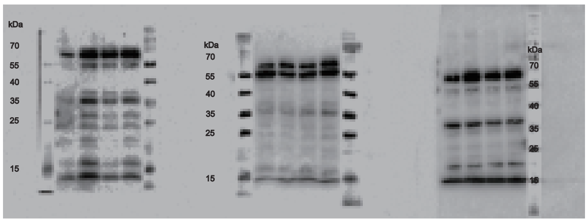

Supplement: Supplementary file 3 — WB-unprocessed-blots [file 41419_2024_7177_MOESM3_ESM.pdf]
