# Supplementary material for: Metformin improves HPRT1-targeted purine metabolism and repairs NR4A1-mediated autophagic flux by modulating FoxO1 nucleocytoplasmic shuttling to treat postmenopausal osteoporosis
Source: Cell Death Dis. 2024 Nov 6;15(11):795. doi: 10.1038/s41419-024-07177-5 (PMC11538437; doi:10.1038/s41419-024-07177-5)
Supplement: Supplementary file 4 — SupplementaryDocuments [file 41419_2024_7177_MOESM4_ESM.pdf]

GO

	ONTOLOG	ID	Descriptio	GeneRatio	BgRatio	pvalue	p.adjust	qvalue	geneID	Count	number	term
GO:20003	BP	GO:20003	regulation	9/34	195/18670	5.15E-11	5.28E-08	2.81E-08	BMP7/F2/	9	30	regulation of reactive oxygen…
GO:00725	BP	GO:00725	reactive o	10/34	284/18670	5.38E-11	5.28E-08	2.81E-08	BMP7/F2/	10	29	reactive oxygen species metabolic…
GO:20003	BP	GO:20003	positive re	7/34	102/18670	5.62E-10	3.67E-07	1.96E-07	F2/GSTP1/	7	28	positive regulation of reactive…
GO:00226	BP	GO:00226	extracellul	6/34	80/18670	6.25E-09	3.06E-06	1.63E-06	CTSK/DPP	6	27	extracellular matrix disassembly
GO:00305	BP	GO:00305	collagen c	5/34	47/18670	2.14E-08	8.39E-06	4.47E-06	CTSK/MM	5	26	collagen catabolic process
GO:00329	BP	GO:00329	collagen m	6/34	115/18670	5.60E-08	1.83E-05	9.74E-06	CTSK/F2/	6	25	collagen metabolic process
GO:00507	BP	GO:00507	regulation	9/34	485/18670	1.47E-07	4.12E-05	2.20E-05	F2/GSTP1/	9	24	regulation of inflammatory response
GO:00518	BP	GO:00518	regulation	7/34	244/18670	2.38E-07	5.83E-05	3.11E-05	F10/HSP9	7	23	regulation of protein kinase…
GO:00434	BP	GO:00434	protein ki	7/34	269/18670	4.60E-07	0.0001	5.34E-05	F10/HSP9	7	22	protein kinase B signaling
GO:00518	BP	GO:00518	positive re	6/34	176/18670	6.96E-07	0.000137	7.27E-05	F10/HSP9	6	21	positive regulation of protein…
GO:19029	CC	GO:19029	protein ki	5/34	109/19717	1.15E-06	9.49E-05	5.63E-05	IGF1R/INS	5	20	protein kinase complex
GO:19048	CC	GO:19048	ficolin-1-r	5/34	124/19717	2.18E-06	9.49E-05	5.63E-05	CANT1/GS	5	19	ficolin-1-rich granule lumen
GO:01010	CC	GO:01010	ficolin-1-r	5/34	185/19717	1.54E-05	0.000446	0.000264	CANT1/GS	5	18	ficolin-1-rich granule
GO:00616	CC	GO:00616	transferas	5/34	259/19717	7.67E-05	0.001668	0.000989	IGF1R/INS	5	17	transferase complex, transferring phosph…
GO:19047	CC	GO:19047	tertiary gr	3/34	55/19717	0.000116	0.002011	0.001192	CANT1/M	3	16	tertiary granule lumen
GO:00708	CC	GO:00708	tertiary gr	4/34	164/19717	0.000176	0.002344	0.00139	CANT1/M	4	15	tertiary granule
GO:00347	CC	GO:00347	secretory	5/34	321/19717	0.000209	0.002344	0.00139	CANT1/GS	5	14	secretory granule lumen
GO:00315	CC	GO:00315	nucleotide	2/34	14/19717	0.000259	0.002344	0.00139	PRKAA2/P	2	13	nucleotide-activated protein kinase comp…
GO:00602	CC	GO:00602	cytoplasmi	5/34	338/19717	0.000266	0.002344	0.00139	CANT1/GS	5	12	cytoplasmic vesicle lumen
GO:00319	CC	GO:00319	vesicle lu	5/34	339/19717	0.000269	0.002344	0.00139	CANT1/GS	5	11	vesicle lumen
GO:00041	MF	GO:00041	endopepti	11/34	427/17697	2.46E-10	4.19E-08	2.36E-08	CASP1/CT	11	10	endopeptidase activity
GO:00042	MF	GO:00042	serine-typ	8/34	160/17697	5.57E-10	4.73E-08	2.67E-08	DPP4/F10/	8	9	serine-type endopeptidase activity
GO:00082	MF	GO:00082	serine-typ	8/34	182/17697	1.55E-09	7.82E-08	4.41E-08	DPP4/F10/	8	8	serine-type peptidase activity
GO:00171	MF	GO:00171	serine hyd	8/34	186/17697	1.84E-09	7.82E-08	4.41E-08	DPP4/F10/	8	7	serine hydrolase activity
GO:00051	MF	GO:00051	insulin-lik	3/34	16/17697	3.57E-06	0.000121	6.83E-05	INSR/IRS1/	3	6	insulin-like growth factor receptor…
GO:00435	MF	GO:00435	phosphati	3/34	30/17697	2.54E-05	0.000719	0.000405	IGF1R/INS	3	5	phosphatidylinositol 3-kinase binding
GO:00042	MF	GO:00042	metalloen	4/34	103/17697	4.39E-05	0.001066	0.0006	MMP1/M	4	4	metalloendopeptidase activity
GO:00167	MF	GO:00167	transferas	3/34	58/17697	0.000186	0.00331	0.001865	FDPS/GST	3	3	transferase activity, transferring alkyl…
GO:00432	MF	GO:00432	glutathion	2/34	11/17697	0.000195	0.00331	0.001865	GSTM1/GS	2	2	glutathione binding
GO:00435	MF	GO:00435	insulin rec	2/34	11/17697	0.000195	0.00331	0.001865	IGF1R/INS	2	1	insulin receptor substrate binding

KEGG

ID	Descriptioı	GeneRatio	BgRatio	pvalue	p.adjust	qvalue	geneID	Count
hsa04068	FoxO signa	8/33	131/8108	3.71E-08	6.30E-06	3.74E-06	IGF1R/INS	8
hsa04920	Adipocyto	6/33	69/8108	2.81E-07	2.30E-05	1.37E-05	IRS1/LEP/N	6
hsa04152	AMPK sigr	7/33	120/8108	4.05E-07	2.30E-05	1.37E-05	IGF1R/INS	7
hsa05418	Fluid shea	7/33	139/8108	1.10E-06	4.38E-05	2.60E-05	GSTM1/GS	7
hsa04211	Longevity	6/33	89/8108	1.29E-06	4.38E-05	2.60E-05	IGF1R/INS	6
hsa04657	IL-17 signa	6/33	94/8108	1.78E-06	4.87E-05	2.90E-05	HSP90AA1	6
hsa05215	Prostate ca	6/33	97/8108	2.14E-06	4.87E-05	2.90E-05	GSTP1/HS	6
hsa04932	Non-alcoh	7/33	155/8108	2.29E-06	4.87E-05	2.90E-05	INSR/IRS1/	7
hsa05171	Coronaviru	8/33	232/8108	2.98E-06	5.63E-05	3.35E-05	CASP1/F2/	8
hsa04213	Longevity	5/33	62/8108	4.47E-06	7.59E-05	4.51E-05	IGF1R/INS	5
hsa05417	Lipid and a	7/33	215/8108	1.99E-05	0.000307	0.000182	CASP1/HS	7
hsa04625	C-type lec	5/33	104/8108	5.62E-05	0.000797	0.000474	CASP1/IL2	5
hsa04659	Th17 cell c	5/33	107/8108	6.44E-05	0.000818	0.000486	HSP90AA1	5
hsa04931	Insulin resi	5/33	108/8108	6.74E-05	0.000818	0.000486	INSR/IRS1/	5
hsa05415	Diabetic ca	6/33	203/8108	0.000144	0.001632	0.00097	INSR/IRS1/	6
hsa04926	Relaxin sig	5/33	129/8108	0.000157	0.001664	0.000989	MAPK14/N	5
hsa04917	Prolactin s	4/33	70/8108	0.000173	0.001726	0.001026	CYP17A1/I	4
hsa04910	Insulin sigr	5/33	137/8108	0.000208	0.001961	0.001166	INSR/IRS1/	5
hsa05133	Pertussis	4/33	76/8108	0.000237	0.002019	0.0012	CASP1/MA	4
hsa04140	Autophagy	5/33	141/8108	0.000238	0.002019	0.0012	IGF1R/IRS1	5
hsa04150	mTOR sigr	5/33	155/8108	0.000369	0.002984	0.001774	IGF1R/INS	5
hsa04151	PI3K-Akt s	7/33	354/8108	0.000455	0.003517	0.00209	HSP90AA1	7
hsa04658	Th1 and Th	4/33	92/8108	0.000494	0.00365	0.00217	IL2/MAPK1	4
hsa01522	Endocrine	4/33	98/8108	0.000628	0.004445	0.002642	IGF1R/MA	4
hsa04914	Progesterc	4/33	100/8108	0.000677	0.004606	0.002738	HSP90AA1	4
hsa05142	Chagas dis	4/33	102/8108	0.00073	0.004719	0.002805	IL2/MAPK1	4
hsa04621	NOD-like	5/33	181/8108	0.000749	0.004719	0.002805	CASP1/HS	5
hsa04620	Toll-like re	4/33	104/8108	0.000785	0.004768	0.002834	CTSK/MAF	4
hsa04930	Type II dia	3/33	46/8108	0.000828	0.004852	0.002884	INSR/IRS1/	3
hsa04668	TNF signal	4/33	112/8108	0.001037	0.005685	0.003379	MAPK14/N	4
hsa05145	Toxoplasm	4/33	112/8108	0.001037	0.005685	0.003379	MAPK14/N	4
hsa04913	Ovarian st	3/33	51/8108	0.00112	0.00595	0.003537	CYP17A1/I	3
hsa04935	Growth ho	4/33	119/8108	0.001299	0.006693	0.003978	IRS1/MAPI	4
hsa04380	Osteoclast	4/33	128/8108	0.001701	0.008505	0.005056	CTSK/MAF	4
hsa05135	Yersinia in	4/33	137/8108	0.002182	0.010601	0.006301	CASP1/IL2	4
hsa05140	Leishmania	3/33	77/8108	0.003661	0.01719	0.010218	MAPK14/N	3
hsa04217	Necroptos	4/33	159/8108	0.003741	0.01719	0.010218	CASP1/HS	4
hsa05161	Hepatitis B	4/33	162/8108	0.004	0.017777	0.010567	MAPK14/N	4
hsa00983	Drug meta	3/33	80/8108	0.004078	0.017777	0.010567	GSTM1/GS	3
hsa04530	Tight junct	4/33	169/8108	0.004651	0.019768	0.011751	MAPK8/PF	4
hsa04610	Compleme	3/33	85/8108	0.004835	0.02005	0.011918	F10/F2/PL/	3
hsa05152	Tuberculos	4/33	180/8108	0.005814	0.023531	0.013988	MAPK14/N	4
hsa05323	Rheumato	3/33	93/8108	0.006214	0.024568	0.014604	CTSK/MMI	3
hsa04710	Circadian r	2/33	31/8108	0.006939	0.02681	0.015936	PRKAA2/P	2
hsa05202	Transcripti	4/33	192/8108	0.007288	0.027533	0.016366	IGF1R/MM	4
hsa04933	AGE-RAGE	3/33	100/8108	0.007596	0.028071	0.016686	MAPK14/N	3
hsa05130	Pathogeni	4/33	197/8108	0.007969	0.028826	0.017135	CASP1/F2/	4
hsa04660	T cell recep	3/33	104/8108	0.00846	0.029964	0.017812	IL2/MAPK1	3
hsa05205	Proteoglyc	4/33	205/8108	0.009145	0.031729	0.01886	IGF1R/MA	4
hsa04066	HIF-1 sign	3/33	109/8108	0.00962	0.032628	0.019395	IGF1R/INS	3
hsa04960	Aldosteror	2/33	37/8108	0.009788	0.032628	0.019395	INSR/IRS1	2
hsa05219	Bladder ca	2/33	41/8108	0.01193	0.039003	0.023185	MMP1/MN	2
hsa04722	Neurotrop	3/33	119/8108	0.012207	0.039156	0.023275	IRS1/MAPI	3
hsa05132	Salmonella	4/33	249/8108	0.017654	0.054982	0.032683	CASP1/HS	4
hsa04371	Apelin sigr	3/33	137/8108	0.017788	0.054982	0.032683	NOS2/PRK	3
hsa05162	Measles	3/33	139/8108	0.018483	0.056109	0.033353	IL2/MAPK8	3
hsa00480	Glutathion	2/33	57/8108	0.022301	0.065366	0.038855	GSTM1/GS	2
hsa04923	Regulation	2/33	57/8108	0.022301	0.065366	0.038855	INSR/IRS1	2
hsa04630	JAK-STAT	3/33	162/8108	0.027559	0.079408	0.047202	IL2/LEP/ST	3
hsa05321	Inflammato	2/33	65/8108	0.028485	0.080707	0.047974	IL2/STAT1	2
hsa05225	Hepatocel	3/33	168/8108	0.030256	0.082867	0.049258	GSTM1/GS	3
hsa04010	MAPK sigr	4/33	294/8108	0.030306	0.082867	0.049258	IGF1R/INS	4
hsa04664	Fc epsilon	2/33	68/8108	0.030962	0.082867	0.049258	MAPK14/N	2
hsa05204	Chemical c	2/33	69/8108	0.031806	0.082867	0.049258	GSTM1/GS	2
hsa05164	Influenza /	3/33	172/8108	0.03213	0.082867	0.049258	CASP1/FD	3
hsa04622	RIG-I-like	2/33	70/8108	0.032659	0.082867	0.049258	MAPK14/N	2
hsa05120	Epithelial c	2/33	70/8108	0.032659	0.082867	0.049258	MAPK14/N	2
hsa04520	Adherens j	2/33	71/8108	0.033522	0.083804	0.049816	IGF1R/INS	2
hsa00982	Drug meta	2/33	72/8108	0.034393	0.084736	0.05037	GSTM1/GS	2
hsa01524	Platinum c	2/33	73/8108	0.035273	0.085663	0.050921	GSTM1/GS	2
hsa05212	Pancreatic	2/33	76/8108	0.037966	0.090904	0.054036	MAPK8/ST	2
hsa00980	Metabolisr	2/33	78/8108	0.039804	0.093982	0.055865	GSTM1/GS	2
hsa05167	Kaposi sar	3/33	194/8108	0.043504	0.101311	0.060222	MAPK14/N	3
hsa05169	Epstein-Ba	3/33	202/8108	0.048083	0.11046	0.065661	MAPK14/N	3

ID	Descriptio	GeneRatio	BgRatio	pvalue	p.adjust	qvalue	geneID	Count
hsa04068	FoxO signa	8/33	131/8108	3.71E-08	6.30E-06	3.74E-06	IGF1R/INS	8
hsa04920	Adipocyto	6/33	69/8108	2.81E-07	2.30E-05	1.37E-05	IRS1/LEP/N	6
hsa04152	AMPK sign	7/33	120/8108	4.05E-07	2.30E-05	1.37E-05	IGF1R/INS	7
hsa05418	Fluid shea	7/33	139/8108	1.10E-06	4.38E-05	2.60E-05	GSTM1/GS	7
hsa04211	Longevity	6/33	89/8108	1.29E-06	4.38E-05	2.60E-05	IGF1R/INS	6
hsa04657	IL-17 signa	6/33	94/8108	1.78E-06	4.87E-05	2.90E-05	HSP90AA1	6
hsa05215	Prostate ca	6/33	97/8108	2.14E-06	4.87E-05	2.90E-05	GSTP1/HS	6
hsa04932	Non-alcoh	7/33	155/8108	2.29E-06	4.87E-05	2.90E-05	INSR/IRS1/	7
hsa05171	Coronaviru	8/33	232/8108	2.98E-06	5.63E-05	3.35E-05	CASP1/F2/	8
hsa04213	Longevity	5/33	62/8108	4.47E-06	7.59E-05	4.51E-05	IGF1R/INS	5
hsa05417	Lipid and a	7/33	215/8108	1.99E-05	0.000307	0.000182	CASP1/HS	7
hsa04625	C-type lec	5/33	104/8108	5.62E-05	0.000797	0.000474	CASP1/IL2	5
hsa04659	Th17 cell c	5/33	107/8108	6.44E-05	0.000818	0.000486	HSP90AA1	5
hsa04931	Insulin resi	5/33	108/8108	6.74E-05	0.000818	0.000486	INSR/IRS1/	5
hsa05415	Diabetic ca	6/33	203/8108	0.000144	0.001632	0.00097	INSR/IRS1/	6
hsa04926	Relaxin sig	5/33	129/8108	0.000157	0.001664	0.000989	MAPK14/N	5
hsa04917	Prolactin s	4/33	70/8108	0.000173	0.001726	0.001026	CYP17A1/I	4
hsa04910	Insulin sign	5/33	137/8108	0.000208	0.001961	0.001166	INSR/IRS1/	5
hsa05133	Pertussis	4/33	76/8108	0.000237	0.002019	0.0012	CASP1/MA	4
hsa04140	Autophagy	5/33	141/8108	0.000238	0.002019	0.0012	IGF1R/IRS1	5
hsa04150	mTOR sign	5/33	155/8108	0.000369	0.002984	0.001774	IGF1R/INS	5
hsa04151	PI3K-Akt s	7/33	354/8108	0.000455	0.003517	0.00209	HSP90AA1	7
hsa04658	Th1 and Th	4/33	92/8108	0.000494	0.00365	0.00217	IL2/MAPK1	4
hsa01522	Endocrine	4/33	98/8108	0.000628	0.004445	0.002642	IGF1R/MA	4
hsa04914	Progesterc	4/33	100/8108	0.000677	0.004606	0.002738	HSP90AA1	4
hsa05142	Chagas dis	4/33	102/8108	0.00073	0.004719	0.002805	IL2/MAPK1	4
hsa04621	NOD-like	5/33	181/8108	0.000749	0.004719	0.002805	CASP1/HS	5
hsa04620	Toll-like re	4/33	104/8108	0.000785	0.004768	0.002834	CTSK/MAF	4
hsa04930	Type II dia	3/33	46/8108	0.000828	0.004852	0.002884	INSR/IRS1/	3
hsa04668	TNF signal	4/33	112/8108	0.001037	0.005685	0.003379	MAPK14/N	4
hsa05145	Toxoplasm	4/33	112/8108	0.001037	0.005685	0.003379	MAPK14/N	4
hsa04913	Ovarian st	3/33	51/8108	0.00112	0.00595	0.003537	CYP17A1/I	3
hsa04935	Growth ho	4/33	119/8108	0.001299	0.006693	0.003978	IRS1/MAPI	4
hsa04380	Osteoclast	4/33	128/8108	0.001701	0.008505	0.005056	CTSK/MAF	4
hsa05135	Yersinia in	4/33	137/8108	0.002182	0.010601	0.006301	CASP1/IL2	4
hsa05140	Leishmania	3/33	77/8108	0.003661	0.01719	0.010218	MAPK14/N	3
hsa04217	Necroptos	4/33	159/8108	0.003741	0.01719	0.010218	CASP1/HS	4
hsa05161	Hepatitis B	4/33	162/8108	0.004	0.017777	0.010567	MAPK14/N	4
hsa00983	Drug meta	3/33	80/8108	0.004078	0.017777	0.010567	GSTM1/GS	3
hsa04530	Tight junct	4/33	169/8108	0.004651	0.019768	0.011751	MAPK8/PR	4
hsa04610	Compleme	3/33	85/8108	0.004835	0.02005	0.011918	F10/F2/PL/	3
hsa05152	Tuberculos	4/33	180/8108	0.005814	0.023531	0.013988	MAPK14/N	4
hsa05323	Rheumato	3/33	93/8108	0.006214	0.024568	0.014604	CTSK/MMI	3
hsa04710	Circadian r	2/33	31/8108	0.006939	0.02681	0.015936	PRKAA2/P	2
hsa05202	Transcripti	4/33	192/8108	0.007288	0.027533	0.016366	IGF1R/MM	4
hsa04933	AGE-RAGE	3/33	100/8108	0.007596	0.028071	0.016686	MAPK14/N	3
hsa05130	Pathogeni	4/33	197/8108	0.007969	0.028826	0.017135	CASP1/F2/	4
hsa04660	T cell rece	3/33	104/8108	0.00846	0.029964	0.017812	IL2/MAPK1	3
hsa05205	Proteoglyc	4/33	205/8108	0.009145	0.031729	0.01886	IGF1R/MA	4
hsa04066	HIF-1 sign	3/33	109/8108	0.00962	0.032628	0.019395	IGF1R/INS	3
hsa04960	Aldosteror	2/33	37/8108	0.009788	0.032628	0.019395	INSR/IRS1	2
hsa05219	Bladder ca	2/33	41/8108	0.01193	0.039003	0.023185	MMP1/MN	2
hsa04722	Neurotrop	3/33	119/8108	0.012207	0.039156	0.023275	IRS1/MAPI	3
hsa05132	Salmonella	4/33	249/8108	0.017654	0.054982	0.032683	CASP1/HS	4
hsa04371	Apelin sign	3/33	137/8108	0.017788	0.054982	0.032683	NOS2/PRK	3
hsa05162	Measles	3/33	139/8108	0.018483	0.056109	0.033353	IL2/MAPK8	3
hsa00480	Glutathion	2/33	57/8108	0.022301	0.065366	0.038855	GSTM1/GS	2
hsa04923	Regulation	2/33	57/8108	0.022301	0.065366	0.038855	INSR/IRS1	2
hsa04630	JAK-STAT	3/33	162/8108	0.027559	0.079408	0.047202	IL2/LEP/ST	3
hsa05321	Inflammat	2/33	65/8108	0.028485	0.080707	0.047974	IL2/STAT1	2
hsa05225	Hepatocel	3/33	168/8108	0.030256	0.082867	0.049258	GSTM1/GS	3
hsa04010	MAPK sign	4/33	294/8108	0.030306	0.082867	0.049258	IGF1R/INS	4
hsa04664	Fc epsilon	2/33	68/8108	0.030962	0.082867	0.049258	MAPK14/N	2
hsa05204	Chemical c	2/33	69/8108	0.031806	0.082867	0.049258	GSTM1/GS	2
hsa05164	Influenza A	3/33	172/8108	0.03213	0.082867	0.049258	CASP1/FD	3
hsa04622	RIG-I-like	2/33	70/8108	0.032659	0.082867	0.049258	MAPK14/N	2
hsa05120	Epithelial c	2/33	70/8108	0.032659	0.082867	0.049258	MAPK14/N	2
hsa04520	Adherens j	2/33	71/8108	0.033522	0.083804	0.049816	IGF1R/INS	2
hsa00982	Drug meta	2/33	72/8108	0.034393	0.084736	0.05037	GSTM1/GS	2
hsa01524	Platinum c	2/33	73/8108	0.035273	0.085663	0.050921	GSTM1/GS	2
hsa05212	Pancreatic	2/33	76/8108	0.037966	0.090904	0.054036	MAPK8/ST	2
hsa00980	Metabolisr	2/33	78/8108	0.039804	0.093982	0.055865	GSTM1/GS	2
hsa05167	Kaposi sarc	3/33	194/8108	0.043504	0.101311	0.060222	MAPK14/N	3
hsa05169	Epstein-Ba	3/33	202/8108	0.048083	0.11046	0.065661	MAPK14/N	3

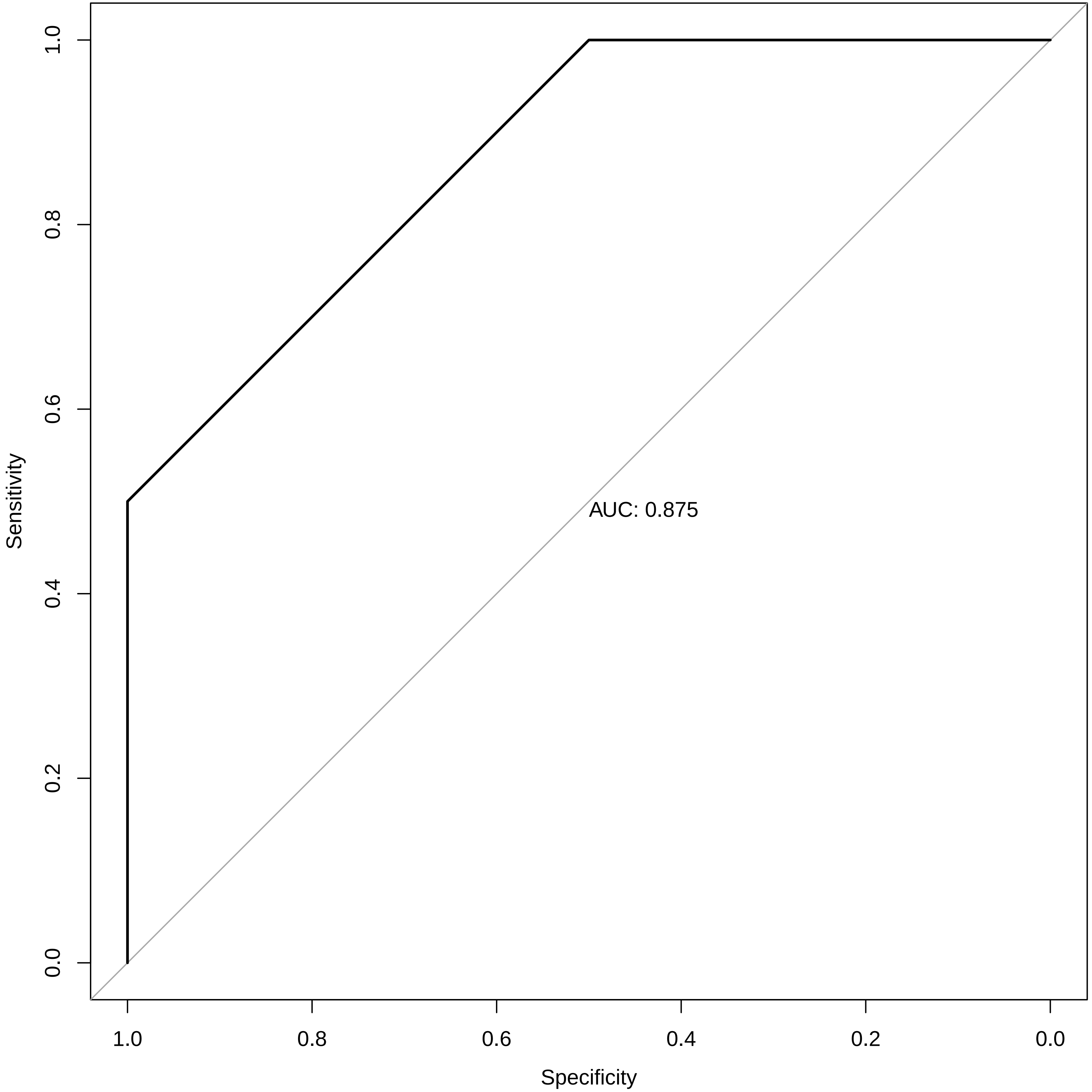

AUC: 0.875
Sensitivity
1.0
0.8
0.6
0.4
0.2
0.0
Specificity
1.0
0.8
0.6
0.4
0.2
0.0

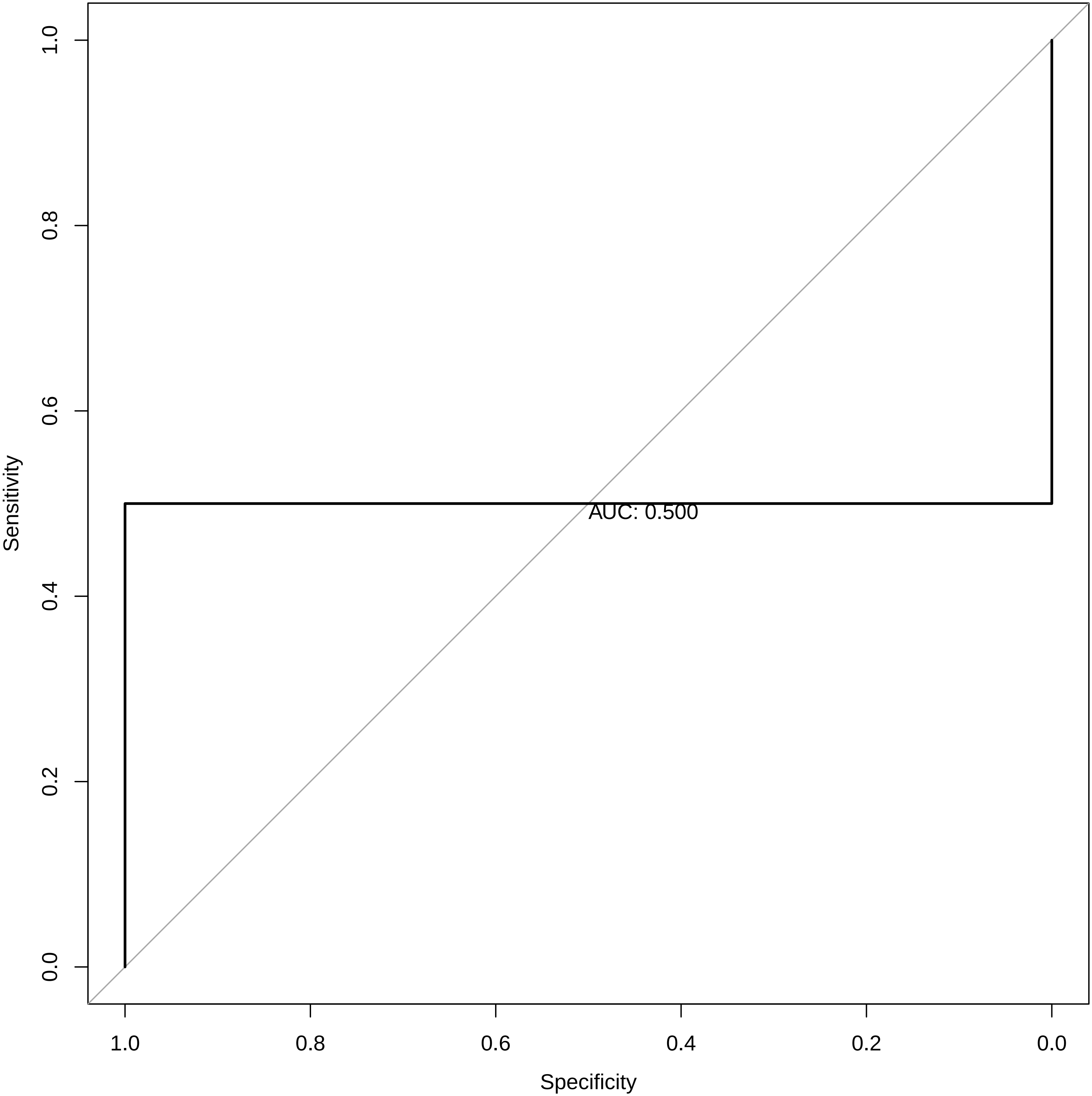

Sensitivity
1.0
0.8
0.6
0.4
0.2
0.0
AUC: 0.500
Specificity

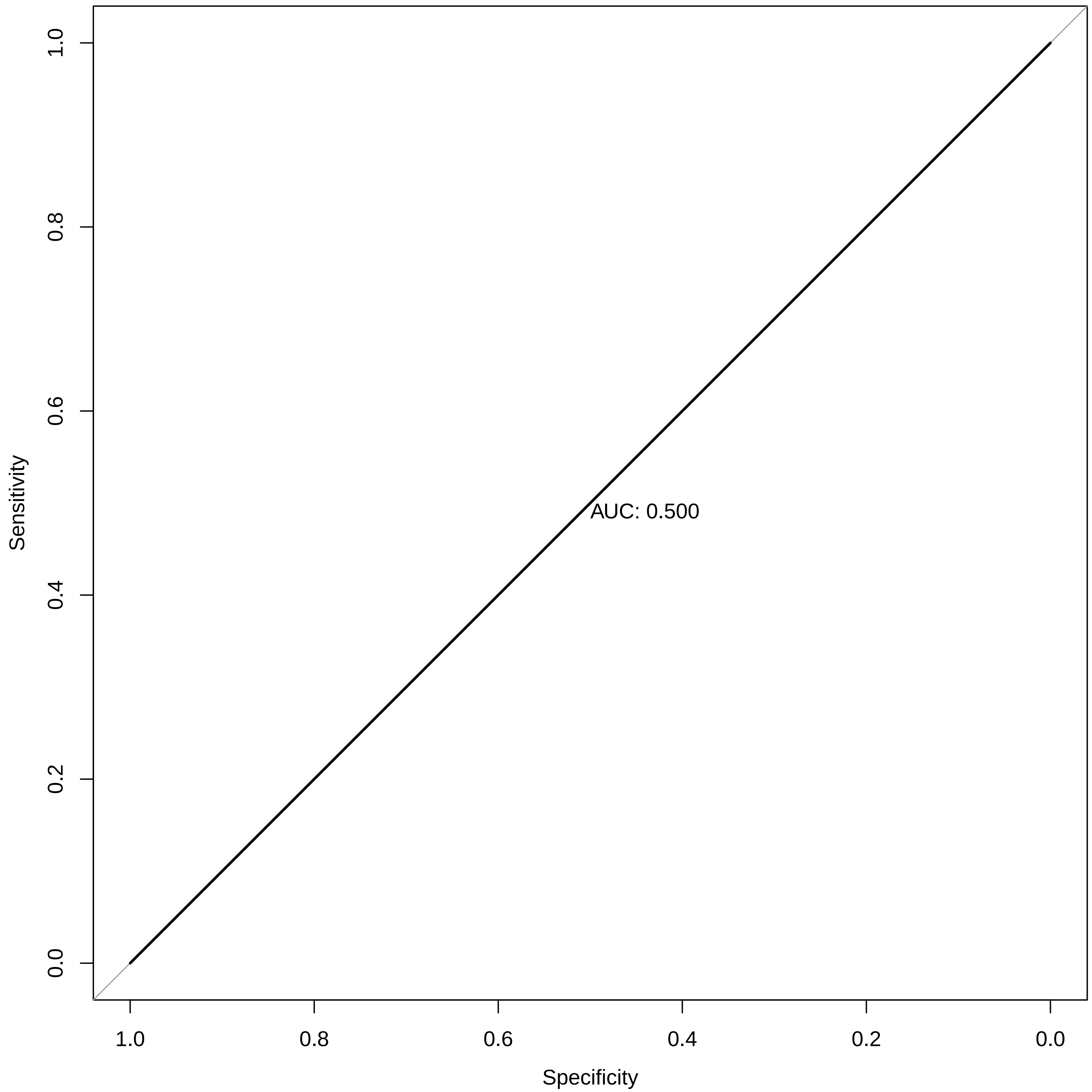

AUC: 0.500
Sensitivity
Specificity
1.0
0.8
0.6
0.4
0.2
0.0

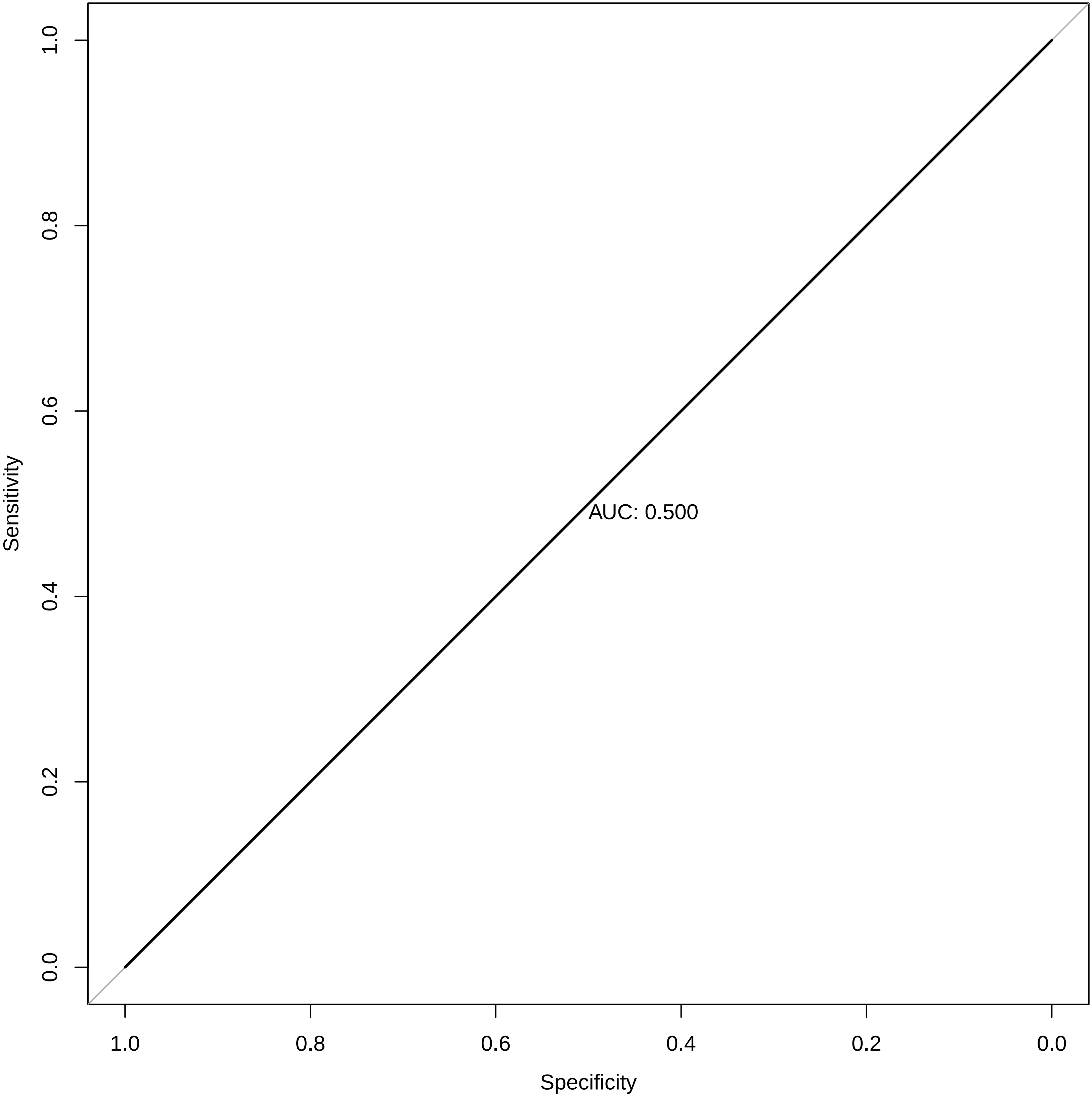

Sensitivity
1.0
0.8
0.6
0.4
0.2
0.0
AUC: 0.500
1.0
0.8
0.6
0.4
0.2
0.0
Specificity

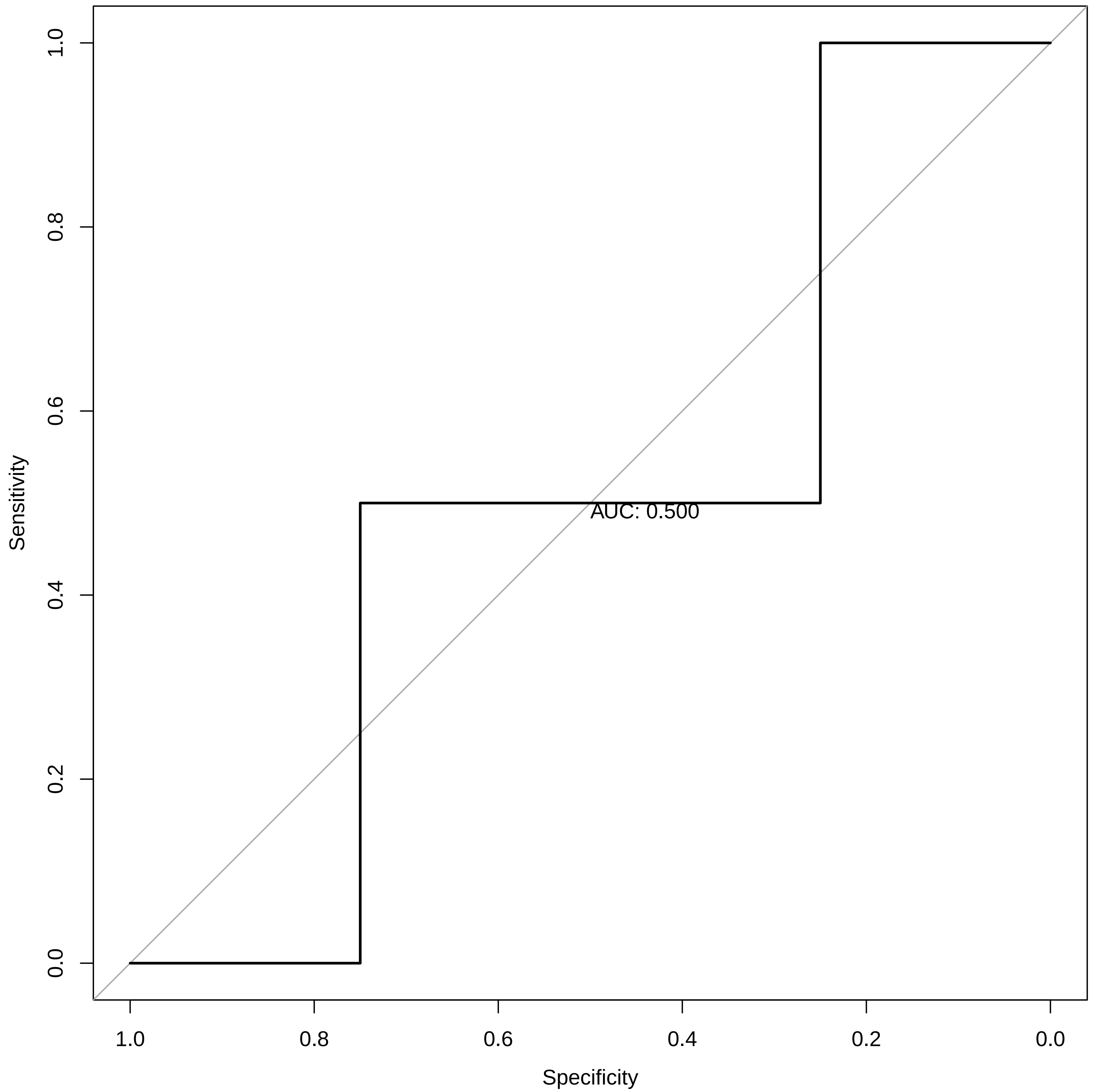
AUC: 0.500
Sensitivity
Specificity
0.0
0.2
0.4
0.6
0.8
1.0

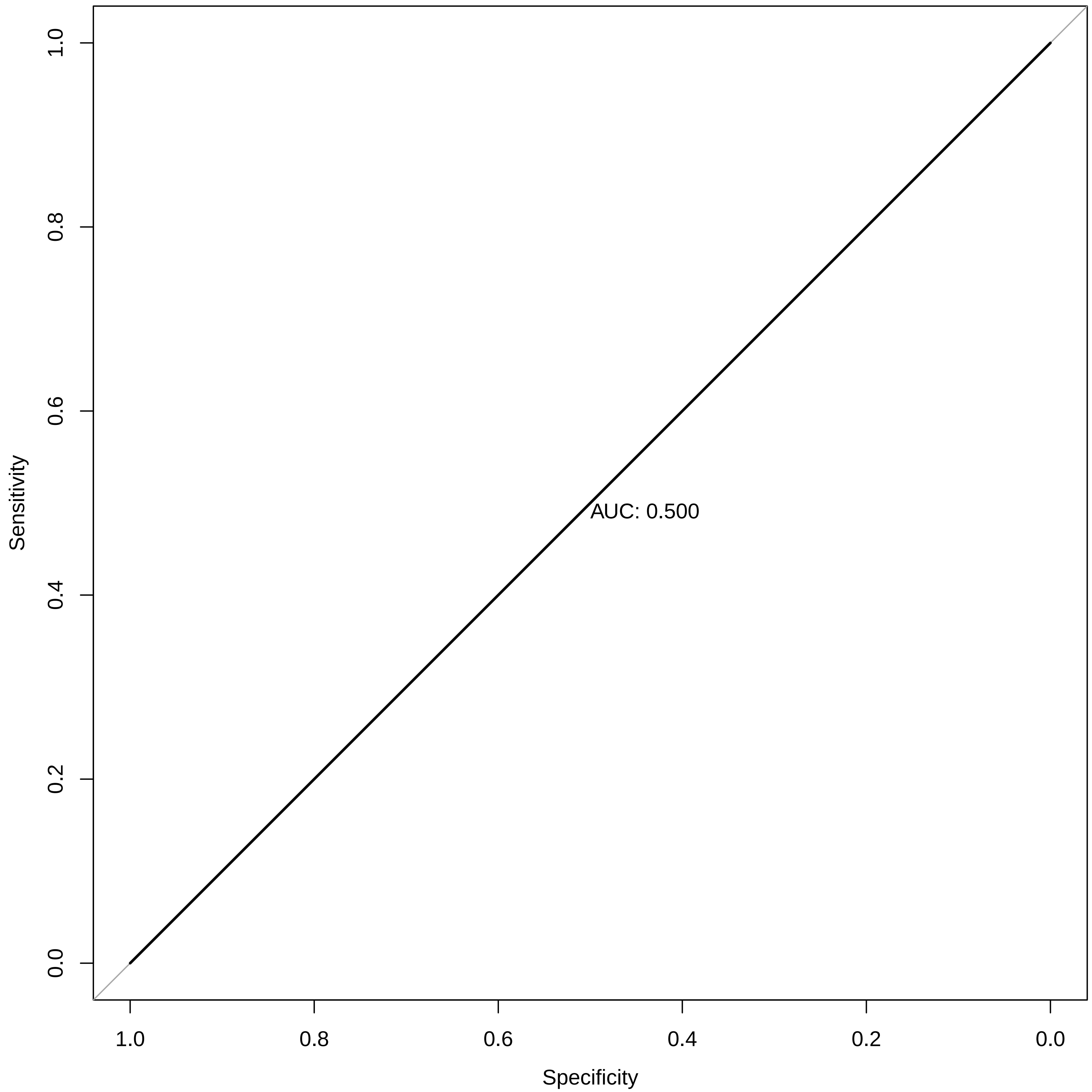

AUC: 0.500
Sensitivity
Specificity
1.0
0.8
0.6
0.4
0.2
0.0

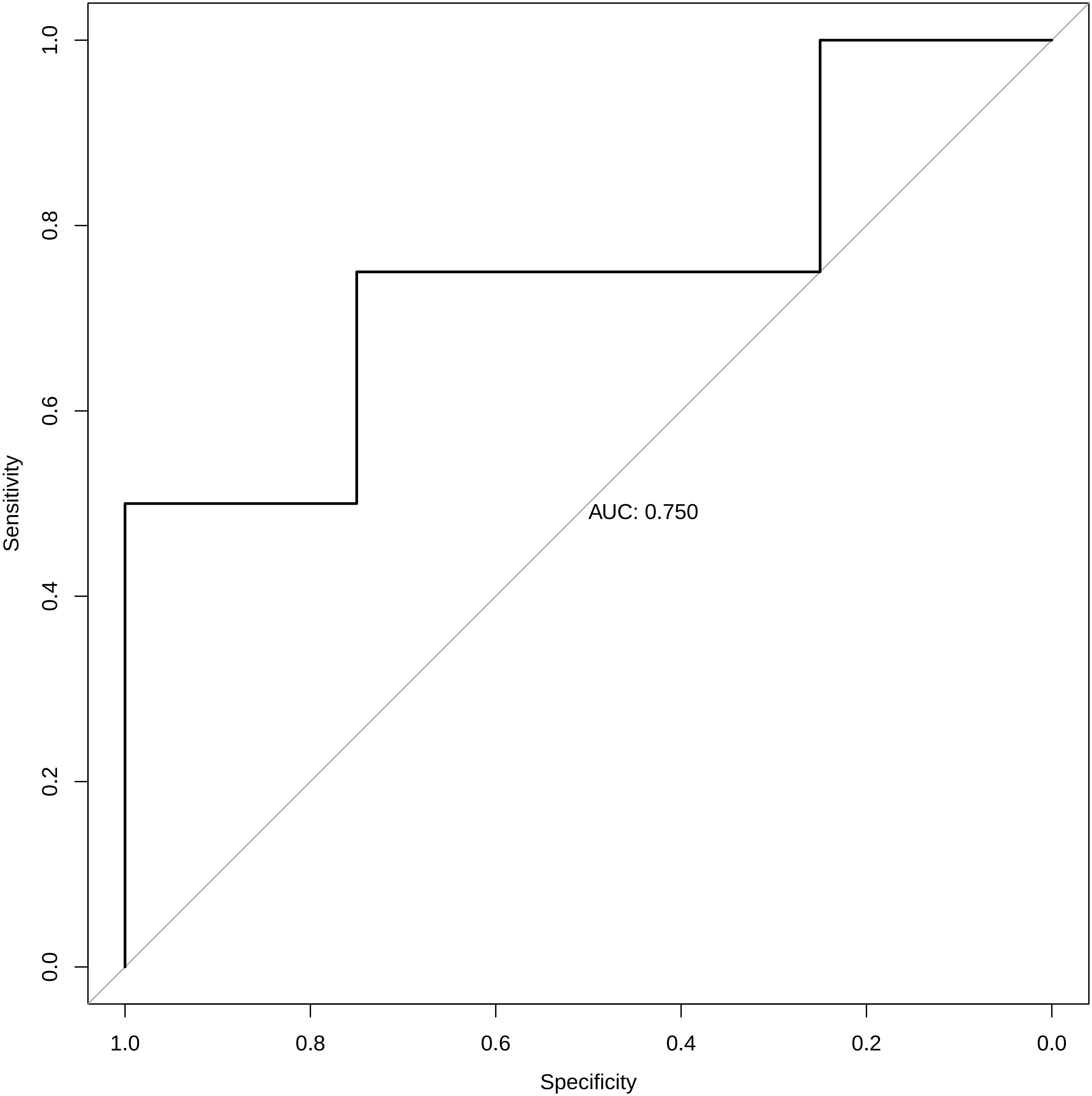

AUC: 0.750
Sensitivity
Specificity
1.0
0.8
0.6
0.4
0.2
0.0

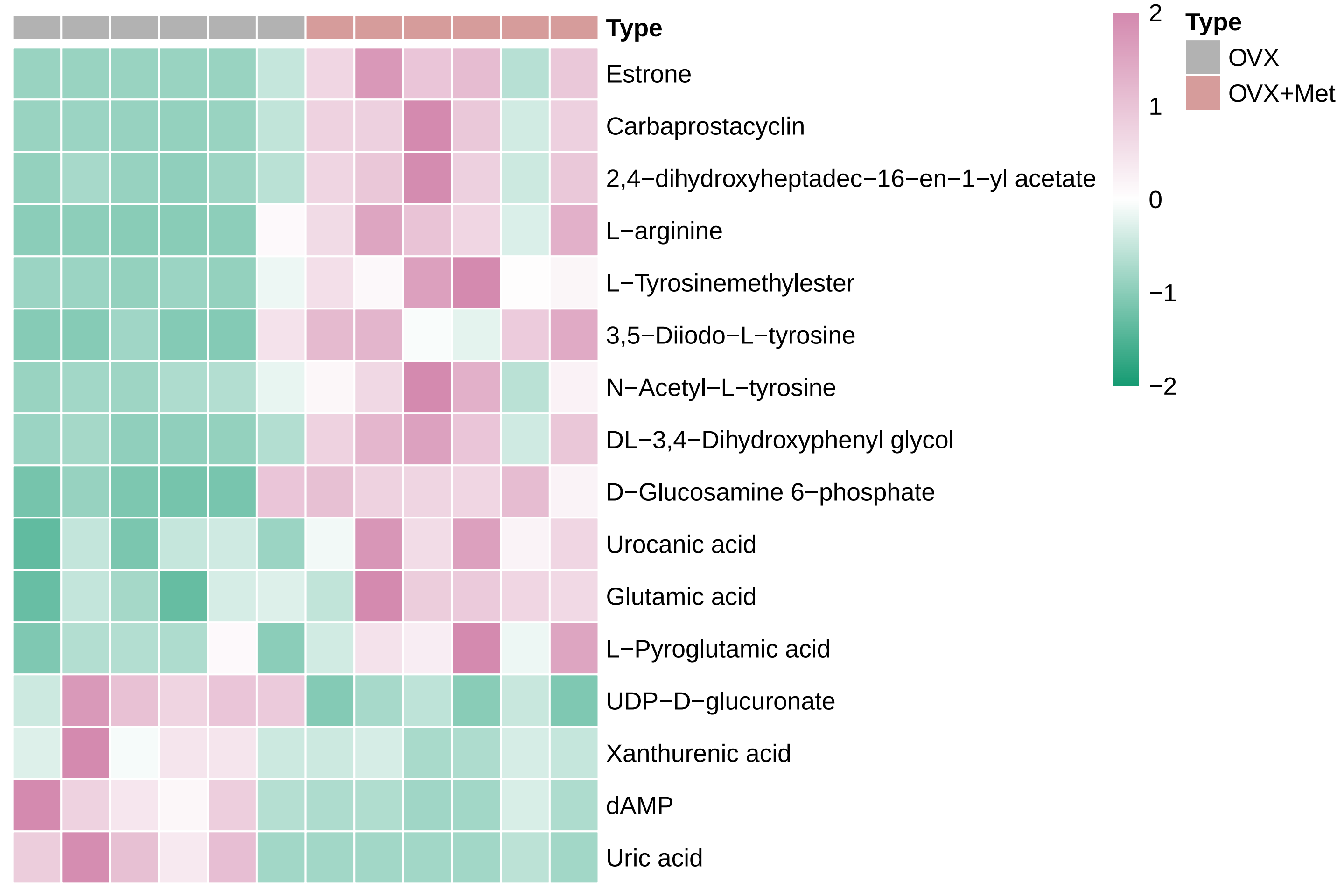
Type
Estrone
Carbaprostacyclin
2,4−dihydroxyheptadec−16−en−1−yl acetate
L−arginine
L−Tyrosinemethylester
3,5−Diiodo−L−tyrosine
N−Acetyl−L−tyrosine
DL−3,4−Dihydroxyphenyl glycol
D−Glucosamine 6−phosphate
Urocanic acid
Glutamic acid
L−Pyroglutamic acid
UDP−D−glucuronate
Xanthurenic acid
dAMP
Uric acid
2
1
0
−1
−2
Type
OVX
OVX+Met

purinediff

ID	Name	Formula	Molecular	RT [min]	m/z
Com_3048_pos	Carbaprostacyclin	C21 H34 C	332.2351	14.143	333.2425
Com_4975_neg	UDP-D-glucuronate	C15 H22 N	580.0346	1.426	579.0274
Com_1632_pos	Xanthurenic acid	C10 H7 N	205.0381	7.126	206.0454
Com_17330_pos	L-Tyrosinemethylester	C10 H13 N	195.0899	11.964	196.0972
Com_15124_pos	N-Acetyl-L-tyrosine	C11 H13 N	223.0847	12.275	224.0919
Com_18996_pos	3,5-Diiodo-L-tyrosine	C9 H9 I2 N	432.8685	8.279	433.8756
Com_19209_pos	D-Glucosamine 6-phosphate	C6 H14 N	259.0464	7.635	260.0537
Com_10368_pos	L-arginine	C6 H14 N4	174.112	8.311	175.1193
Com_2048_pos	Glutamic acid	C5 H9 N C	147.0533	1.325	148.0605
Com_928_pos	L-Pyroglutamic acid	C5 H7 N C	129.0428	2.127	130.0502
Com_1684_pos	2,4-dihydroxyheptadec-16-en-	C19 H36 C	350.2457	14.14	351.2519
Com_15327_pos	DL-3,4-Dihydroxyphenyl glycol	C8 H10 O4	170.0586	7.217	171.0659
Com_5387_pos	Estrone	C18 H22 C	287.1849	10.437	288.1921
Com_1363_pos	Uric acid	C5 H4 N4	168.0286	1.812	169.0359
Com_7034_pos	dAMP	C10 H14 N	331.0677	6.951	332.075
Com_1323_pos	Urocanic acid	C6 H6 N2	138.0431	1.743	139.0505
Com_4975_neg	UDP-D-glucuronate	C15 H22 N	580.0346	1.426	579.0274

mzCloud_I	mzVault_R	MassList_R	E1	E2	E3	E4	E5	E6
Invalid ma	No results	No match	26010575	26679956	44920314	28889128	9255942	26620924
No results	No results	Full match	778716.6	986257	1132197	798899.8	1191586	741877
Full match	No results	Full match	2028544	2573842	236498.3	468847.3	2618215	1768635
No results	No results	Full match	974483.7	721867.1	1603377	1859599	683486	753847.1
No results	No results	Full match	972536.4	1406855	2769641	1956174	391371.6	1049637
Full match	No results	Full match	1100722	1144340	521169.9	432864	959460	1228974
No results	No results	Full match	993901.3	862718.7	847832.1	838590.3	1019053	630486.3
No results	No results	Full match	3761152	5781538	4610497	3952178	1774112	5306196
No results	Full match	Full match	30146167	62143358	47579077	48089359	45589714	44758247
Full match	Full match	Full match	66327033	89373725	84044486	1.34E+08	72611194	1.17E+08
Invalid ma	No results	No match	58933641	67316641	1.01E+08	61952630	20940978	66564490
No results	No results	Full match	1945049	2372086	2681308	2133211	906137.3	2118883
Invalid ma	No results	No results	10255485	17260798	12052948	13114013	1786308	11838346
Full match	No results	Full match	1422304	1404444	1422781	1281502	12912580	1387563
No results	No results	Full match	315311	348893.8	96871.49	129108.8	962544.9	319710.4
Full match	Full match	Full match	47671825	97134502	66005171	92814711	55561565	68807618
No results	No results	Full match	778716.6	986257	1132197	798899.8	1191586	741877

M1	M2	M3	M4	M5	M6	FC	log2FC	Pvalue
2051365	2225914	1777940	1462543	1952988	7156182	9.765893	3.287752	2.17E-05
1216587	2763926	2272084	2039927	2223234	2155898	0.444262	-1.17052	0.000268
2908288	23363781	4411402	7481389	7504497	2147617	0.202744	-2.30227	0.01537
167997.8	171005	135033.5	165342.3	134604.2	580912.2	4.868761	2.283555	0.000215
163743.4	225490.7	188745.7	306809.4	345796.8	715467.9	4.391562	2.134734	0.002367
54909.11	54453.26	157105.4	46237.59	44036.62	779532.1	4.7414	2.245313	0.003886
63117.53	174007.5	84592.84	57957.63	66247.75	952113	3.714197	1.89305	0.006591
269654.6	308938.4	244882	214069.2	311829.2	2613667	6.355139	2.667924	0.000951
20411696	30465404	27103161	20101667	32504725	33231003	1.698876	0.764581	0.002587
47739055	59509803	59486187	58459576	78911472	50591153	1.58752	0.666775	0.008949
4803130	10024270	5235053	3134670	6976826	15149893	8.302524	3.05355	4.51E-05
508777.6	582453.8	410664.7	413556.4	454404	689606.1	3.973467	1.990398	8.41E-05
92862.64	109615.5	85806.78	87687.91	89838.23	2517216	22.22839	4.474332	0.000212
87539922	1.45E+08	98996880	62525582	1E+08	1168881	0.040043	-4.6423	0.006369
6196018	2939871	2327825	1835755	3111131	418777.1	0.129086	-2.95359	0.001992
15031739	37218109	21052466	37856804	39800690	28141428	2.389684	1.25682	0.001323
1216587	2763926	2272084	2039927	2223234	2155898	0.444262	-1.17052	0.000268

ROC	VIP	Up_Down
1	1.405537	up
1	1.359267	down
0.944444	1.070892	down
1	1.345612	up
0.972222	1.222848	up
0.944444	1.239491	up
0.888889	1.245287	up
0.972222	1.330832	up
0.916667	1.207325	up
0.944444	1.183849	up
1	1.385493	up
1	1.408431	up
0.972222	1.366729	up
0.833333	1.226635	down
0.972222	1.237016	down
1	1.308189	up
1	1.359267	down
